# Fluorescence umpolung enables light-up sensing of *N*-acetyltransferases and nerve agents

**DOI:** 10.1038/s41467-021-24187-5

**Published:** 2021-06-23

**Authors:** Chenxu Yan, Zhiqian Guo, Weijie Chi, Wei Fu, Syed Ali Abbas Abedi, Xiaogang Liu, He Tian, Wei-Hong Zhu

**Affiliations:** 1 Key Laboratory for Advanced Materials and Joint International Research Laboratory of Precision Chemistry and Molecular Engineering, Shanghai Key Laboratory of Functional Materials Chemistry, Frontiers Science Center for Materiobiology and Dynamic Chemistry, Institute of Fine Chemicals, School of Chemistry and Molecular Engineering, East China University of Science & Technology, Shanghai, China; 2 Fluorescence Research Group, Singapore University of Technology and Design, Singapore, Singapore

**Keywords:** Fluorescent dyes, Fluorescent probes

## Abstract

Intramolecular charge transfer (ICT) is a fundamental mechanism that enables the development of numerous fluorophores and probes for bioimaging and sensing. However, the electron-withdrawing targets (EWTs)-induced fluorescence quenching is a long-standing and unsolved issue in ICT fluorophores, and significantly limits the widespread applicability. Here we report a simple and generalizable structural-modification for completely overturning the intramolecular rotation driving energy, and thus fully reversing the ICT fluorophores’ quenching mode into light-up mode. Specifically, the insertion of an indazole unit into ICT scaffold can fully amplify the intramolecular rotation in donor-indazole-π-acceptor fluorophores (fluorescence OFF), whereas efficiently suppressing the rotation in their EWT-substituted system (fluorescence ON). This molecular strategy is generalizable, yielding a palette of chromophores with fluorescence umpolung that spans visible and near-infrared range. This strategy expands the bio-analytical toolboxes and allows exploiting ICT fluorophores for light-up sensing of EWTs including *N*-acetyltransferases and nerve agents.

## Abstract (web summary)

The electron-withdrawing target (EWT)-induced fluorescence quenching is an unsolved issue in intramolecular charge transfer (ICT) fluorophores that limits their applicability. Here, the authors report a simple and generalizable strategy to reverse the EWT-induced quenching mode into light-up mode, by introducing an indazole building block between the π-bridge and the donor in the ICT scaffold.

## Introduction

**Fig. 1:**
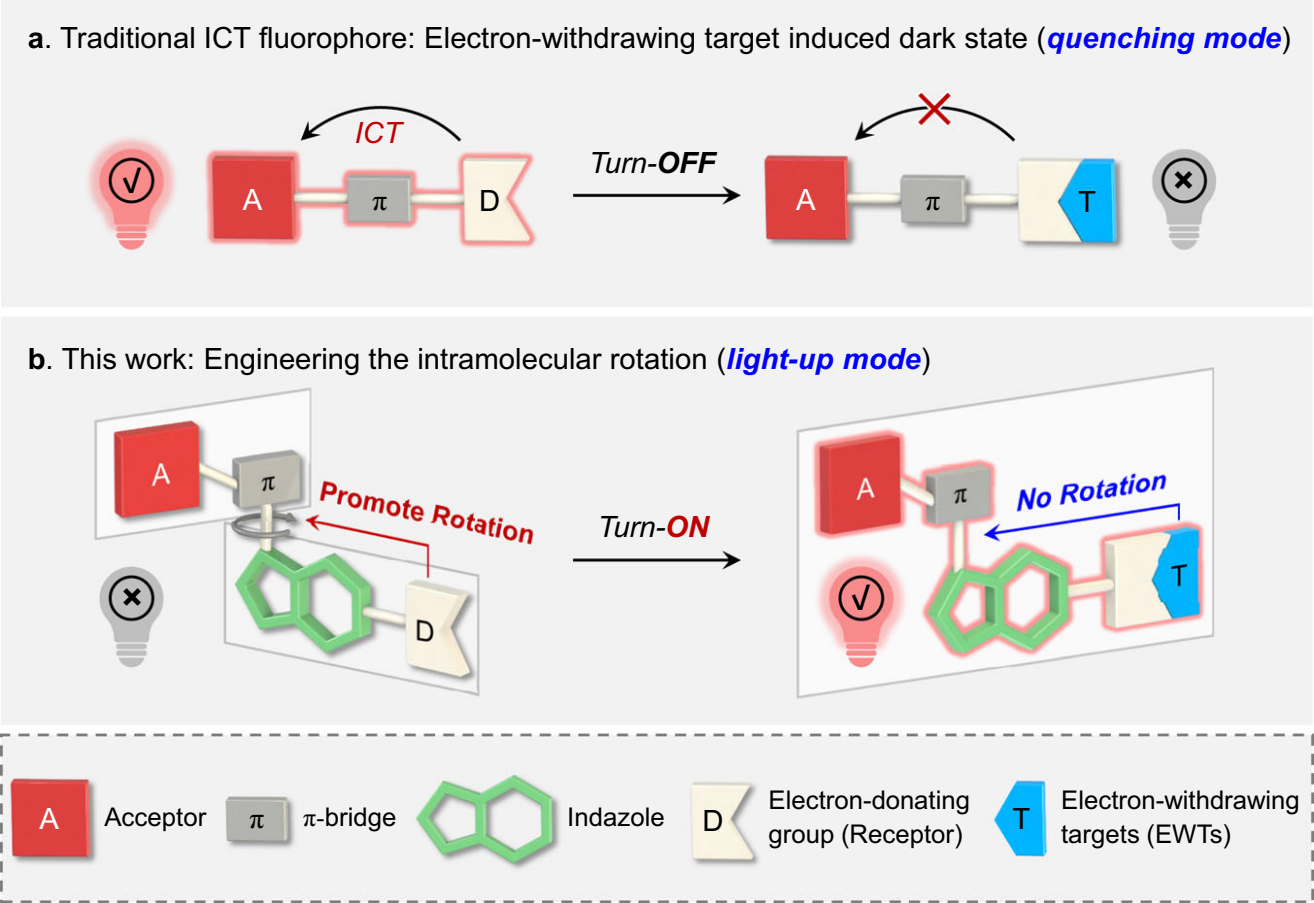
Fluorescence umpolung of ICT fluorophores: overturning the quenching mode into light-up mode. **a** Traditional ICT fluorophores: electron-withdrawing targets (EWTs)-induced fluorescence quenching. **b** In this work, indazole-based ICT fluorophores enable light-up sensing of EWTs: D-indazole-π-A fluorophores show obvious intramolecular rotation between indazole and π-bridge, resulting in a dark state (rotation-induced quenching, fluorescence OFF). Upon the incorporation of EWTs (such as *N*-acetyltransferases and nerve agents), the resulting EWT-indazole-π-A fluorophores show no rotation in the photophysical process, leading to a significant fluorescence enhancement (fluorescence ON).

Intramolecular charge transfer (ICT)-based fluorophores^1,2^, featured by donor-π-acceptor (D-π-A) scaffold, are essential research tools in biosensing^3–7^ and bioimaging^8–12^. Their exceptionally high sensitivity to the electron disturbance and large Stokes shifts make these chromophores promising platforms for the construction of numerous high-performance fluorescent dyes and probes^13–17^. In general, the substitution and/or interaction of the donor receptor with electron-withdrawing targets (EWTs, which are widespread and found in living organisms and natural environments) can intrinsically suppress the ICT pathway, thereby undesirably quenching the fluorescence (Fig. 1a)^18–20^. This quenching mode of ICT fluorophores inevitably generates misleading information and severely restricts their applicability for accurate sensing/labeling. Therefore, it is essential to formulate a molecular design strategy that overcomes the essential issue of the EWT-induced fluorescence quenching, which will pave a reliable pathway for the light-up and highly accurate analysis toolbox.

Herein, we report a simple and generalizable molecular engineering strategy—insertion of an indazole building block into ICT chromophore to regulate the intramolecular rotation driving energy—for completely overturning the EWT-induced quenching mode into the light-up mode (Fig. 1b). Starting from D-π-A chromophore like a typical laser dye dicyanomethylene-4H-pyran (DCM)^21,22^, we further expand this fluorescence umpolung strategy to other D-π-A featured chromophores and other insertion building blocks (benzo five-membered heterocycles, such as benzothiophene) that span the visible and near-infrared (NIR) range (550–700 nm). As demonstrated, we show that the electron density perturbation of the insertion building block can lead to a mutational effect on intramolecular rotation driving energy (Δ*E*_RDE_), flipping between positive (enhance rotation) and negative (suppress rotation). Specifically, the straightforward insertion of an indazole building block into the D-π-A motif can (i) substantially increase the intramolecular rotation in D-indazole-π-A fluorophores (Δ*E*_RDE_ > 0), resulting in a rotation-induced dark state, whereas (ii) efficiently suppress the rotation with the incorporation of EWTs (Δ*E*_RDE_ < 0), along with a significant fluorescence enhancement. This fluorescence umpolung strategy expands the understanding of ICT mechanism, and allows us to elaborately design ICT probes for light-up sensing of EWTs, like *N*-acetyltransferases and nerve agents.

## Results

### Engineering ICT chromophores for fluorescence umpolung

**Fig. 2:**
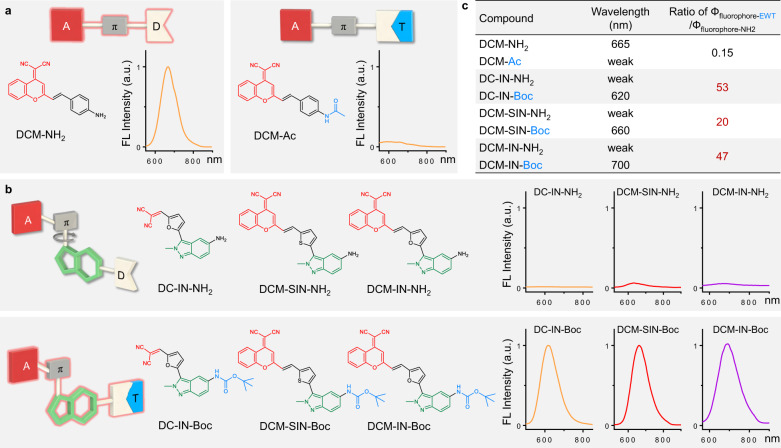
Fluorescence umpolung of indazole-based fluorophores. **a** Fluorescence spectra of DCM-NH_2_ (10 μM) and its electron-withdrawing acetyl-substituted product DCM-Ac (10 μM) in DMSO. **b** Design of indazole-based fluorophores: starting with D-π-A fluorophore as the motif; inserting an indazole building block between the π-bridge and donor. Fluorescence spectra of these indazole-based fluorophores indicated their fluorescence umpolung properties. The D-indazole-π-A type dyes displayed weak emission intensity, whereas their electron-withdrawing Boc-substituted products (EWT-indazole-π-A type dyes) exhibited strong fluorescence. Fluorescence spectra of each pair of the amino-substituted fluorophore and its EWT-substituted fluorophore were recorded in the same test condition (10 μM, in DMSO). **c** Emission wavelength and EWT-induced enhancement of fluorescence quantum yield in solution-state (the ratio of Ф_fluorophore-EWT_/Ф_fluorophore-NH2_).

Aiming to expand the utility of ICT fluorophores along with tailoring their emission properties for high-fidelity bioimaging in vivo, our group has been engaged in a long-term research project investigating the use of high-performance donor-π-acceptor (D-π-A) fluorescent dyes, such as dicyanomethylene-4H-pyran (DCM)^23–25^, quinoline-malononitrile (QM)^26,27^, and so on. To further extend the emission wavelength and optimize the biocompatibility, we grafted an indazole unit between the π-bridge and donor in the D-π-A motif because of indazole’s biological and pharmacological activities^28^ (Fig. 2 and Supplementary Figs. 1–3). In the designed D-indazole-π-A fluorophore named DCM-IN-NH_2_, the amino unit is employed as the donor, furan as the π-bridge, and DCM as the acceptor. To study the effects of the electron density perturbation on emission, we then substituted the electron-donating amino group with an electron-withdrawing *tert*-butoxycarbonyl unit (Boc) to obtain an EWT-indazole-π-A fluorophore named DCM-IN-Boc. Unexpectedly, we observed that the DCM-IN-Boc fluorophore with an electron-withdrawing Boc unit showed strong NIR emission (Fig. 2), thereby motivating us to more carefully study this fluorescence umpolung phenomenon.

As mentioned above, the EWT-induced fluorescence quenching is ubiquitous in ICT fluorophores, such as DCM-NH_2_ (fluorescence ON) and its acylate product DCM-Ac (fluorescence OFF) (Fig. 2a). However, upon the insertion of an additional indazole group into the D-π-A fluorophore, the resulting DCM-IN-NH_2_ (D-indazole-π-A type dye) shows unexpected very weak fluorescence. After further attachment of the amino group with an electron-withdrawing Boc group, the corresponding DCM-IN-Boc (EWT-indazole-π-A type dye) shows a bright fluorescence (120-fold enhancement) at around 700 nm (Fig. 2b and Supplementary Fig. 4). In this case, the insertion of indazole into DCM fluorophore can quench the fluorescence in the D-indazole-π-A featured system, but lighting-up the fluorescence in the EWT-indazole-π-A motif. Clearly, this EWT-induced light-up emission is completely opposite to the EWT-induced quenching mode of general ICT fluorophores and warranted further investigation.

Starting from the initial DCM-IN-NH_2_ and DCM-IN-Boc, we engineered a series of fluorescence umpolung chromophores using the following guidelines (Fig. 2b and Supplementary Fig. 4): (i) starting with D-π-A fluorophore as the motif; (ii) inserting an indazole building block between the π-bridge and donor. This strategy unlocks a great opportunity to rapidly establish a library of indazole-based fluorophores for studying the regularity of fluorescence umpolung. Specifically, we employed DCM, malononitrile, or aldehyde group as the acceptor (A), thiophene or furan as the π-bridge (π), 2-methyl-indazole or 1-methyl-indazole as the indazole building block (IN), amino group as the donor (D), and Boc or acetyl group as the EWT (Supplementary Fig. 4). To our delight, all resulting D-indazole-π-A chromophores displayed weak emission intensity, whereas their corresponding Boc-substituted products (EWT-indazole-π-A chromophores) exhibited strong fluorescence (Fig. 2b, c and Supplementary Fig. 4). Compiling these results together, we have successfully developed a simple and generalizable molecular engineering strategy—insertion of indazole building block into ICT chromophore—to achieve the fluorescence umpolung that spans the visible and NIR range.

### Reversing intramolecular rotation driving energy enables fluorescence umpolung

**Fig. 3:**
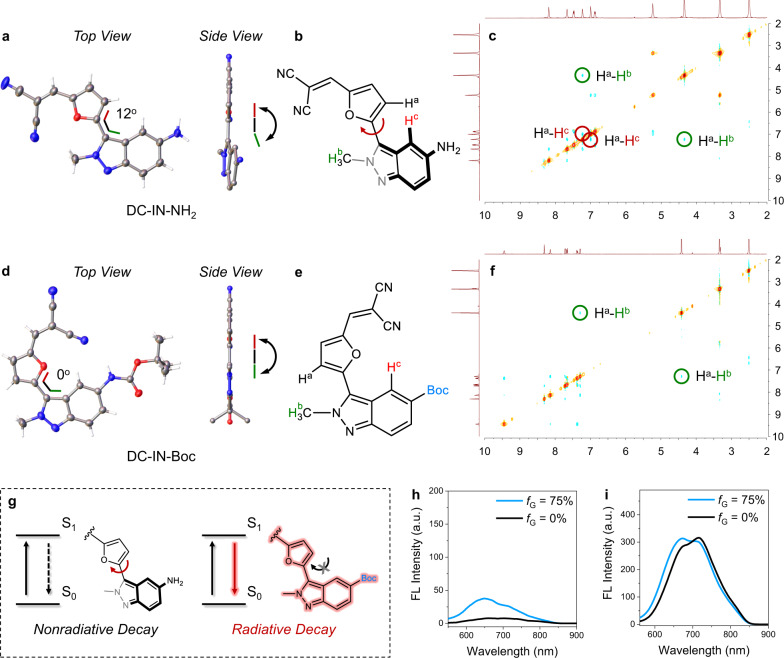
Modulation of intramolecular rotation between indazole and the π-bridge. **a**–**f** Single-crystal X-ray structures and 2D-NOESY NMR spectra (in DMSO-d_6_) of DC-IN-NH_2_ (**a**–**c**) and DC-IN-Boc (**d**–**f**). **g** Schematic illustration of EDG/EWT controlled intramolecular rotation for regulating the fluorescent intensity. **h**, **i** Emission spectra of DC-IN-NH_2_ (**h**) and DCM-IN-Boc (**i**) in a mixture of ethanol-glycerin with different glycerin volume fractions (*f*_G_), *λ*_ex_ = 525 nm.

To get a deeper understanding of this fluorescence umpolung, it’s critical to obtain the molecular geometries of these indazole-based chromophores. Fortunately, we acquired single crystals of IN-NH_2_, IN-Boc, IN1-NH_2_, DC-IN-NH_2_, DC-IN-Boc, and DCM-IN-NH_2_ (Fig. 3a, d and Supplementary Fig. 5, Supplementary Tables 1–6). Specifically, the amino-substituted chromophore DC-IN-NH_2_ (D-indazole-π-A type dye) shows an obvious dihedral angle (around 12°) between indazole and furan (Fig. 3a). After attachment of the amino group with an electron-withdrawing Boc unit, the resulting DC-IN-Boc (EWT-indazole-π-A type dye) exhibits an exactly coplanar conformation with 0° dihedral angle (Fig. 3d). According to the single-crystal analysis, the dihedral angle between indazole and the π-bridge is distinctly different from the electron density perturbation of indazole’s C_4_ position, and the molecular geometries in the solution-state should be further investigated.

To support the discrete molecular geometries of D-indazole-π-A versus EWT-indazole-π-A fluorophore in solution-state, we subsequently carried out the two-dimensional 2D-NOESY NMR experiments. In 2D-NOESY NMR spectra of DC-IN-NH_2_, H^a^ shows a similar degree of couplings with H^b^ and H^c^ (Fig. 3b, c), which implies that the spatial distance of H^a^-and-H^b^ is comparable with that of H^a^-and-H^c^. The result denotes a much more twisted conformation of DC-IN-NH_2_ in solution-state than that of crystal-state. Different from DC-IN-NH_2_, H^a^ of DC-IN-Boc shows only coupling with H^b^ rather than H^c^ (Fig. 3e, f), which further validates the planar conformation of EWT-indazole-π-A dyes in the solution-state. Taken together, these single crystals and 2D-NOESY NMR analysis strongly confirm the twisted/planar conformation of D-indazole-π-A/EWT-indazole-π-A. According to this information, we hypothesized that the dark state of D-indazole-π-A dyes is due to the C–C bond rotations between indazole and the π-bridge unit, thereby generating pronounced non-radiative decay losses (Fig. 3g). In contrast, EWT-indazole-π-A dyes with bright emission exhibit rigid molecule conformation, inhibiting non-radiative decay losses.

To verify our hypothesis, we investigated the effect of viscosity on the fluorescence of these chromophores. According to a general rule, when rotation is restricted in high-viscosity environments, the non-radiative deactivation is minimized, which results in a fluorescence enhancement^29–34^. Indeed, the emission of DCM-IN-NH_2_ is partly restored in a highly viscous environment (Fig. 3h), which is typical for molecular rotors. In contrast, DCM-IN-Boc shows almost no fluorescent change with viscosity variation (Fig. 3i). Collectively, the viscosity sensitivity of DCM-IN-NH_2_ and the viscosity insensitivity of DCM-IN-Boc help elucidate the fluorescence umpolung of indazole-based fluorophores: (i) When modified with EDG (electron-donating group, such as amino group), the obtained fluorophores show obvious C–C bond rotations between indazole and the π-bridge, which leads to a fluorescence dark state. (ii) On the other hand, when modified with EWT (such as Boc group), the C-C bond rotation is inhibited and the fluorophores show bright emissions. Taken together, these results strongly confirm the dark-state/emission properties in the rotation/rigid forms of indazole-based chromophores. Therefore, this fluorescence umpolung of these chromophores could be attributed to the effects of the electron density perturbation on intramolecular rotation (Fig. 3g).

**Fig. 4:**
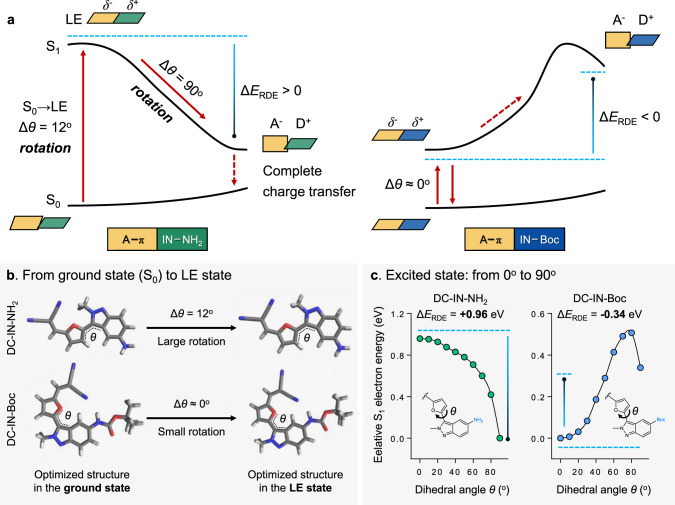
Quantum chemical calculations for elucidating the key role of intramolecular rotation in the fluorescence umpolung mechanism. **a** Jablonski diagram illustrating the distinctly different photoexcitation and deactivation processes of DC-IN-NH_2_ and DC-IN-Boc. **b** Optimized molecular structures of DC-IN-NH_2_ and DC-IN-Boc in the ground state and the locally excited (LE) state. **c** Calculated S_1_ PESs (potential energy surfaces) for DC-IN-NH_2_ and DC-IN-Boc. The theoretical model of describing the LE state (partial charge transfer) → circa 90° twisted excited state (complete charge transfer) photoreaction by plotting S_1_ PES along the rotation angle (*θ*). Source data are provided as a Source Data file. We show that the electron density perturbation of indazole can lead to a mutational effect on intramolecular rotation driving energy (Δ*E*_RDE_), flipping between positive (enhance rotation) and negative (suppress rotation). Δ*E*_RDE_ = *E*(0°) − *E*(90°) (i.e., energy difference between 0° excited state and 90° twisted excited state in the S_1_).

Furthermore, quantum chemical calculations were conducted by using model molecules DC-IN-NH_2_ and DC-IN-Boc (Fig. 4). Their results strongly corroborate the above experimental data, and provide more details in the photoexcitation and deactivation process. Upon photoexcitation, a fluorophore could be excited from the ground state to the locally excited (LE) state, and then experience a transition from the LE state to the circa 90° twisted excited state^35^. As shown in Fig. 4b, we studied the process from the ground state to the LE state. In optimized geometries, DC-IN-NH_2_ exhibits a twisted conformation (*θ* = 12°) in the ground state, whereas demonstrates a typical planar conformation (*θ* = 0°) in the locally excited (LE) state (Fig. 4b). This substantial rotation (Δ*θ* = 12°) could greatly quench the emission of DC-IN-NH_2_. In stark contrast, we noted that DC-IN-Boc displayed a planar conformation both in the ground and excited states (Δ*θ* = 0°), and could thus rationalize the bright emission of DC-IN-Boc (Fig. 4b). These calculation results of the process from the ground state to the LE state can in part explain the fluorescence umpolung phenomena.

Considering the rotations from the ground state to the planar LE state, we further investigated the formations of the circa 90° twisted excited state in DC-IN-NH_2_ and DC-IN-Boc. Calculations of the potential energy surface (PES) in the excited state (S_1_) show that twisted excited state formation is energetically favorable in DC-IN-NH_2_ (rotation driving energy Δ*E*_RDE_ = +0.96 eV) (Fig. 4c). This rotation (Δ*θ* = 90°) is accompanied by a significant reduction in oscillator strength along with enhanced charge separation in DC-IN-NH_2_ (Supplementary Figs. 6–8). In contrast, the twisted excited state is unlikely to occur in DC-IN-Boc due to a large energy barrier and the lack of driving force to enter (Δ*E*_RDE_ = −0.34 eV) (Fig. 4c). We attributed these different excited state tendencies to the varied electron-donating strength of the –NH_2_ group (strong) in DC-IN-NH_2_ in comparison to that of the Boc group (weak) in DC-IN-Boc (Supplementary Figs. 6–8). These results confirm that the electron density perturbation of indazole leads to a mutational effect on the Δ*E*_RDE_, flipping between positive (enhance rotation) and negative (suppress rotation). It is thus concluded that (i) the fluorescence quenching of DC-IN-NH_2_ (D-indazole-π-A type dye) is largely related to the intramolecular rotation in the excited state, and (ii) the fluorescence lighting-up of DC-IN-Boc (EWT-indazole-π-A type dye) is attributed to no rotation in its’ photophysical process (Fig. 4a). In addition to 2-methyl-indazole and 1-methyl-indazole, we extended these findings to other types of insertion building blocks. Specifically, the insertion of other benzo five-membered heterocycle (such as benzothiophene) into ICT scaffold can also lead to the fluorescence umpolung properties (Supplementary Fig. 9). Thus, this molecular strategy is generalizable, yielding a palette of chromophores with fluorescence umpolung that spans visible and near-infrared range (550–700 nm).

### Overturning the ICT probes’ quenching mode into light-up mode for sensing EWTs

The above experiments have demonstrated that indazole-based fluorophores keep silent in the D-indazole-π-A molecules, but emit brightly when substituted with an electron-withdrawing group. We thus hypothesized that this characteristic could be used for light-up (OFF-ON) sensing of EWTs. Upon encountering the corresponding EWT analytes, the transformation from D-indazole-π-A to EWT-indazole-π-A could dramatically light-up the fluorescent signal. In this regard, various OFF-ON fluorescent probes (named Lighter EW Trackers) could be built up via using our specific indazole-based chromophores.

**Fig. 5:**
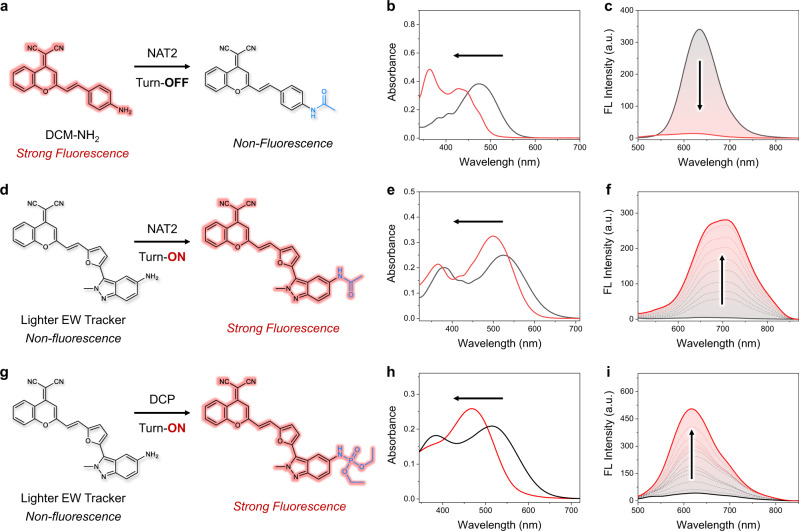
Distinctly different fluorescence response between traditional ICT probe (quenching mode) and indazole-based ICT probe (light-up mode) for sensing EWTs. **a**–**f** The proposed sensing mechanism of DCM-NH_2_ (**a** quenching mode) and Lighter EW Tracker (**d** light-up mode) for NAT2. UV-vis absorption and emission spectra of DCM-NH_2_ (**b**, **c** *λ*_ex_ = 450 nm, 10 μM) and DCM-IN-NH_2_ (**e**, **f** *λ*_ex_ = 500 nm, 10 μM) upon addition of NAT2 mimic (acetyl chloride, from 0 to 2 mM) in acetonitrile solution. **g** Proposed sensing mechanism of DCM-IN-NH_2_ for DCP (light-up mode). UV-vis absorption (**h**) and emission (**i**, *λ*_ex_ = 470 nm) spectra of DCM-IN-NH_2_ (10 μM) upon addition of DCP (from 0 to 3 mM) in acetonitrile solution.

Detection of EWTs, especially for arylamine *N*-acetyltransferases (NATs) and nerve agents, has recently received growing attention in the fields of biological and environmental science. NATs are phase II metabolism enzymes that transfer an acetyl group from acetyl-CoA to aromatic amines and arylhydroxylamines^36^. The detection of NAT2 activity is significant for disease diagnosis and personalized therapy in a clinical setting. Unfortunately, although traditional ICT fluorophores (such as DCM-NH_2_) possess the large Stokes shift, they inevitably exhibit a turn-OFF response toward NAT2 (Fig. 5a–c) owing to the EWT-induced fluorescence quenching. In contrast, our designed indazole-based probes could finely address this limitation (Fig. 5d). As shown in Fig. 5e, upon reaction with NAT2 mimic, Lighter EW Tracker (DCM-IN-NH_2_) with excellent photostability (Supplementary Fig. 10) shows a blue-shift (from 525 to 500 nm) in the absorption spectrum. Simultaneously, a significant fluorescence enhancement (around 56-fold) is observed at 700 nm with a large Stokes shift (200 nm, Fig. 5f). Notably, both the emission wavelength, peak shape (Supplementary Fig. 4c), and high-resolution mass spectrum (HRMS, Supplementary Fig. 11) are coincident with our synthesized DCM-IN-Ac, which further confirms the generation of acylation product upon reaction with the NAT2 mimic. Overall, we have successfully developed an OFF–ON fluorescence probe for light-up sensing of NAT2 based on our fluorescence umpolung strategy.

Encouraged by the application in biosensing, we further explored the environmental monitoring applications of Lighter EW Tracker. Nerve agents (such as sarin, soman, phosgene, and so on) have gained infamy as chemical-warfare agents (CWAs) used in wars in undeveloped countries^37–40^. It’s essential to analyze highly toxic CWAs and related chemicals in a rapid and precise manner. As known, the high toxicity of CWAs is due to their strong capability of nucleophilic attack. Therefore, we reasoned that the active amino group of Lighter EW Tracker could be utilized as the recognition site for light-up sensing of nerve agents.

Then, we studied the spectral response of Lighter EW Tracker toward the nerve-agent mimic diethyl chlorophosphate (DCP). As shown in Fig. 5h, the absorption band at 515 nm becomes gradually decreased and is replaced by a new peak at 470 nm. Simultaneously, the NIR emission becomes enhanced to an intensity that is around 12 times higher than that of the original solution (Fig. 5i). Moreover, the plots of the *I*_650 nm_ against the concentrations of DCP ranging from 0 to 6 μM display a good linear relationship (*r*^2^ = 0.999, Supplementary Fig. 12). Hence, this linear curve allows for the convenient quantitative detection or tracing of DCP over this concentration range. In the HRMS of Lighter EW Tracker with DCP, the peak of DCM-IN-DCP is found at *m*/*z* 568.1739 (Supplementary Fig. 13), which strongly supports that the nucleophilic substitution causes the generation of emissive DCM-IN-DCP (EWT-indazole-π-A type dye). Consequently, Lighter EW Tracker enables the quantitative and light-up detection of nerve agents. More importantly, the fluorescence umpolung-based ICT probes can combine the advantages of light-up sensing and wavelength-reconfigurable mechanisms. That is, Lighter EW Tracker shows a distinctly different light-up with wavelength shift towards different types of EWTs (DCP, phosphorylation: *λ*_em_ = 620 nm, visible region; acetyl chloride, acylation: *λ*_em_ = 700 nm, NIR region; Fig. 5). Clearly, these unique performances cannot be simultaneously achieved with a typical intensity-dependent photoinduced electron transfer (PET) mechanism or the traditional fluorescence-off ICT mechanism. All these results show that the fluorescence umpolung strategy can provide a generalizable method for light-up sensing EWTs including NAT2 and nerve agents.

### Light-up tracking of endogenous NAT2 in living cells and tissue homogenates

**Fig. 6:**
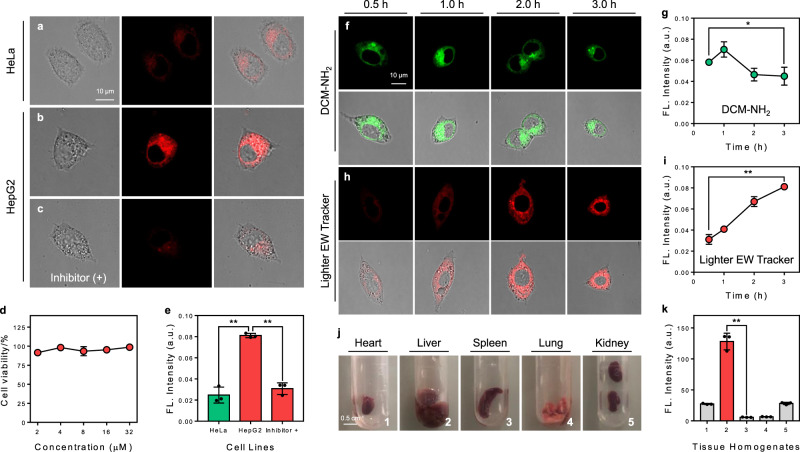
Lighter EW Tracker enables real-time and light-up sensing of endogenous NATs in living cells and tissue homogenates. **a–c** NIR fluorescence imaging (*λ*_ex_ = 488 nm, *λ*_em_ = 700 ± 20 nm) of HeLa cells (**a**) and HepG2 cells incubated with Lighter EW Tracker (DCM-IN-NH_2_, 10 μM, incubated for 2 h) and either untreated (**b**) or treated with (**c**) quercetin (an inhibitor of NATs, 1 mM). Each experiment of **a**–**c** was repeated independently 3 times with similar results. **d** Remarkable biocompatibility of Lighter EW Tracker. Data with error bars are expressed as mean ± s.d., *n* = 3 biologically independent cells. Source data are provided as a Source Data file. **e** Relative NIR fluorescence intensity in cell imaging experiments of **a**–**c**, *p* = 0.0032 (between HeLa and HepG2 group), *p* = 0.0024 (between HepG2 and Inhibitor group). Data with error bars are expressed as mean ± s.d., *n* = 3 biologically independent cells. Source data are provided as a Source Data file. **f**–**i** Time-dependent NIR imaging and relative NIR fluorescence intensity of HepG2 cells incubated with DCM-NH_2_ (**f**, **g** *λ*_ex_ = 488 nm, *λ*_em_ = 650 ± 20 nm) and Lighter EW Tracker (**h**, **i** *λ*_ex_ = 488 nm, *λ*_em_ = 700 ± 20 nm). Each experiment of **f** and **h** was repeated independently 3 times with similar results. Data with error bars are expressed as mean ± s.d., *n* = 3 biologically independent cells, *p* = 0.1579 (in **g**), *p* = 0.0057 (in **i**). Source data are provided as a Source Data file. **j**, **k** NIR fluorescent intensities of tissue homogenates incubated with Lighter EW Tracker (the detailed method is described in the Supplementary Information), *p* = 0.0042. Data with error bars are expressed as mean ± s.d., *n* = 3 biologically independent samples. Source data are provided as a Source Data file. All the *p*-values were performed with one-way ANOVA, *p < 0.5, **p < 0.01.

After investigating the light-up response characteristics of Lighter EW Tracker (DCM-IN-NH_2_) as a NATs (such as NAT2) probe in vitro, we further explored the potential of the probe for live-cell imaging of NATs activation (Fig. 6). Cytotoxicity of Lighter EW Tracker was evaluated by the widely used MTT assay. As shown in Fig. 6d, when incubated with 2, 4, 8, 16, or 32 μM Lighter EW Tracker for 24 h, the cell viabilities are close to 100%, indicating the remarkable biocompatibility of the probe. Then, a fluorescence confocal microscope was used to image HepG2 and HeLa cells after incubation with Lighter EW Tracker. As well known, in humans, NATs are expressed only in specific cells (such as HepG2 cells)^36^. As expected, much stronger NIR fluorescence is detected in HepG2 cells than that of HeLa cells (Fig. 6a, b, e). Indeed, this distinctly different fluorescent intensity (*p* < 0.01) in HepG2 and HeLa cells is consistent with the biodistribution of NATs. Then, co-staining studies confirm that the probes are mainly localized in the cytoplasm, such as the cell mitochondria (Supplementary Fig. 14). Furthermore, the NIR fluorescence in cells with quercetin (inhibitor of NATs) is much weaker (*p* < 0.01) than that of the cells without quercetin (Fig. 6b, c, e). All these results strongly support that Lighter EW Tracker could be used to specifically detect endogenous NATs in cells with a remarkable light-up signal.

More convincing evidence was from the comparison of the time-dependent changes of the fluorescence signal of DCM-NH_2_ (turn-OFF probe) and Lighter EW Tracker (turn-ON probe). As shown in Fig. 6f, g, DCM-NH_2_ shows a bright NIR fluorescent signal after cellular uptake within 0.5 h. Owing to the turn-OFF response toward NATs, the fluorescence of DCM-NH_2_ becomes weaker with time, which inevitably results in misleading information, as the reduction in fluorescence intensities could also be attributed to photobleaching, diffusion, and so on. In contrast, Lighter EW Tracker shows a weak fluorescent signal within 0.5 h, indicating that the probe is non-fluorescent initially with low background interference. As the incubation time elapses, Lighter EW Tracker is activated by endogenous NATs, and the fluorescence intensity increases gradually which reaches a maximum at 1.5 h (Fig. 6h, i). These results clearly demonstrate that Lighter EW Tracker can be used for real-time and light-up monitoring of cellular NATs.

The cell imaging results encouraged us to further detect NATs in different tissue homogenates including heart, liver, spleen, lung, and kidney. Previous findings suggest that human NATs expression is the highest in the liver but is expressed at functional levels in other tissues^41^. As expected, Lighter EW Tracker exhibits the highest fluorescent intensity in livers than other tissues (Fig. 6j, k). Besides, we further conducted (i) selectivity test (Supplementary Fig. 15), (ii) HRMS spectrum of DCM-IN-NH_2_ with homogenates (Supplementary Fig. 16), (iii) in vivo imaging (Supplementary Fig. 17), and (iv) docking calculation (Supplementary Fig. 18). All these results indicated that the light-up fluorescence signals were resulting from *N*-acetyltransferases-catalyzed acylation rather than other possible alternative explanations. All these imaging results further highlight the potential of Lighter EW Tracker for the detection of NATs activity in a clinical setting, which will be very useful for personalized therapy and disease diagnosis.

## Discussion

By employing a simple and generalizable structural modification strategy—insertion of the additional indazole group into ICT fluorophores, we have successfully resolved the long-standing challenge of EWTs-induced fluorescence quenching in ICT fluorophores. This breakthrough is enabled by overturning the intramolecular rotation driving energy (Δ*E*_RDE_), and thus allows the complete reversing of the traditional ICT fluorophores’ quenching mode into the light-up mode.

With the single crystal, 2D-NOESY NMR experiments, and quantum chemical calculations, we confirmed that the electron density perturbation could modulate the Δ*E*_RDE_ with fluorescence umpolung. The insertion of an indazole building block into the D-π-A motif plays a vital role in both the process from the ground state to the LE state, and the process from the LE state to the circa 90° twisted excited state, and thus leading to the reversal Δ*E*_RDE_ between positive (enhance rotation) and negative (suppress rotation). The D-indazole-π-A fluorophores display obvious intramolecular rotations between indazole and π-bridge (Δ*E*_RDE_ > 0), thereby accounting for the dark state. On the other hand, the EWT-indazole-π-A fluorophores show non-rotation (Δ*E*_RDE_ < 0), and exhibit 120-fold emission enhancement. Starting with classic D-π-A chromophore like a typical laser dye DCM, We have expanded this molecular engineering strategy to other D-π-A featured chromophores and other insertion building blocks (benzo five-membered heterocycles) that span the visible and NIR range (550–700 nm), and the corresponding chromophores with fluorescence umpolung demonstrate the generalizability of our platform. This fluorescence umpolung strategy has allowed for the construction of various probes that make a breakthrough to light-up sense/distinguish EWTs. Based on the unique fluorescent probe named as Lighter EW Trackers, we showed the quantitative and light-up detection of nerve agents, and real-time monitoring of endogenous NATs in living cells and tissue homogenates with high fidelity. We anticipate that our strategy of fluorescence umpolung would greatly expand the bio-analytical toolboxes for both basic life science research and clinical applications, and altogether push the limits of biological imaging.

## Methods

### Materials and general methods

Unless specially stated, all solvents and chemicals were purchased from commercial suppliers in analytical grade and used without further purification. The ^1^H and ^13^C NMR spectra were recorded on a Bruker AM 400 spectrometer using TMS as an internal standard. The high-resolution mass spectrometry data were obtained with a Waters LCT Premier XE spectrometer. The single-crystal data were obtained with a Bruker D8 Venture X-Ray Diffractometer. UV-vis absorption spectra were collected on a Varian Cary 500 spectrophotometer, and fluorescence spectrum measurements were performed on a Varian Cary Eclipse fluorescence spectrophotometer. Confocal fluorescence images were taken on a Leica TCS SP8 (×63 oil lens). Synthesis methods for all compounds, characterization data are provided in the Supplementary Information (Supplementary Figs. 1–3 and Supplementary Figs. 19–77).

### Theoretical calculation details

M06-2X/Def2-SVP and CAM-B3LYP/def2SVP calculations were carried out using Gaussian 16 A^42^. Density functional theory (DFT) and time-dependent DFT (TD-DFT) were employed to investigate the fluorescence (quenching) mechanism of all compounds. All structural optimizations in the ground and excited states were performed using M06-2X functional^43^ and Def2-SVP basis set^44^. Solvation effects in dimethyl sulfoxide (DMSO) were taken into account using the SMD model^45^. Frequency calculations were performed to confirm that we obtained stable structures without imaginary vibrational frequencies. The potential energy surface (PES) in the first excited state (S_1_) was calculated at the same level (M06-2X/Def2-SVP) using relaxed scans with the corrected Linear Response (cLR) solvent formalism around the bond connecting the indazole to the furan moieties. Docking calculation was carried out using AutoDock 4.2.6.

### Cell lines

Hep-G2 and HeLa cell lines were purchased from the Institute of Cell Biology (Shanghai, China). All cells were propagated in T-75 flasks cultured at 37 °C under a humidified 5% CO_2_ atmosphere in RPMI-1640 medium or DMEM medium (GIBCO/Invitrogen, Camarillo, CA, USA) which were supplemented with 10% fetal bovine serum (FBS, Biological Industry, Kibbutz Beit Haemek, Israel) and 1% penicillin–streptomycin (10,000U mL^−1^ penicillin and 10 mg mL^−1^ streptomycin, Solarbio life science, Beijing, China).

### Cellular imaging

The cells at 1 × 10^5^ cells/well were seeded onto glass-bottom Petri dishes with a complete medium (1.5 mL) for 12 h. Then the cells were exposed to desired concentrations (10 μM) of fluorophores for 2 h. PBS was used to wash cells three times to clean the background. The cells were rinsed with PBS twice. The images were then photographed by using a confocal laser scanning microscope Leica TCS SP8 (×63 oil lens).

### Animals

We have complied with all relevant ethical regulations for animal testing and research. The procedures for care and use of animals were approved by the East China University of Science and Technology Animal Studies Committee, and all applicable institutional and governmental regulations concerning the ethical use of animals were followed. The 3–4-week-old female BALB/cA nude mice were purchased from Shanghai Genechem Co. Ltd. and maintained under standard conditions. The animals were housed in sterile cages within laminar airflow hoods at 24 °C, 45–65% humidity in a specific pathogen-free room with a 12-h light/12-h dark schedule and fed autoclaved chow and water ad libitum. Production Permit No.: SCXK (Shanghai) 2013-0017. SYXK No. of Shanghai Institute of Materia Medica: SYXK (Shanghai) 2013-0049.

### Reporting summary

Further information on experimental design is available in the Nature Research Reporting Summary linked to this paper.

## Supplementary information

Supplementary information

Reporting summary

## Data Availability

The X-ray crystallographic coordinates for structures reported in this study have been deposited at the Cambridge Crystallographic Data Centre (CCDC), under deposition numbers CCDC-2041874, CCDC-2041875, CCDC-2041872, CCDC-2041871, CCDC-2041873, and CCDC-2041870. These data can be obtained free of charge from The Cambridge Crystallographic Data Centre via www.ccdc.cam.ac.uk/data_request/cif. All other data are available from the corresponding author. Source data are provided with this paper.

## Source data

Source Data file
