# Supplementary material for: Fluorescence umpolung enables light-up sensing of N-acetyltransferases and nerve agents
Source: Nat Commun. 2021 Jun 23;12:3869. doi: 10.1038/s41467-021-24187-5 (PMC8222306; doi:10.1038/s41467-021-24187-5)
Supplement: Supplementary file 1 — Supplementary information [file 41467_2021_24187_MOESM1_ESM.pdf]

Supplementary Information

Fluorescence umpolung enables light-up sensing of N-acetyltransferases and nerve agents

Chenxu Yan[1], Zhiqian Guo[1], Weijie Chi[2], Wei Fu[1], Syed Ali Abbas Abedi[2], Xiaogang Liu[2], He Tian[1] and Wei-Hong Zhu[1]*

*e-mail: whzhu@ecust.edu.cn

Supplementary Figures

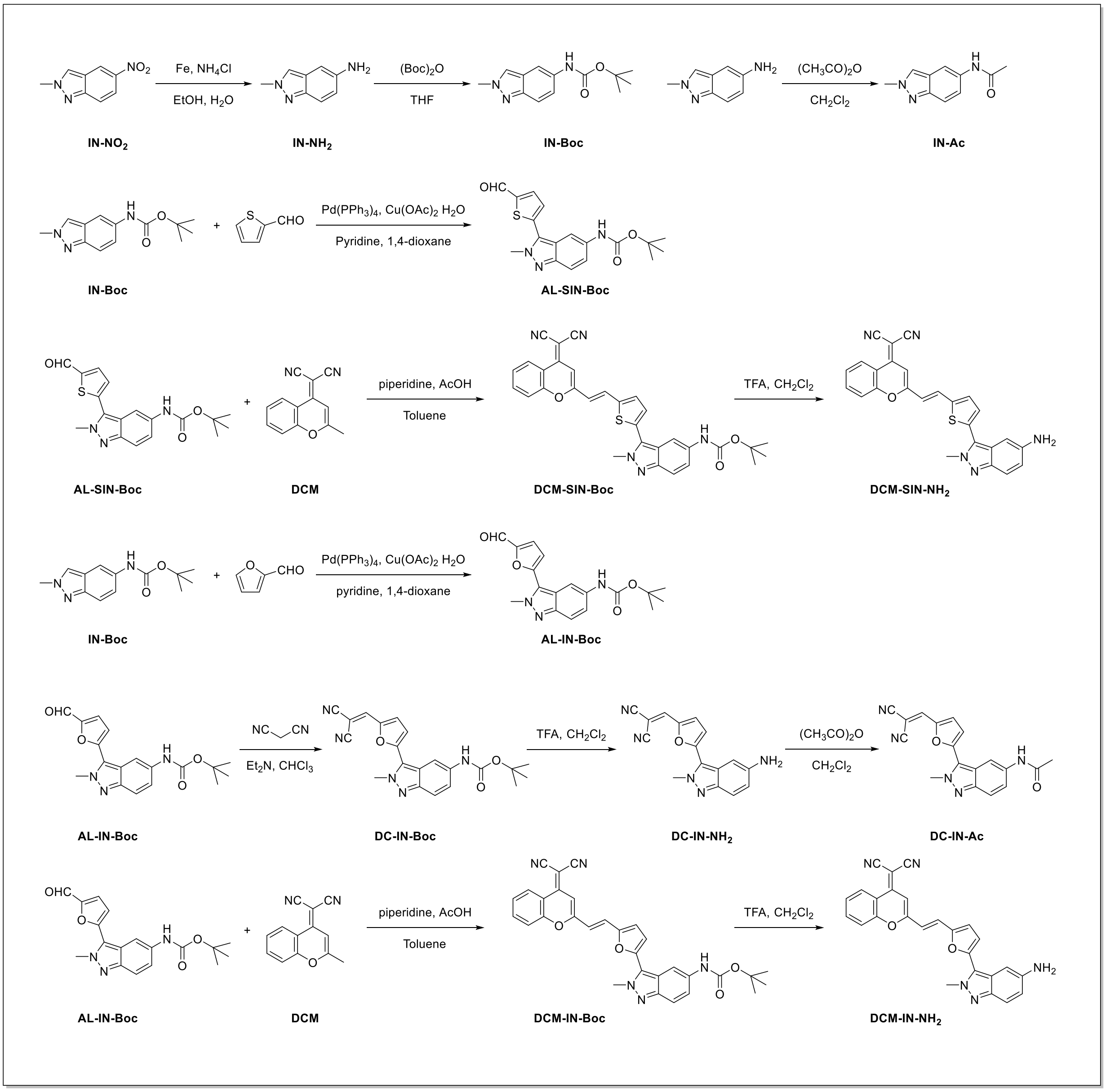

Supplementary Fig. 1. Synthetic route of IN-Ac, DCM-SIN-NH_2, DC-IN-Ac, and DCM-IN-NH_2.

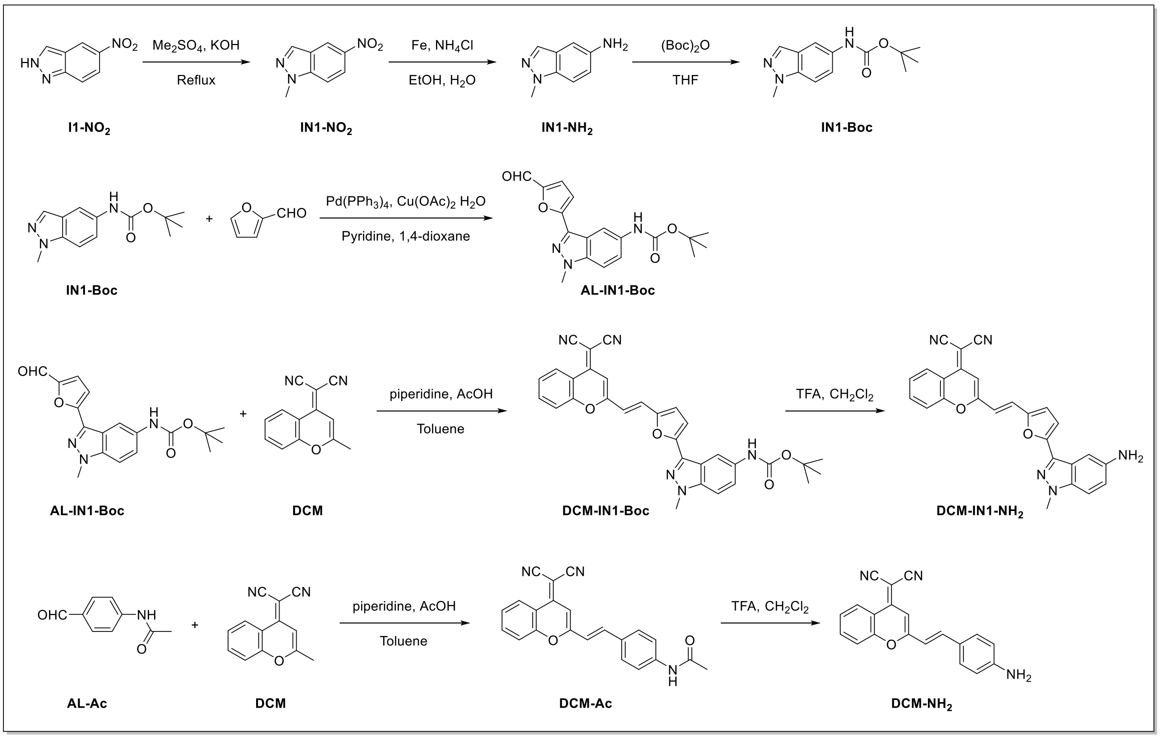

Supplementary Fig. 2. Synthetic route of DCM-IN1-NH_2 and DCM-NH_2

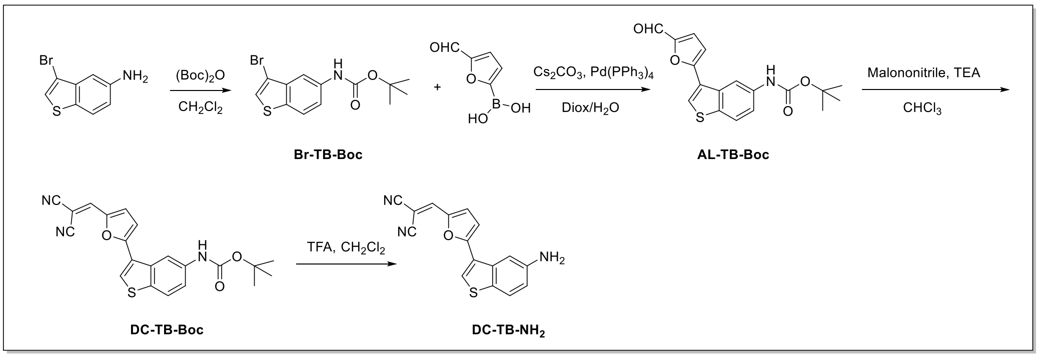

Supplementary Fig. 3. Synthetic route of DC-TB-Boc and DC-TB-NH_2

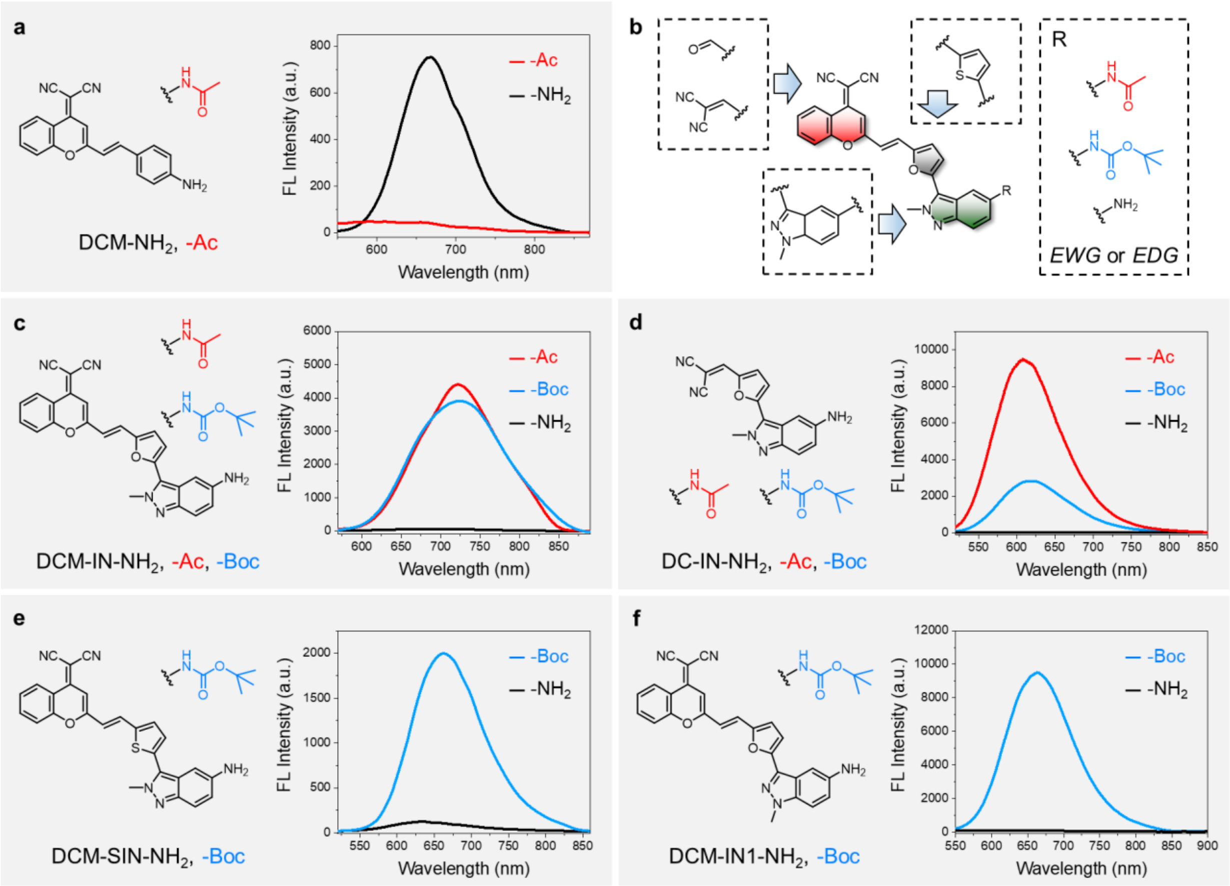

Supplementary Fig. 4. Fluorescence spectra of indazole-based fluorophores. **a**, Fluorescence spectra of DCM-NH_2 (traditional ICT fluorophore, 10 μM) and its electron-withdrawing acetyl substituted product DCM-Ac (10 μM) in DMSO. **b-f**, Design of indazole-based fluorophores: start with D-π-A fluorophore as a basis (π-bridge: thiophene or furan; acceptor: DCM, malononitrile, or aldehyde group); insert an indazole building block (2-methyl-indazole or 1-methyl-indazole) between the π-bridge and the donor. Fluorescence spectra of these indazole-based fluorophores indicated their fluorescence umpolung properties: the D-indazole-π-A type dyes (10 μM, in DMSO) displayed extremely weak emission intensity, whereas their electron-withdrawing Boc substituted products (EWT-indazole-π-A type dyes, 10 μM, in DMSO) exhibited strong fluorescence. DCM-NH_2 and DCM-Ac: λ_{ex} = 500 nm; DCM-IN-NH_2, DCM-IN-Ac and DCM-IN-Boc: λ_{ex} = 510 nm; DC-IN-NH_2, DC-IN-Ac and DC-IN-Boc: λ_{ex} = 500 nm; DCM-SIN-NH_2 and DCM-SIN-Boc: λ_{ex} = 510 nm; DCM-1IN-NH_2 and DCM-1IN-Boc: λ_{ex} = 510 nm.

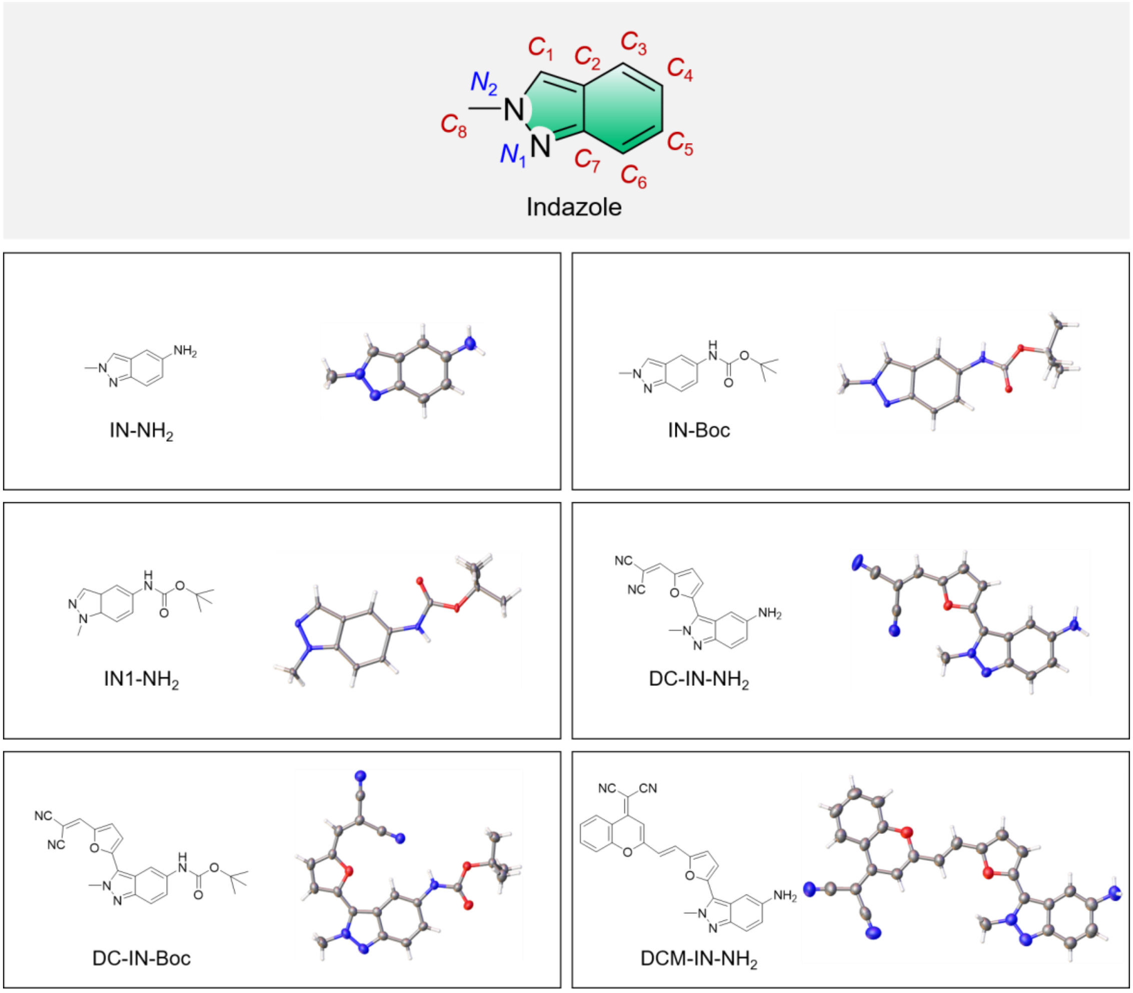

Supplementary Fig. 5. Single-crystal X-ray structures of IN-NH_2, IN-Boc, IN1-NH_2, DC-IN-NH_2, DC-IN-Boc, and DCM-IN-NH_2

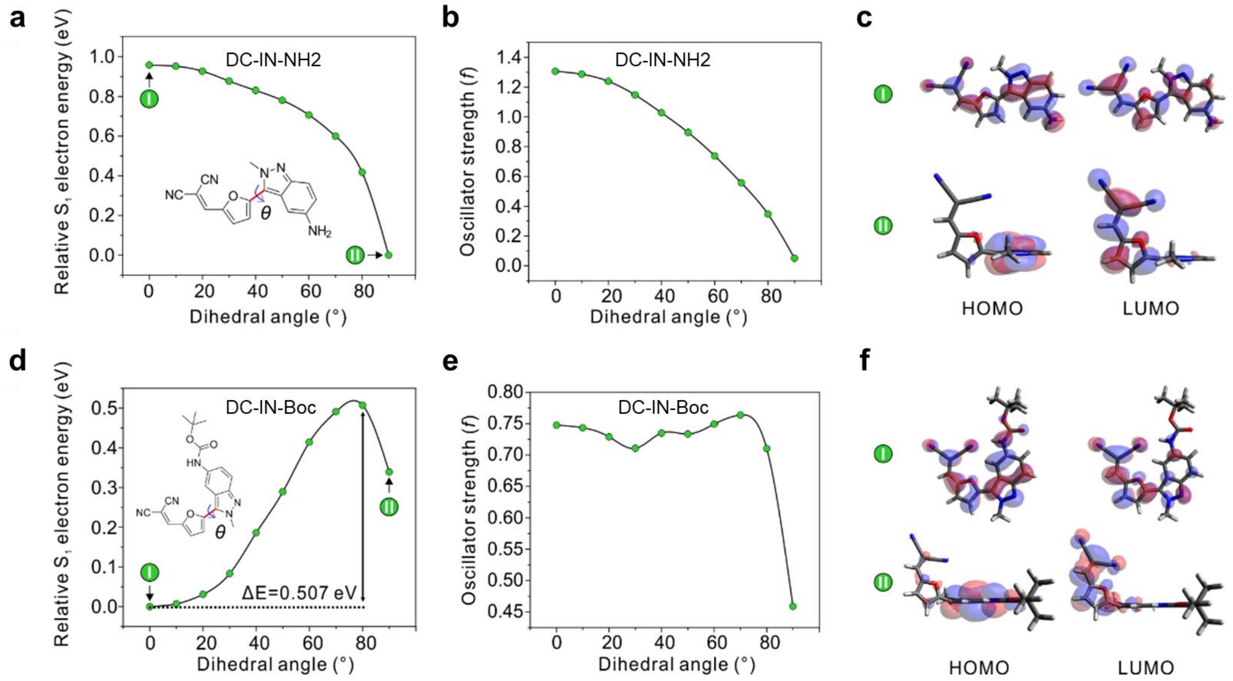

Supplementary Fig. 6. Quantum chemical calculations for elucidating the unusual fluorescence umpolung mechanism. Calculated S_1 potential energy surfaces (**a**), corresponding oscillator strength (**b**) as a function of θ, and representative molecular structures I and II and associated frontier molecular orbitals (**c**) both in the planar and the perpendicular conformations for DC-IN-NH_2 in DMSO. Source data are provided as a Source Data file. Calculated S_1 potential energy surfaces (**d**), corresponding oscillator strength as a function of θ (**e**), and representative molecular structures I and II and associated frontier molecular orbitals (**f**) both in the planar and the perpendicular conformations for DC-IN-NH_2 in DMSO. Source data are provided as a Source Data file.

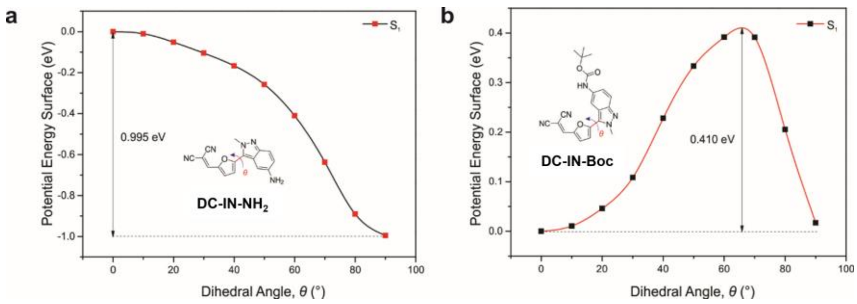

Supplementary Fig. 7. Relative electronic energy of the S_1 potential energy surface (PES) as a function of θ for DC-IN-NH_2 (**a**) and DC-IN-Boc (**b**) in DMSO, calculated using CAM-B3LYP/def2SVP (SMD solvation model with corrected linear response). Source data are provided as a Source Data file.

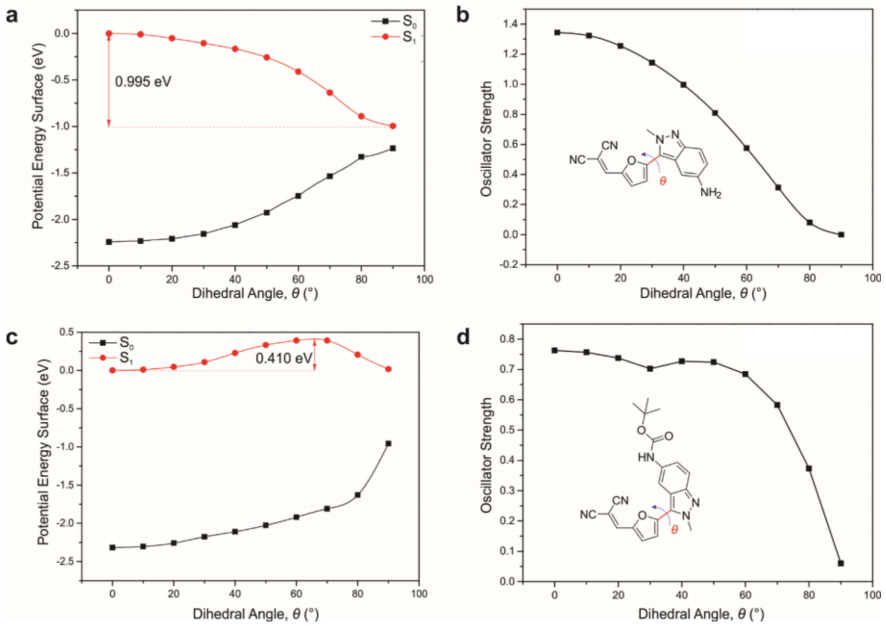

Supplementary Fig. 8. Relative electronic energy of the S_1 potential energy surface (PES) as a function of θ for DC-IN-NH_2 (**a**) and DC-IN-Boc (**c**), and the corresponding oscillator strength during the intramolecular rotations of DC-IN-NH_2 (**b**) and DC-IN-Boc (**d**) in DMSO, calculated using CAM-B3LYP/def2SVP (SMD solvation model with corrected linear response). Source data are provided as a Source Data file. To further confirm the reliability of M06-2X in our study, we have re-performed the excited-state calculations using CAM-B3LYP (a popular DFT functional) in combination with def2SVP basis set. Our results show that both functionals yielded similar results. That is, DC-IN-NH_2 could enter the circa 90° twisted excited state without any energy barrier in the S_1 PES, while DC-IN-Boc faced a significant barrier of 0.410 eV. These electronic structures are in good agreement with the experimental observations of negligible fluorescence in DC-IN-NH_2 and bright emissions from DC-IN-Boc. In summary, these consistencies between functionals and between quantum chemical calculations and experimental observations show that the computational results based on M06-2X are reliable.

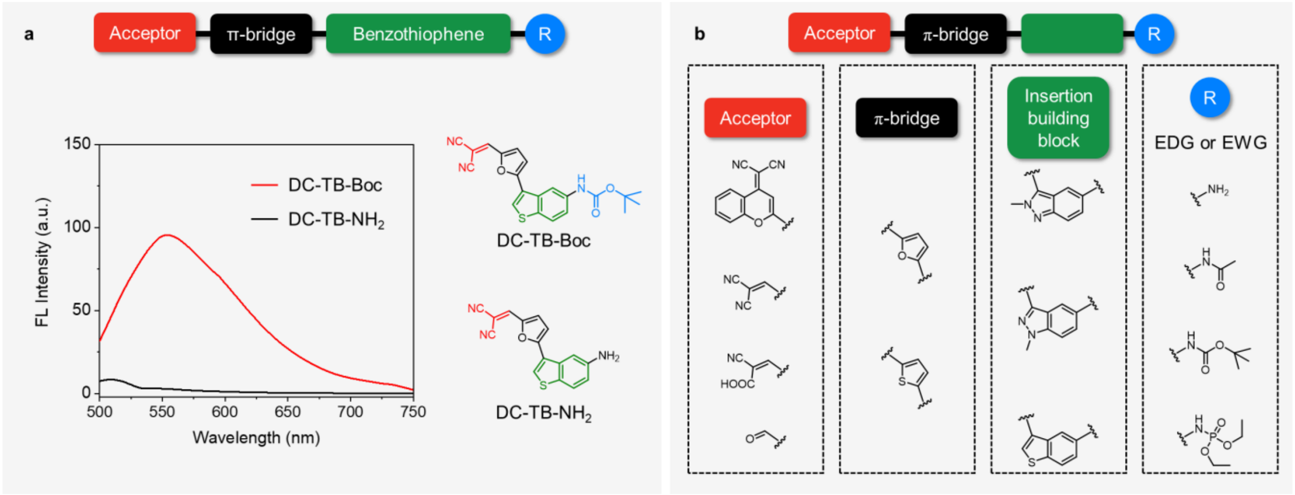

Supplementary Fig. 9. Expanding fluorescence umpolung strategy to other insertion building blocks. **a**, Chemical structure and fluorescence spectra of DC-TB-Boc and DC-TB-NH_2 (10 μm, λ_{ex} = 440 nm) in DMSO. **b**, Engineering the insertion building block for fluorescence umpolung. Insertion of indazole and other benzo five-membered heterocycles (such as benzothiophene) into ICT fluorophores enables fluorescence umpolung.

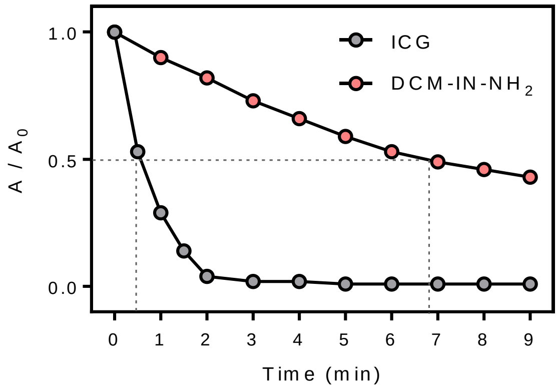

Supplementary Fig. 10. Photostability of DCM-IN-NH_2. Time-dependent absorbance of ICG (10 μM, absorbance at 760 nm) and DCM-IN-NH_2 (10 μM, monitored at 500 nm) under illumination in DMSO/PBS (1/1, v/v, pH 7.4). The time-dependent absorbance measurements were conducted for the photostability of DCM-IN-NH_2 upon continuous illumination (Hamamatsu, LC8 Lightningcure, 300 W), and compared with cyanine dye ICG (the FDA-approved NIR contrast agent). After exposure for about 2 min, the absorbance of ICG decreased sharply to approximate 5% of the initial value, indicative of the almost complete decomposition of the scaffold of ICG, while for DCM-IN-NH_2, more than 80% of the original absorbance at 500 nm survived. Obviously, DCM-IN-NH_2 exhibited much better photostability than ICG. Source data are provided as a Source Data file.

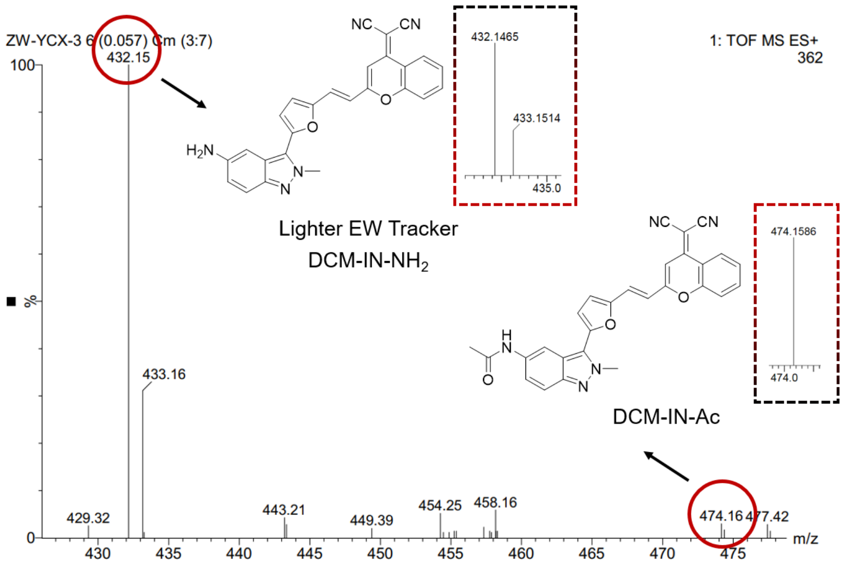

Supplementary Fig. 11. ESI-MS and high-resolution mass spectra (HRMS) of the products from the reaction of Lighter EW Tracker (DCM-IN-NH_2) (10 μM) with NAT2 mimic (acetyl chloride, 1 mM) for 30 min in acetonitrile solution.

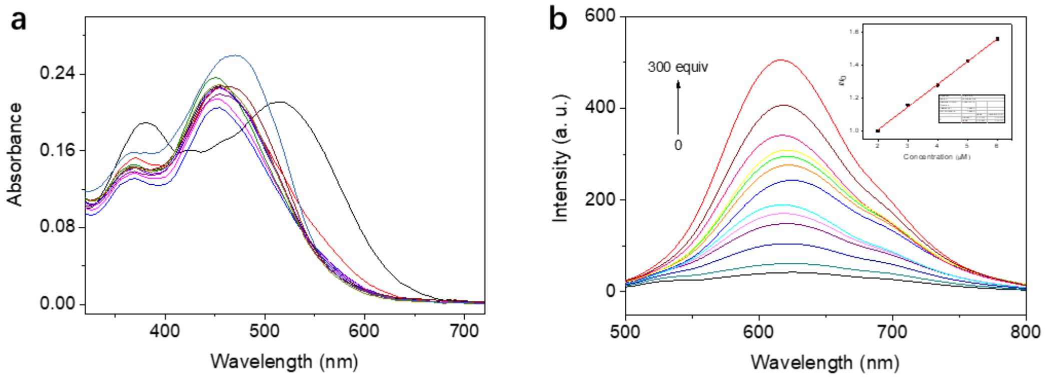

Supplementary Fig. 12. Absorption (**a**) and emission (**b**) spectra spectra of Lighter EW Tracker (DCM-IN-NH_2,10 μM) with increasing concentration of DCP (0-300 equiv) in CH_3CN solution for 10 min, λ_{ex}=470 nm. Inset: Variations in I/I_0 of Lighter EW Tracker with different concentration of DCP, I_0 and I are the emission intensities of DCM-IN-NH_2 (10 μM) at a specific concentration of DCP, respectively. Source data are provided as a Source Data file. Detailed analytical methods: Prepare a 10^{-3} M stock solution of Lighter EW Tracker (MW 431 g mol^{-1}) in DMSO by dissolving 10^{-3} mmol of the probe in 1 mL of DMSO. Prepare 10^{-3} M, 10^{-2} M, and 10^{-1} M stock solutions of the diethyl chlorophosphate (DCP, MW 172 g mol^{-1}) by dissolving 10^{-3} mmol, 10^{-2} mmol, and 10^{-1} mmol of the probe in 1 mL of acetonitrile (CH_3CN), respectively. Add 20 μL of the 10^{-3} M stock solution of Lighter EW Tracker to 2 mL CH_3CN. The final concentrations of Lighter EW Tracker will be 10 μM. This solution will serve as a negative control. Add 20 μL of the 10^{-3} M stock solution of Lighter EW Tracker and 20 μL of the 10^{-3} M stock solution of DCP to 2 mL CH_3CN. The final concentrations will be 10 μM for Lighter EW Tracker and 10 μM for Lighter EW Tracker. This solution will serve as a positive control. After incubation for 10 min, read the fluorescence intensity of both positive and negative control samples. The excitation wavelength is 470 nm.

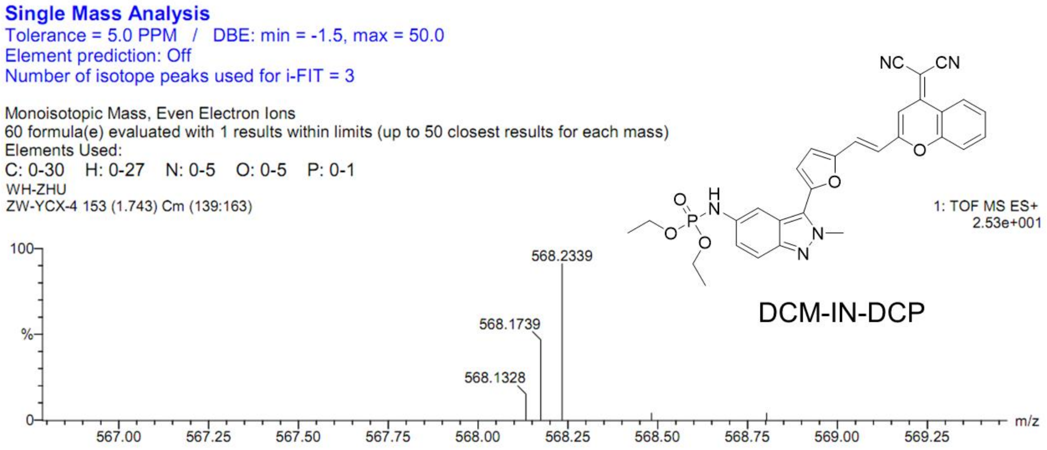

Minimum:				-1.5			
Maximum:		5.0	5.0	50.0			
Mass	Calc. Mass	mDa	PPM	DBE	i-FIT	i-FIT (Norm)	Formula
568.1739	568.1750	-1.1	-1.9	20.5	94.0	0.0	C30 H27 N5 O5 P

Supplementary Fig. 13. HRMS spectrum of DCM-IN-DCP from the reaction of Lighter EW Tracker (DCM-IN-NH_2, 10 μM) with DCP for 30 min in acetonitrile solution.

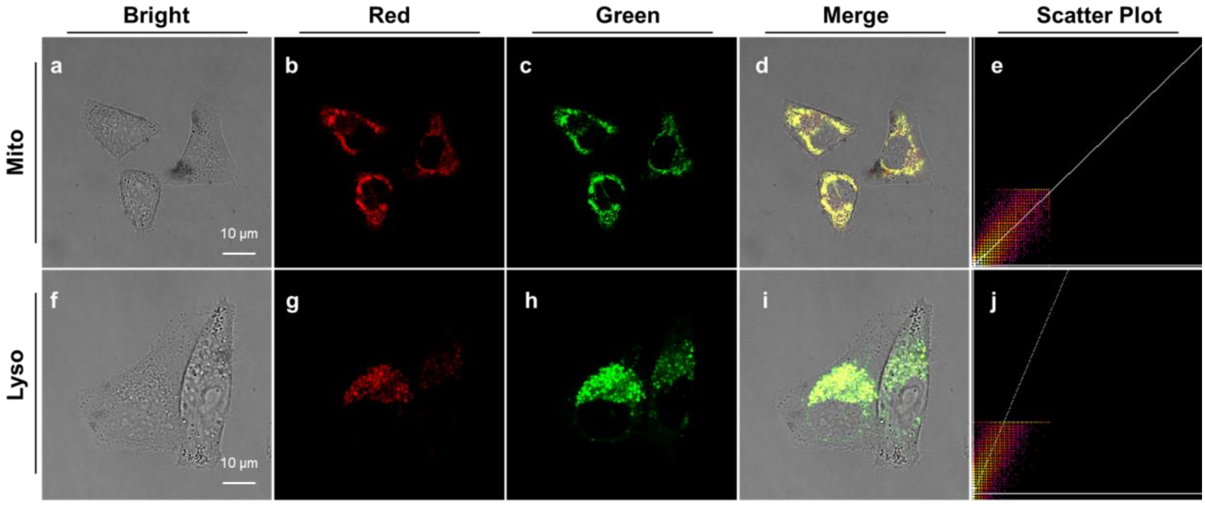

Supplementary Fig. 14. CLSM images for intracellular localization of Lighter EW Tracker (DCM-IN-NH_2) in HepG2 cells. Cells were incubated with Lighter EW Tracker (10 μM) for 3 h and then co-stained with 100 nM Lyso-Tracker Red DND-99 (**a-e**) and 200 nM Mito-Tracker Red (**f-j**) for 30 min, respectively. The green channel at 590-610 nm for Lyso-Tracker Red DND-99 and Mito-Tracker Red, λ_{ex} = 568 nm. The Red channel at 650-800 nm, λ_{ex} = 520 nm. Pearson's correlation coefficient of Lighter EW Tracker is 0.8988 and 0.7822 with Mito-Tracker Red and Lyso-Tracker Red DND-99, respectively. Each experiment was repeated independently for 3 times with similar results.

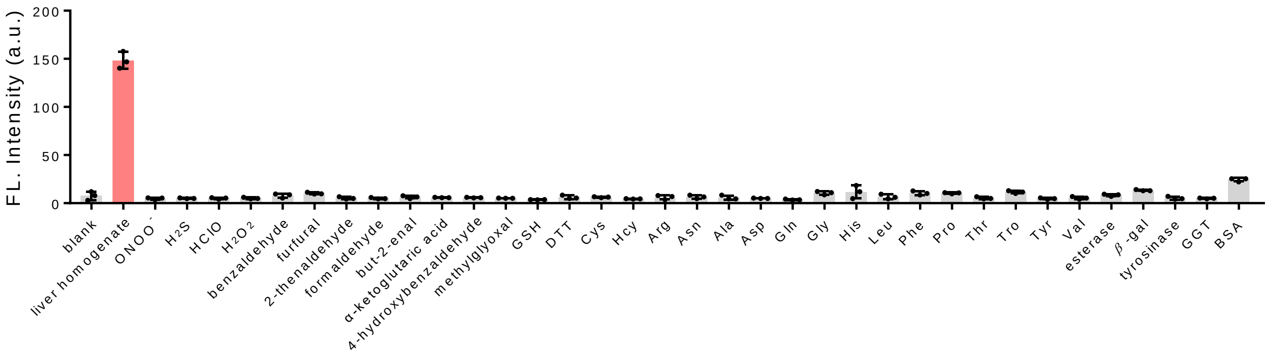

Supplementary Fig. 15. Fluorescence responses (λ_{ex} = 500 nm, λ_{em} = 700 nm) of DCM-IN-NH_2 toward liver homogenate, aldehyde compounds (1 mM), ROS, RNS, amino acids (1 mM), enzymes (1U mL^{-1}), and so on, in PBS/DMSO (9/1, v/v, pH 7.4, 37 °C for 1 hour).β-Galactosidase (β-gal), gamma-glutamyl transpeptidase (GGT), bovine serum albumin (BSA). Data with error bars are expressed as mean ± s.d., n = 3 independent samples. Source data are provided as a Source Data file. In order to exclude these interference factors existing in vivo, we investigated some potential competitive species including amino acid, enzymes, ROS, RNS, aldehyde compound, and so on. As expected, there was subtle change of DCM-IN-NH_2 towards these species. Thus, it could be neglected for DCM-IN-NH_2 with these potential competitive species under the physiological condition.

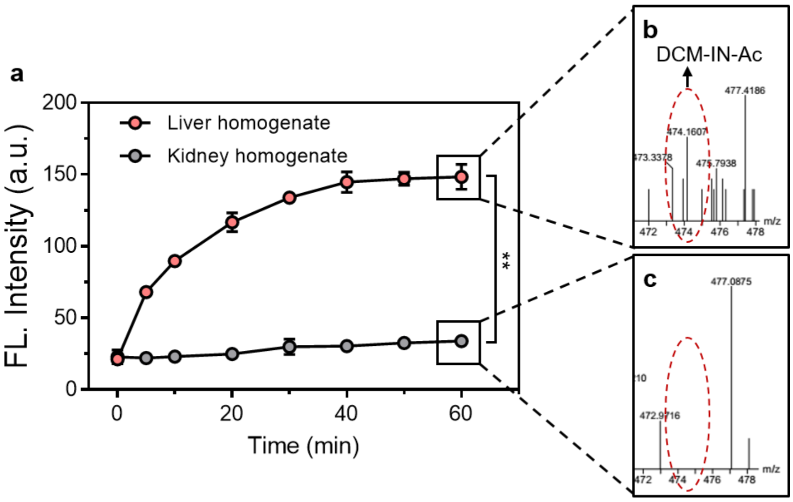

Supplementary Fig. 16. a, Time dependence of fluorescence intensity at 700 nm for Lighter EW Tracker (DCM-IN-NH_2, 10 μM, λ_{ex} = 500 nm) with homogenates (homogenate:PBS:DMSO = 4.5:4.5:1, v:v:v, pH 7.4, 37 °C). Data with error bars are expressed as mean ± s.d., n = 3 biologically independent samples. Statistical significance: p values, $^{**}p < 0.01$, p values were performed with one-way ANOVA, $p = 0.0022$. Source data are provided as a Source Data file. High-resolution mass spectra (HRMS) of the products from the reaction of Lighter EW Tracker (DCM-IN-NH_2) (10 μM) with liver (**b**) or kidney (**c**) homogenate for 1 hour (homogenate:PBS:DMSO = 4.5:4.5:1, v:v:v, pH 7.4, 37 °C). In order to confirm that the light-up fluorescence signals were resulting from NATs-catalyzed acylation, we carried out the HRMS spectrum of DCM-IN-NH_2 with homogenates. Upon treatment with liver homogenate, significant NIR fluorescence was activated. This fluorescence enhancement of DCM-IN-NH_2 in liver homogenate reached a plateau within around 50 min. Subsequently, the anticipated generation of acetylation product was further proven by HRMS analyses. In the presence of liver homogenate, the peak of DCM-IN-Ac at m/z 474.16 (corresponding to $[\text{DCM-IN-Ac} + H]^+$) was observed in the HRMS. Clearly, it was indicated that the acetylation occurred upon exposure to liver homogenate (containing active NATs). In contrast, upon treatment with kidney homogenate (without NATs), DCM-IN-NH_2 showed non-fluorescence response. Accordingly, the peaks of DCM-IN-Ac could not be found in the HRMS. Taken together, the fluorescence light-up response has shown high internal consistency with the generation of acetylation product DCM-IN-Ac. All these pieces of evidence clearly indicated that DCM-IN-NH_2 could be specifically activated by NATs.

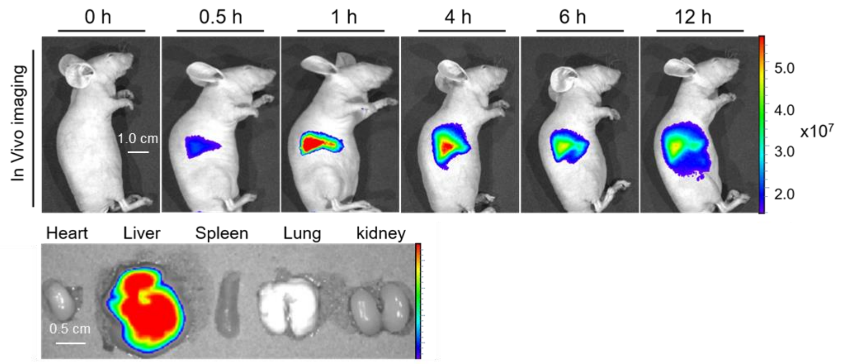

Supplementary Fig. 17. In vivo imaging of DCM-IN-NH_2. In vivo NIR-fluorescence imaging (with a PerkinElmer IVIS Lumina Kinetic Series III imaging system) of nude mice at various time (0, 0.5, 1, 4, 5, and 12 h) after intravenous injection of DCM-IN-NH_2 at a dose of 10 mmol kg^{-1}. Ex vivo NIR-fluorescence imaging of excised organs (tumor, heart, liver, spleen, lung, and kidney) at 12 h after the intravenous injection of DCM-IN-NH_2. The color bars correspond to the detected fluorescence intensity. λ_{ex} = 500 nm, λ_{em} = 700 nm. Each experiment was repeated independently for 3 times with similar results. DCM-IN-NH_2 possessed bright fluorescence intensity in the liver site after 1 h injection, which could be attributed to the endogenous NATs induced acylation of DCM-IN-NH_2. The ex vivo fluorescent images of excised liver further confirmed the activation of DCM-IN-NH_2, with no fluorescence in heart, spleen, lung, and kidney. Indeed, these in vivo and ex vivo fluorescent images were consistent with the results in Fig. 6k. All these imaging results further highlight the potential of DCM-IN-NH_2 for the detection of NATs activity in vitro and in vivo, which will be very useful for disease diagnosis.

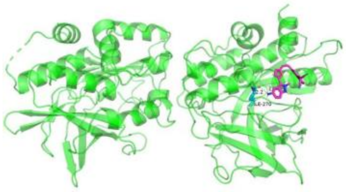

Supplementary Fig. 18. Calculated binding model of DCM-IN-NH_2 to NAT2. To gain a deep understanding of the detection mechanism of probe DCM-IN-NH_2, the NAT2-probe docking calculations are carried out with AutoDock 4.2 docking software in the semi-flexible mode (rigid receptor and flexible ligand). AutoDock has proven to be an effective tool capable of quickly and accurately predicting binding conformations and binding energies between ligands with proteins. Before docking, NAT2 and probe are prepared using the AutoDock Tools 4 software. As known, effective NAT2-catalyzed reaction involves a binding and subsequently a catalysis processes (acylation) process. It should be noted that accurate substrate recognition is a prerequisite for productive enzymatic function. The DCM-IN-NH_2 tends to approach the hydrophobic interspace of NAT2 by the hydrophobic interaction, then a transition state was formed utilizing hydrogen bonding between DCM-IN-NH_2 and NAT2. These results indicate the strong binding ability between NAT2 and DCM-IN-NH_2. Notably, the docking affinities are 8.5 (kcal mol^{-1}) for DCM-IN-NH_2 to NAT2, which is comparable to other high-performance enzyme-specific fluorescence probes[1].

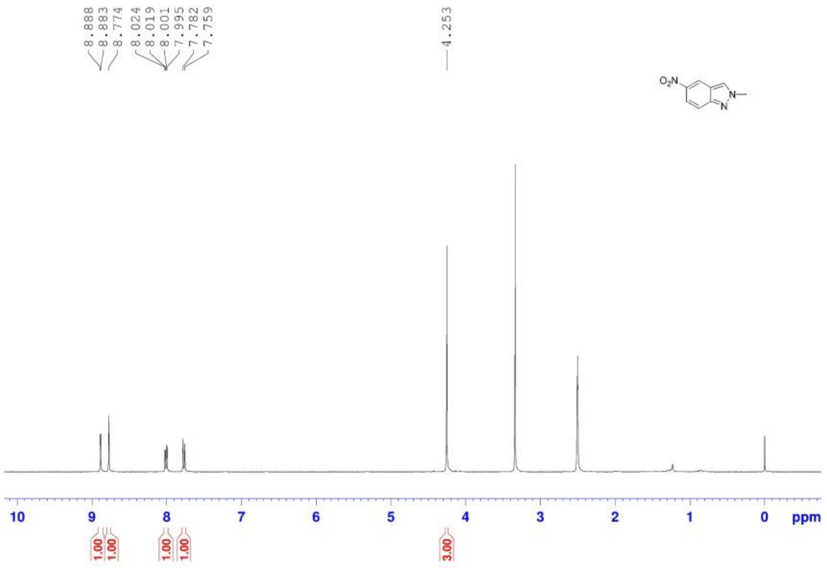

Supplementary Fig. 19. ^{1}H NMR spectrum of IN-NO_2 in DMSO-d_6

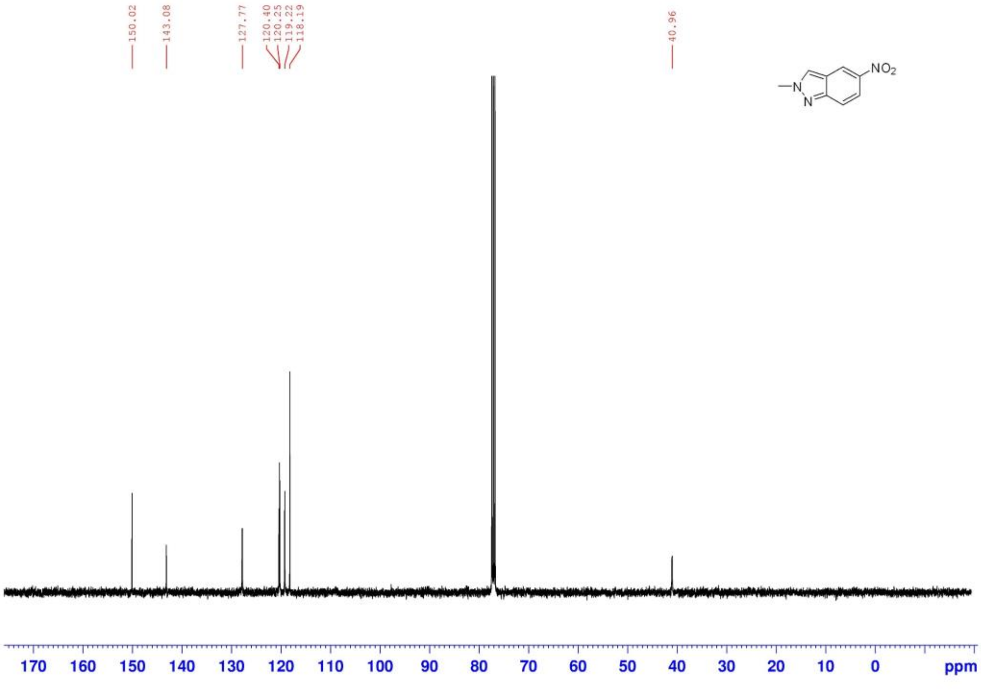

Supplementary Fig. 20. ^{13}C NMR spectrum of IN-NO_2 in $CDCl_3$

Monoisotopic Mass, Even Electron Ions
15 formula(e) evaluated with 1 results within limits (up to 50 best isotopic matches for each mass)
Elements Used:
C: 0-9 H: 0-99 N: 0-3 O: 0-2
WH-ZHU
ZW-YCX-291 75 (0.849) Cm (74:75)

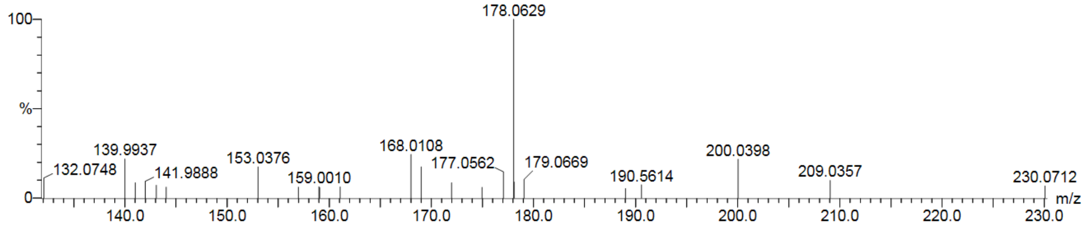

Minimum:				-1.5			
Maximum:		5.0	10.0	50.0			

Mass	Calc. Mass	mDa	PPM	DBE	i-FIT	i-FIT (Norm)	Formula
178.0629	178.0617	1.2	6.7	6.5	30.5	0.0	C8 H8 N3 O2

Supplementary Fig. 21. HRMS spectrum of IN-NO_2

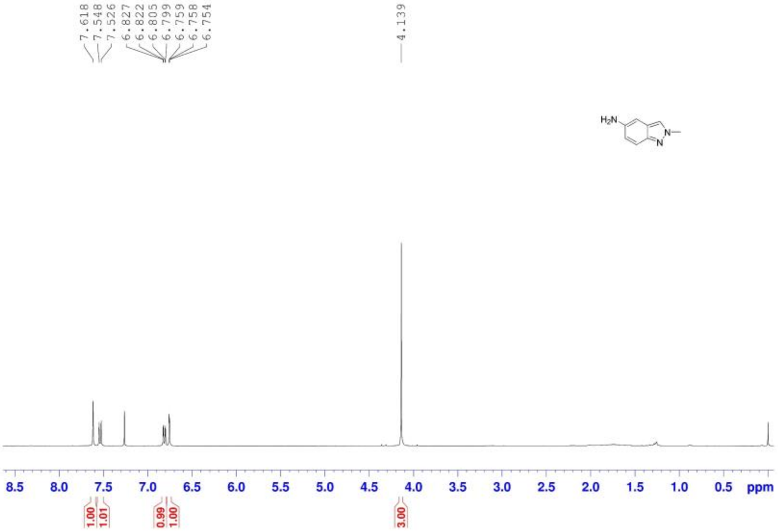

Supplementary Fig. 22. ^{1}H NMR spectrum of IN-NH_2 in $CDCl_3$

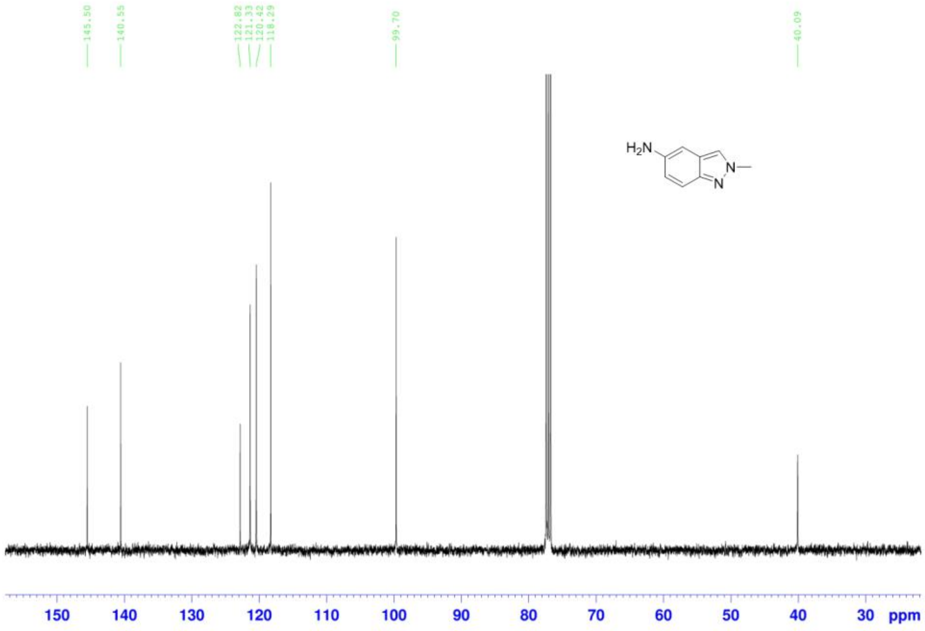

Supplementary Fig. 23. ^{13}C NMR spectrum of IN-NH_2 in $CDCl_3$

Monoisotopic Mass, Even Electron Ions
3 formula(e) evaluated with 1 results within limits (up to 50 best isotopic matches for each mass)
Elements Used:
C: 0-8 H: 0-10 N: 0-3
WH-ZHU
ZW-FW-1 54 (0.610) Cm (50:58)

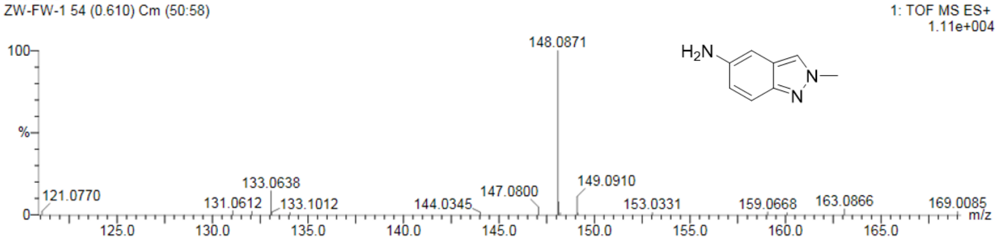

Minimum:				-1.5			
Maximum:		5.0	10.0	50.0			
Mass	Calc. Mass	mDa	PPM	DBE	i-FIT	i-FIT (Norm)	Formula
148.0871	148.0875	-0.4	-2.7	5.5	55.8	0.0	C8 H10 N3

Supplementary Fig. 24. HRMS spectrum of IN-NH_2

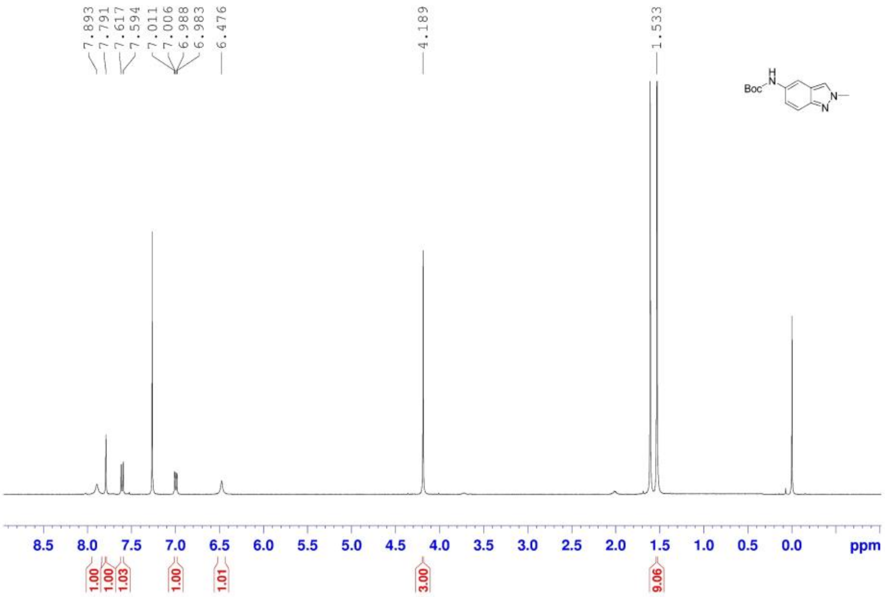

Supplementary Fig. 25. ^{1}H NMR spectrum of IN-Boc in $CDCl_3$

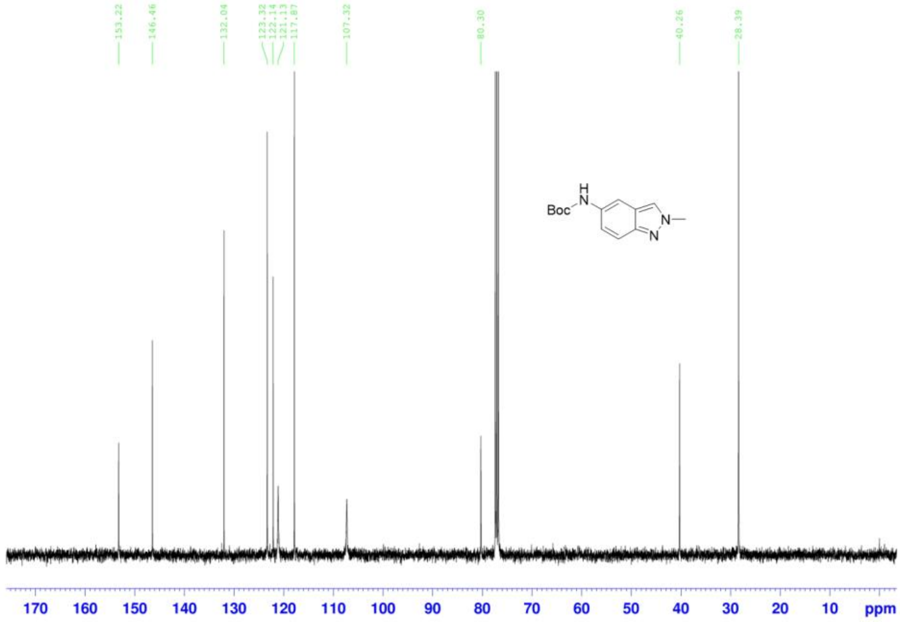

Supplementary Fig. 26. ^{13}C NMR spectrum of IN-Boc in $CDCl_3$

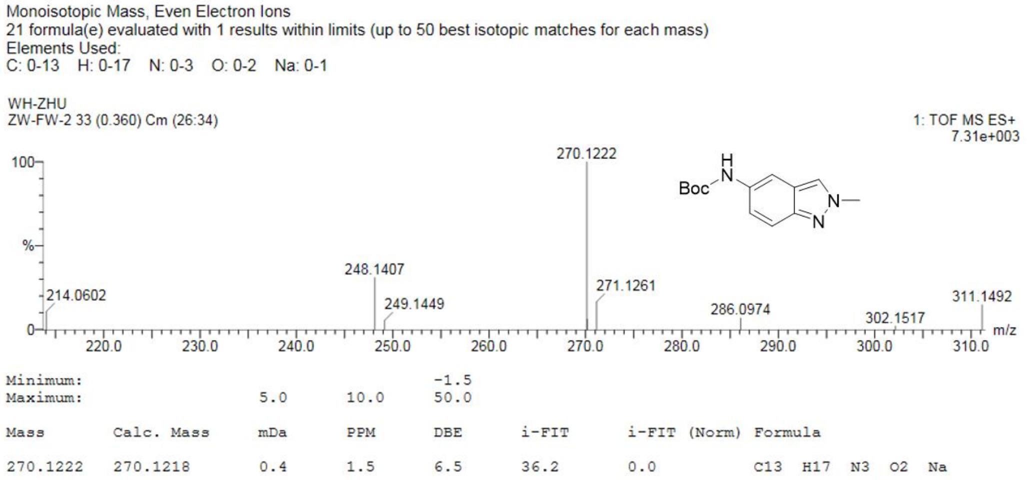

Supplementary Fig. 27. HRMS spectrum of IN-Boc

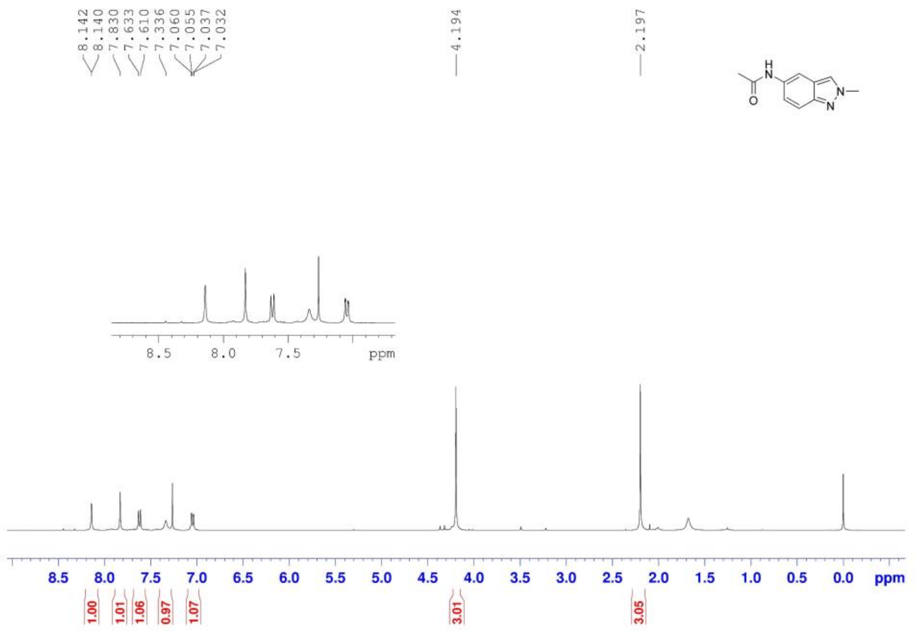

Supplementary Fig. 28. ^{1}H NMR spectrum of IN-Ac in $CDCl_3$

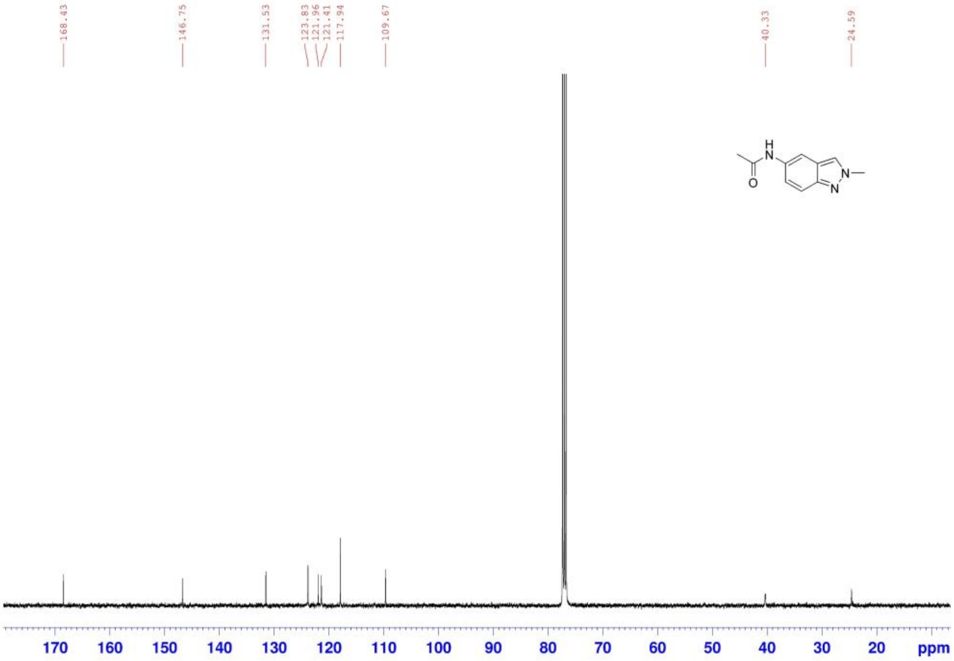

Supplementary Fig. 29. ^{13}C NMR spectrum of IN-Ac in $CDCl_3$

Monoisotopic Mass, Even Electron Ions
6 formula(e) evaluated with 1 results within limits (up to 50 best isotopic matches for each mass)
Elements Used:
C: 0-10 H: 0-12 N: 0-3 O: 0-1
WH-ZHU
ZW-YCX-23 57 (0.636) Cm (54:57)

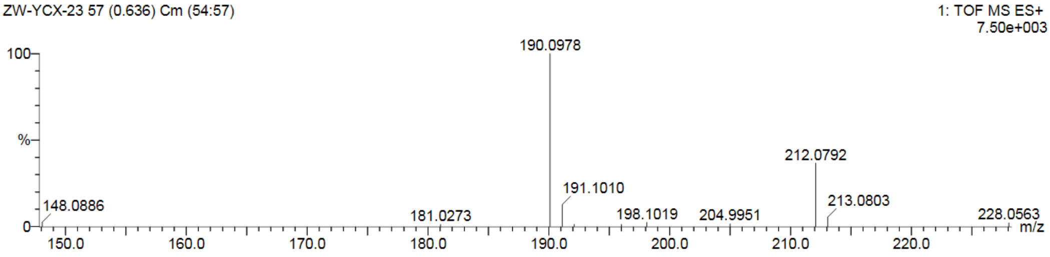

Mass	Calc. Mass	mDa	PPM	DBE	i-FIT	i-FIT (Norm)	Formula
Minimum:				-1.5			
Maximum:		5.0	5.0	50.0			
190.0978	190.0980	-0.2	-1.1	6.5	6.3	0.0	C10 H12 N3 O

Supplementary Fig. 30. HRMS spectrum of IN-Ac

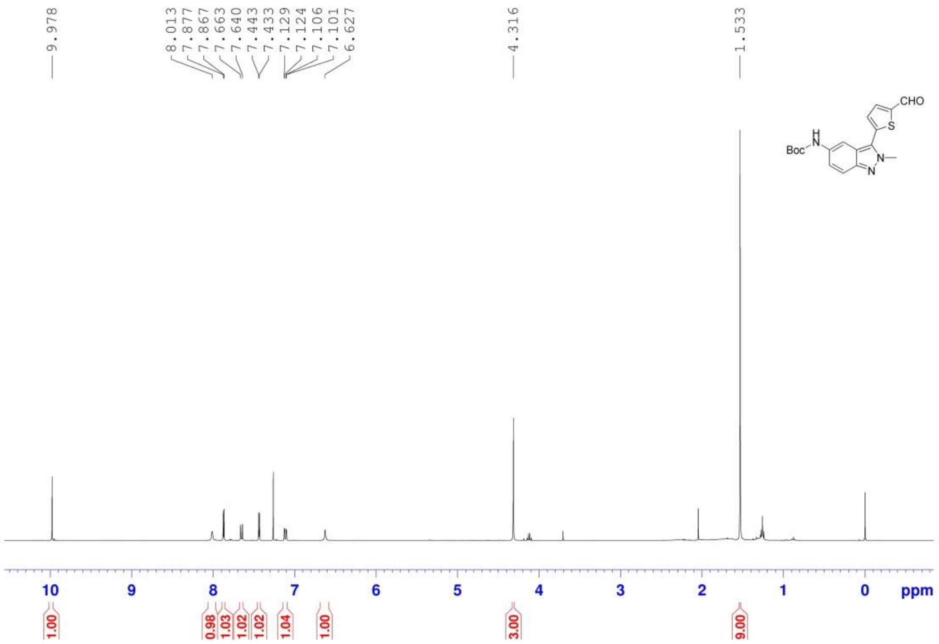

Supplementary Fig. 31. ^{1}H NMR spectrum of AL-SIN-Boc in $CDCl_3$

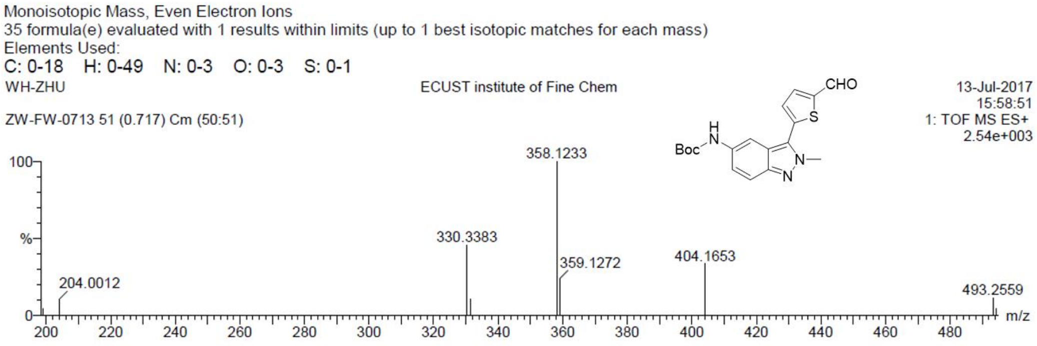

Mass	Calc. Mass	mDa	PPM	DBE	i-FIT	i-FIT (Norm)	Formula
Minimum:				-1.5			
Maximum:		30.0	50.0	100.0			
358.1233	358.1225	0.8	2.2	10.5	28.0	0.0	C18 H20 N3 O3 S

Supplementary Fig. 32. HRMS spectrum of AL-SIN-Boc

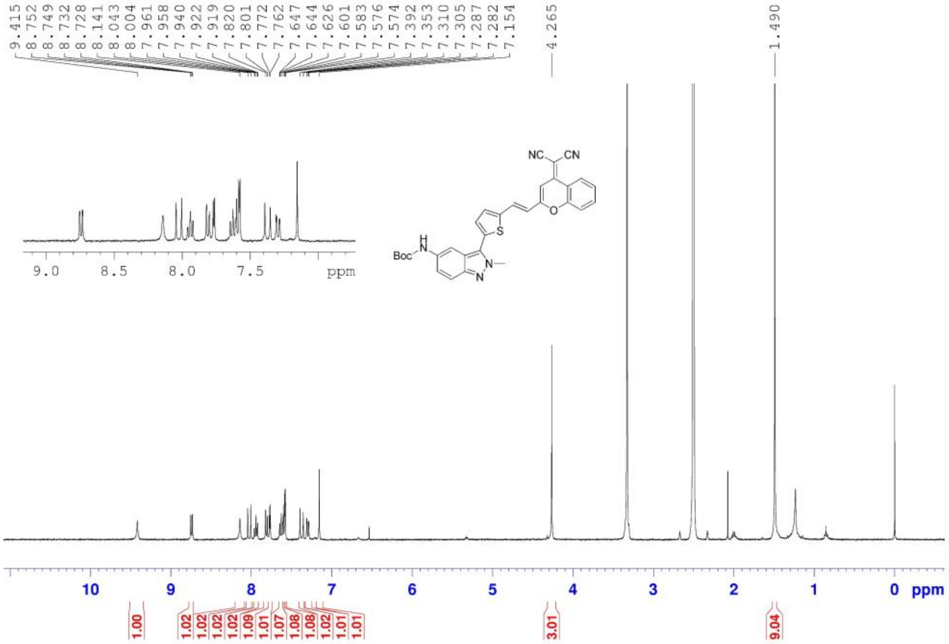

Supplementary Fig. 33. ^{1}H NMR spectrum of DCM-SIN-Boc in DMSO-d_6

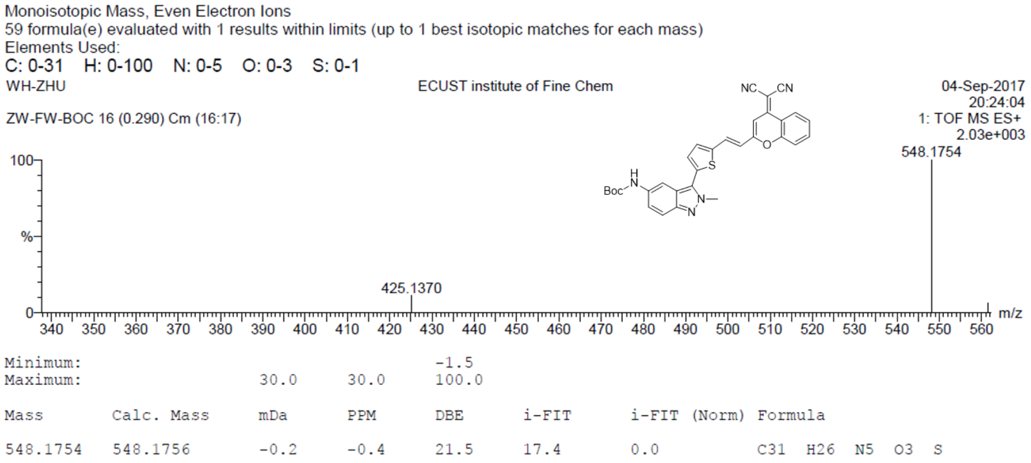

Supplementary Fig. 34. HRMS spectrum of DCM-SIN-Boc

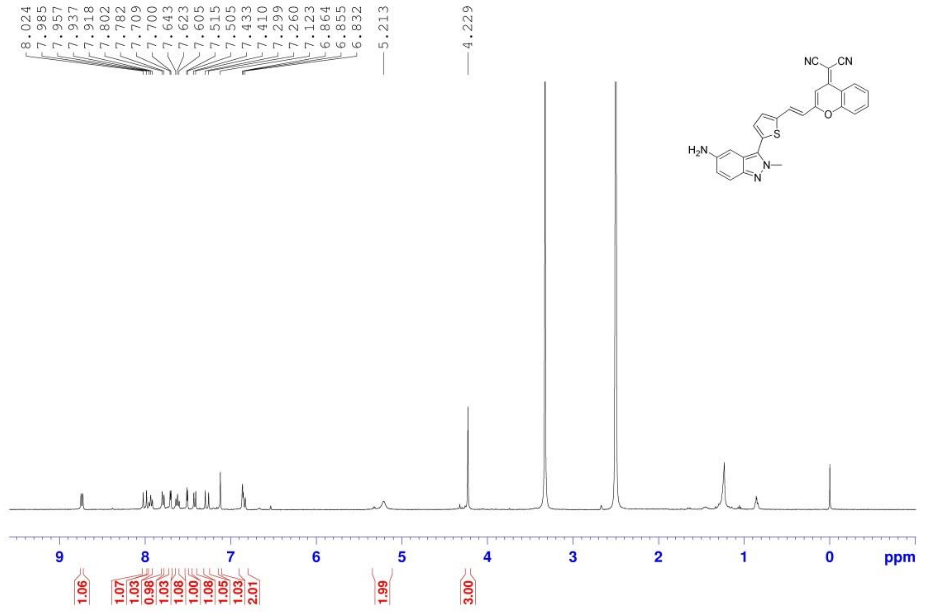

Supplementary Fig. 35. ^{1}H NMR spectrum of DCM-SIN-NH_2 in DMSO-d_6

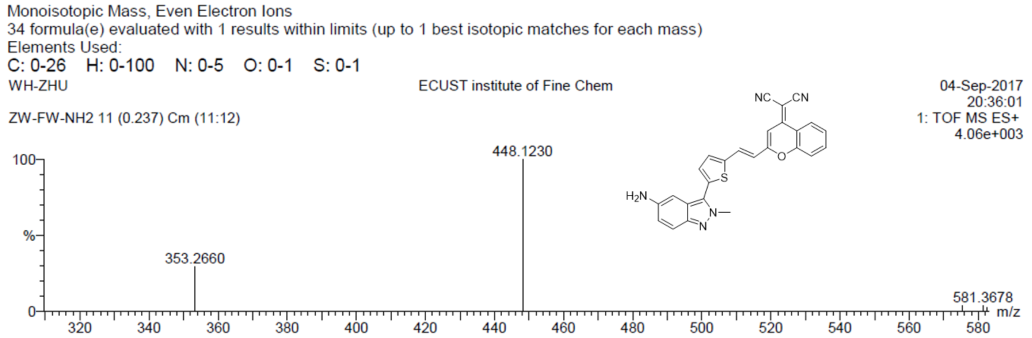

Minimum:				-1.5			
Maximum:		30.0	30.0	100.0			
Mass	Calc. Mass	mDa	PPM	DBE	i-FIT	i-FIT (Norm)	Formula
448.1230	448.1232	-0.2	-0.4	20.5	7.0	0.0	C26 H18 N5 O S

Supplementary Fig. 36. HRMS spectrum of DCM-SIN-NH_2

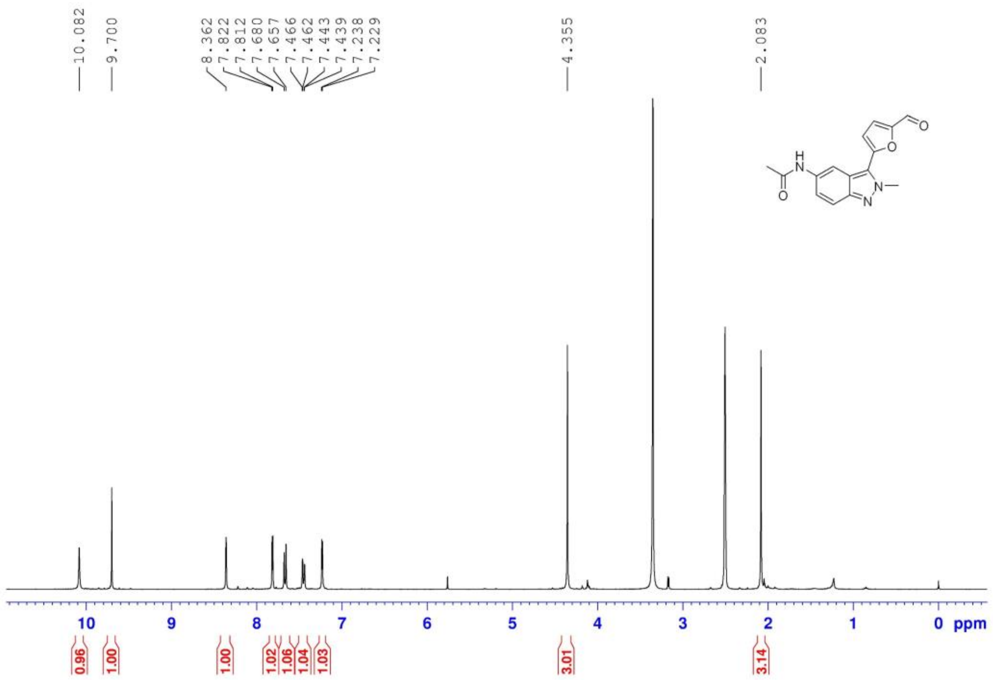

Supplementary Fig. 37. ^{1}H NMR spectrum of AL-IN-Ac in DMSO-d_6

Single Mass Analysis
Tolerance = 5.0 PPM / DBE: min = -1.5, max = 50.0
Element prediction: Off
Number of isotope peaks used for i-FIT = 3

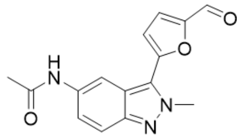

Monoisotopic Mass, Even Electron Ions
12 formula(e) evaluated with 1 results within limits (up to 50 closest results for each mass)
Elements Used:
C: 0-15 H: 0-14 N: 0-3 O: 0-3
WH-ZHU
ZW-YCX-1 26 (0.287) Cm (26:29)

1: TOF MS ES+
2.62e+003

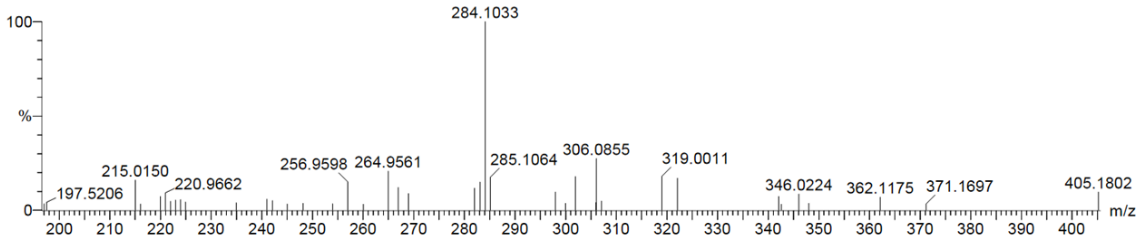

Minimum:				-1.5			
Maximum:		5.0	5.0	50.0			
Mass	Calc. Mass	mDa	PPM	DBE	i-FIT	i-FIT (Norm)	Formula
284.1033	284.1035	-0.2	-0.7	10.5	20.1	0.0	C15 H14 N3 O3

Supplementary Fig. 38. HRMS spectrum of AL-IN-Ac

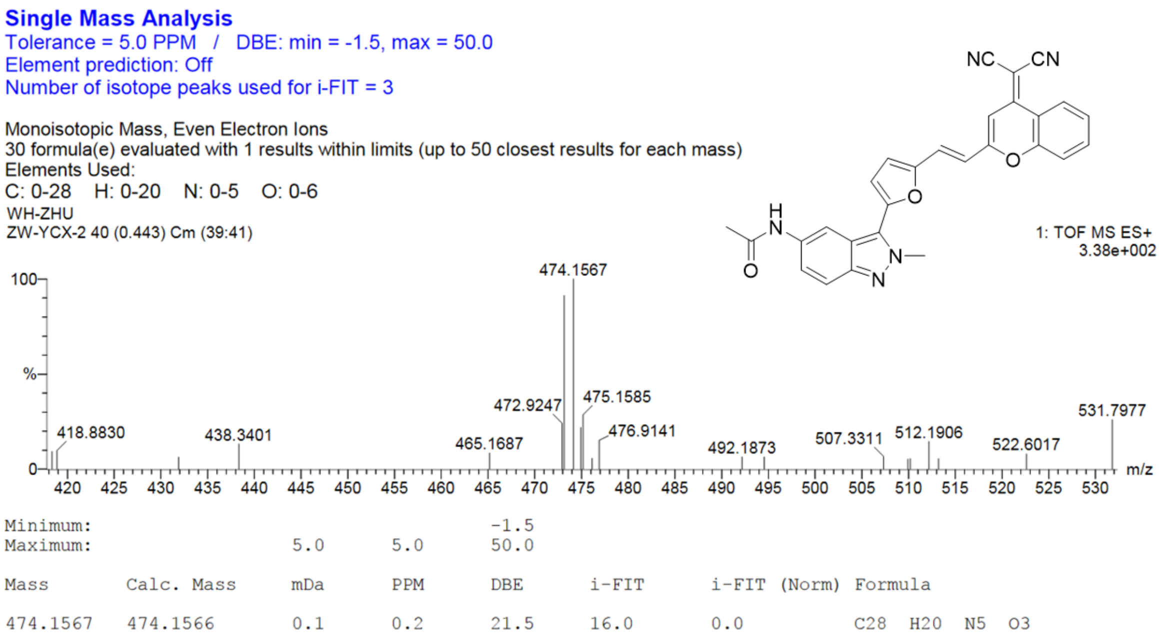

Supplementary Fig. 39. HRMS spectrum of DCM-IN-Ac

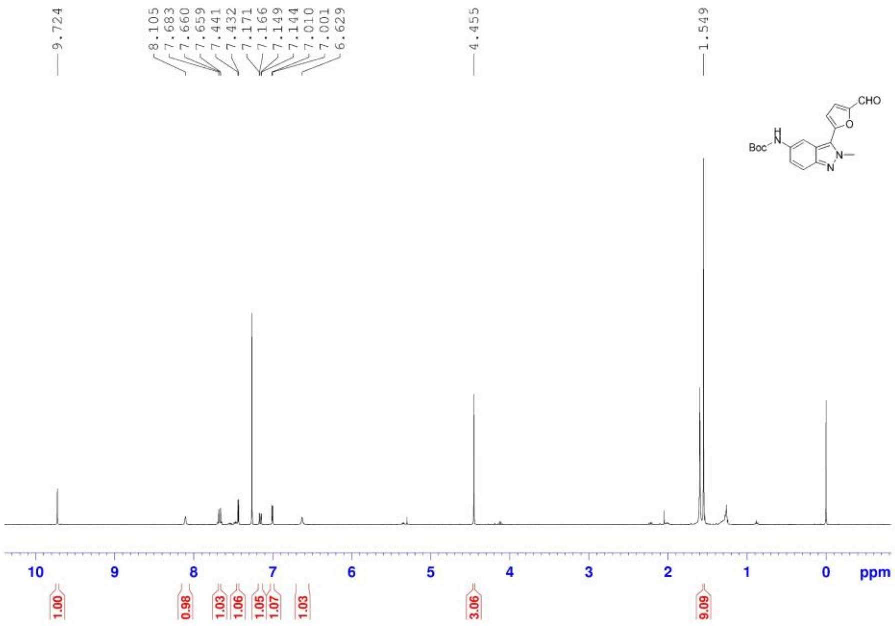

Supplementary Fig. 40. ^{1}H NMR spectrum of AL-IN-Boc in $CDCl_3$

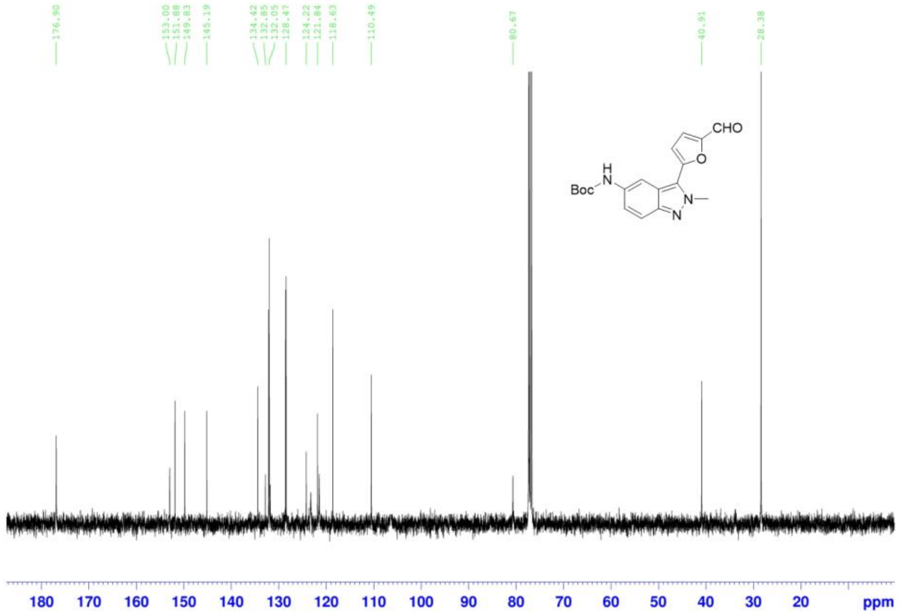

Supplementary Fig. 41. ^{13}C NMR spectrum of AL-IN-Boc in $CDCl_3$

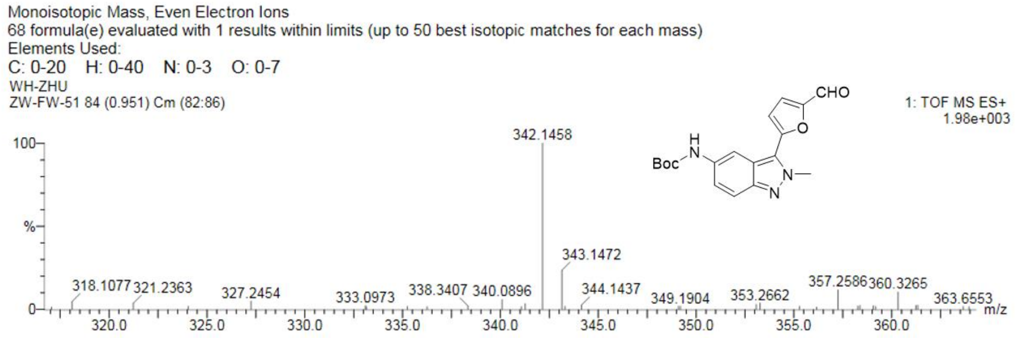

Minimum:				-1.5			
Maximum:		5.0	5.0	50.0			
Mass	Calc. Mass	mDa	PPM	DBE	i-FIT	i-FIT (Norm)	Formula
342.1458	342.1454	0.4	1.2	10.5	17.0	0.0	C18 H20 N3 O4

Supplementary Fig. 42. HRMS spectrum of AL-IN-Boc

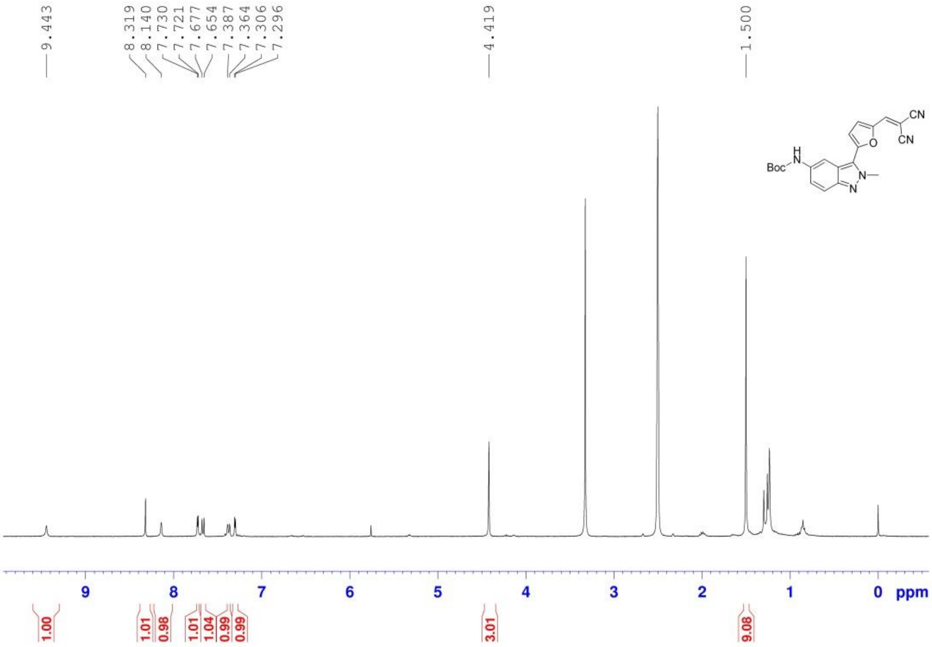

Supplementary Fig. 43. ^{1}H NMR spectrum of compound DC-IN-Boc in DMSO-d_6

Monoisotopic Mass, Even Electron Ions
93 formula(e) evaluated with 2 results within limits (up to 1 best isotopic matches for each mass)
Elements Used:
C: 0-21 H: 0-40 10B: 0-1 11B: 0-1 N: 0-5 O: 0-3

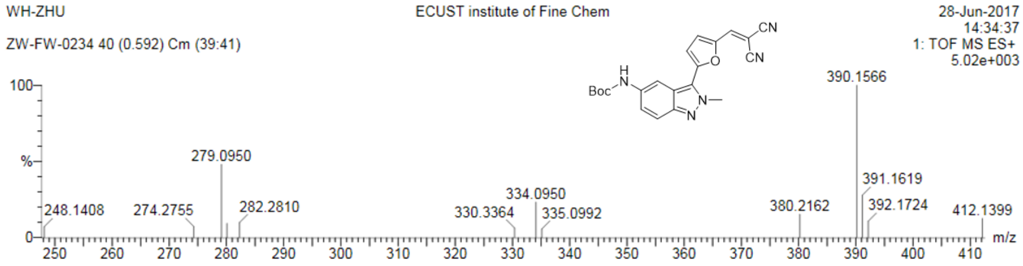

Minimum:				-1.5			
Maximum:		30.0	50.0	100.0			
Mass	Calc. Mass	mDa	PPM	DBE	i-FIT	i-FIT (Norm)	Formula
390.1566	390.1566	0.0	0.0	14.5	21.8	0.0	C21 H20 N5 O3

Supplementary Fig. 44. ^{13}C NMR spectrum of compound DC-IN-Boc in $CDCl_3$

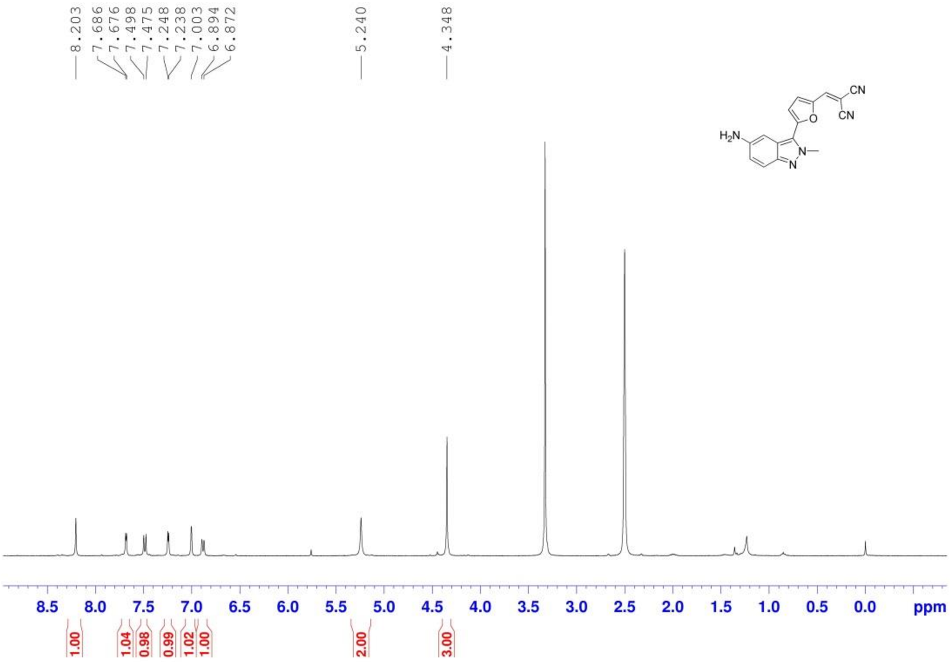

Supplementary Fig. 45. ¹H NMR spectrum of DC-IN-NH_2 in DMSO-d_6

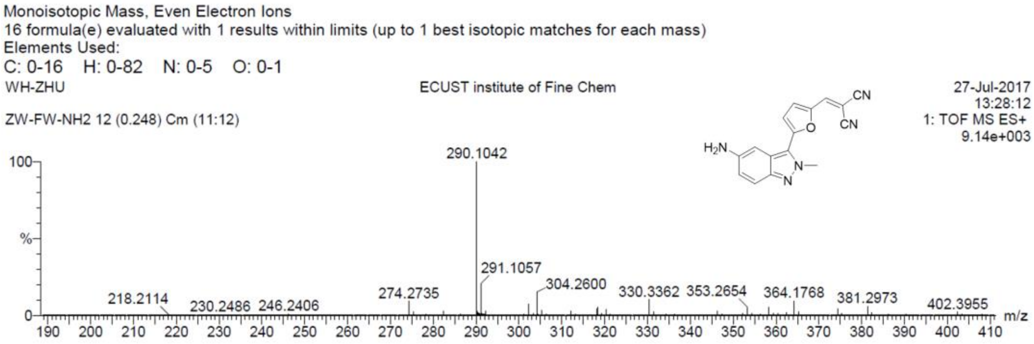

Minimum:				-1.5			
Maximum:		30.0	30.0	100.0			
Mass	Calc. Mass	mDa	PPM	DBE	i-FIT	i-FIT (Norm)	Formula
290.1042	290.1042	0.0	0.0	13.5	314.1	0.0	C16 H12 N5 O

Supplementary Fig. 46. HRMS spectrum of DC-IN-NH_2

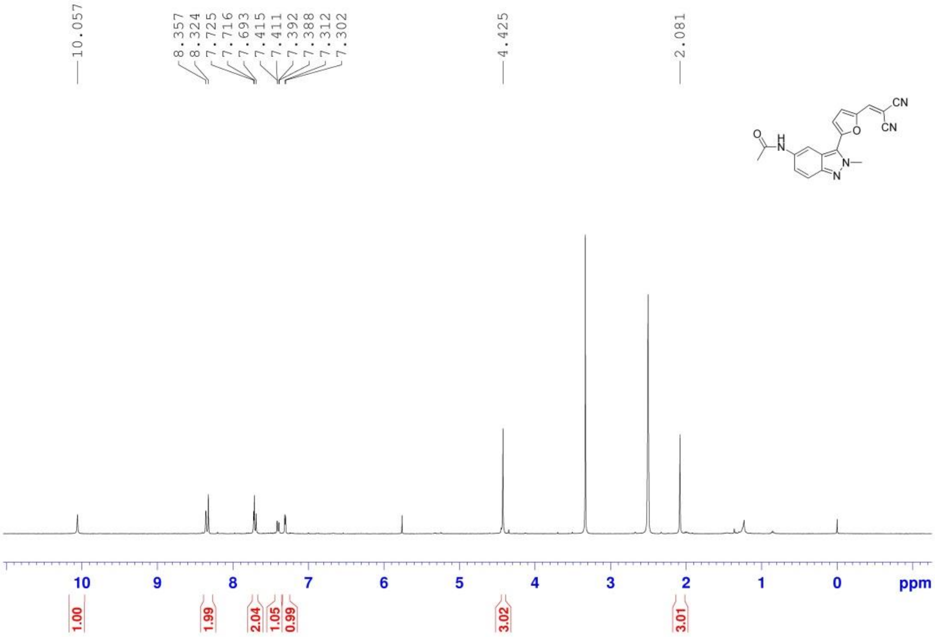

Supplementary Fig. 47. ^{1}H NMR spectrum of DC-IN-Ac in DMSO-d_6

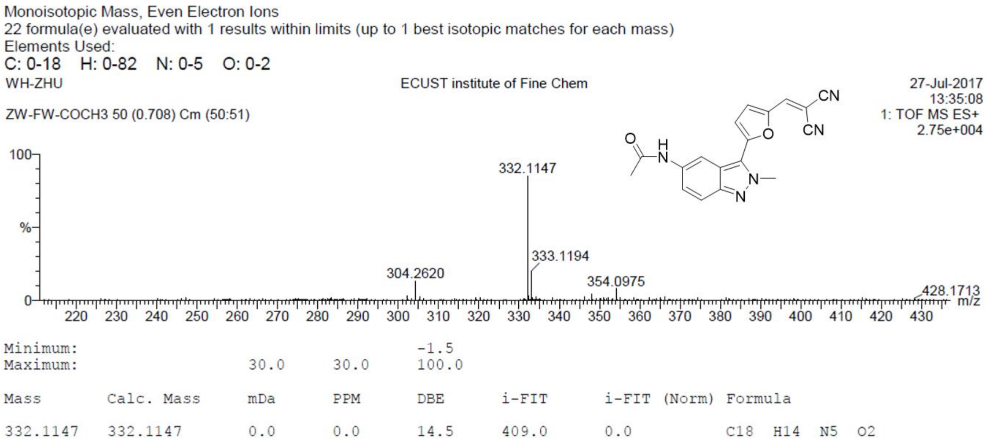

Supplementary Fig. 48. HRMS spectrum of DC-IN-Ac

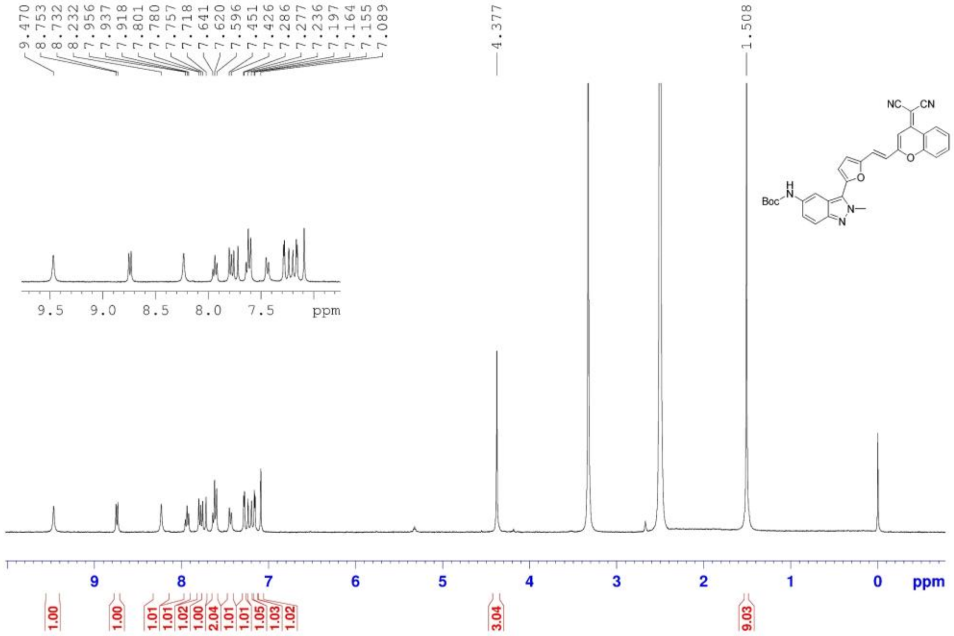

Supplementary Fig. 49. ^{1}H NMR spectrum of DCM-IN-Boc in DMSO-d_6

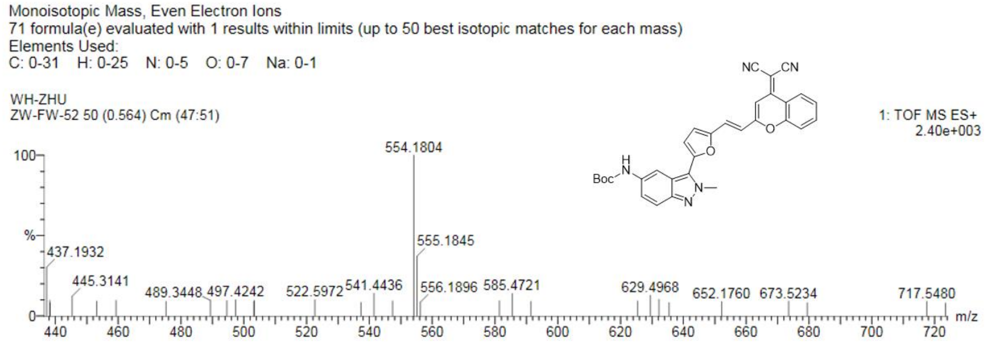

Minimum:				-1.5			
Maximum:		5.0	5.0	50.0			
Mass	Calc. Mass	mDa	PPM	DBE	i-FIT	i-FIT (Norm)	Formula
554.1804	554.1804	0.0	0.0	21.5	8.1	0.0	C31 H25 N5 O4 Na

Supplementary Fig. 50. HRMS spectrum of DCM-IN-Boc

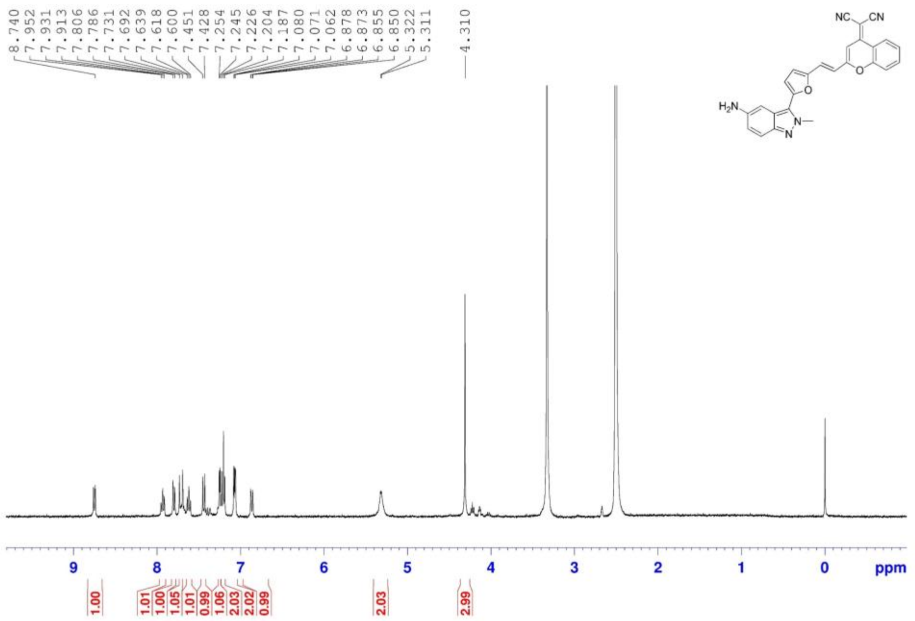

Supplementary Fig. 51. ^{1}H NMR spectrum of DCM-IN-NH$_2$ in DMSO-d_6

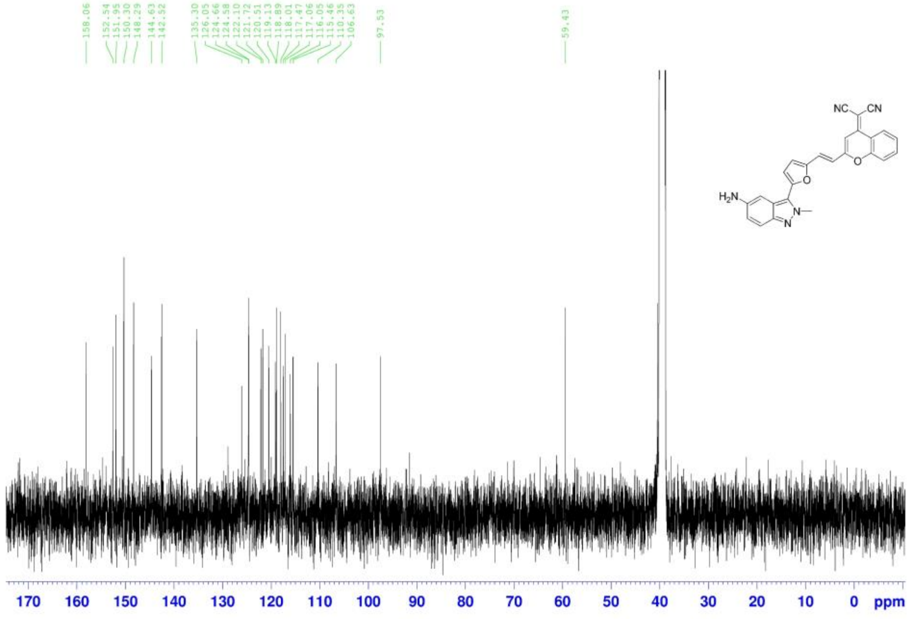

Supplementary Fig. 52. ^{13}C NMR spectrum of DCM-IN-NH$_2$ in DMSO-d_6

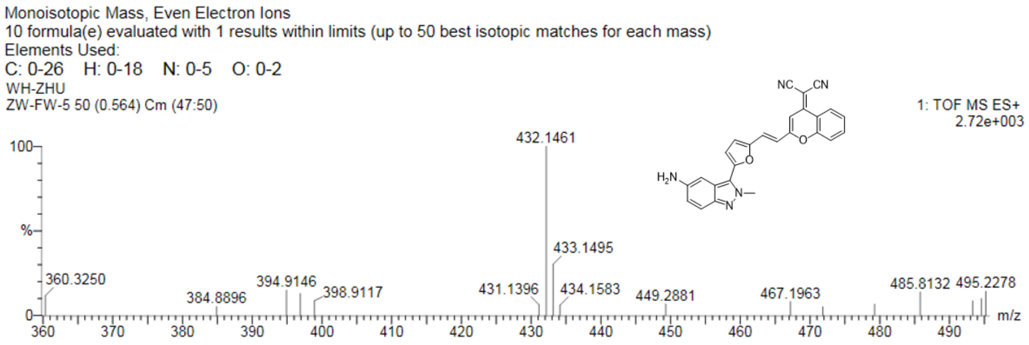

Minimum:				-1.5			
Maximum:		5.0	5.0	50.0			
Mass	Calc. Mass	mDa	PPM	DBE	i-FIT	i-FIT (Norm)	Formula
432.1461	432.1461	0.0	0.0	20.5	10.4	0.0	C26 H18 N5 O2

Supplementary Fig. 53. HRMS spectrum of DCM-IN-NH_2

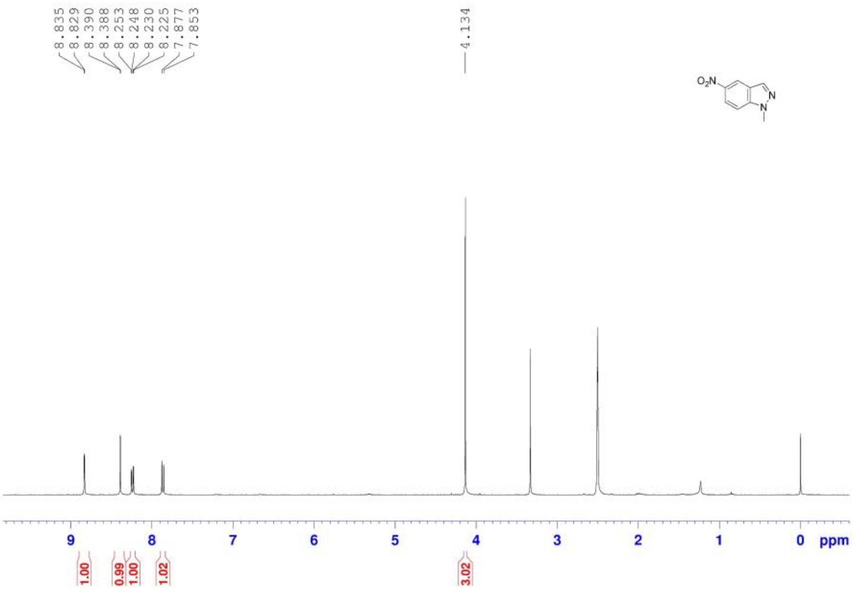

Supplementary Fig. 54. ^{1}H NMR spectrum of IN1-NO_2 in DMSO-d_6

Monoisotopic Mass, Even Electron Ions
15 formula(e) evaluated with 1 results within limits (up to 50 best isotopic matches for each mass)
Elements Used:
C: 0-9 H: 0-99 N: 0-3 O: 0-2
WH-ZHU
ZW-YCX-292 124 (1.412) Cm (122:124)

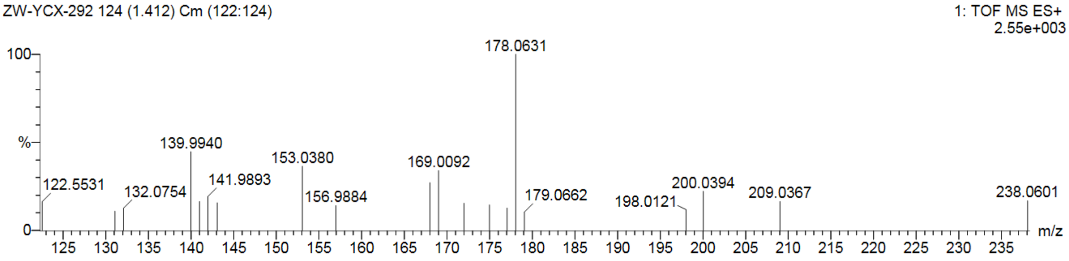

Minimum:				-1.5			
Maximum:		5.0	10.0	50.0			
Mass	Calc. Mass	mDa	PPM	DBE	i-FIT	i-FIT (Norm)	Formula
178.0631	178.0617	1.4	7.9	6.5	18.2	0.0	C8 H8 N3 O2

Supplementary Fig. 55. HRMS spectrum of IN1-NO_2

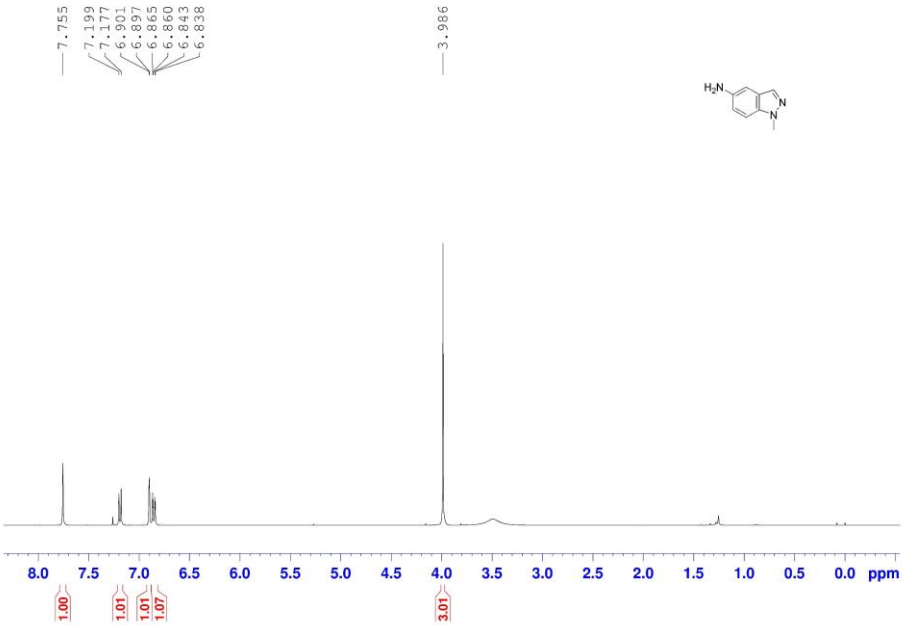

Supplementary Fig. 56. ^{1}H NMR spectrum of IN1-NH_2 in $CDCl_3$

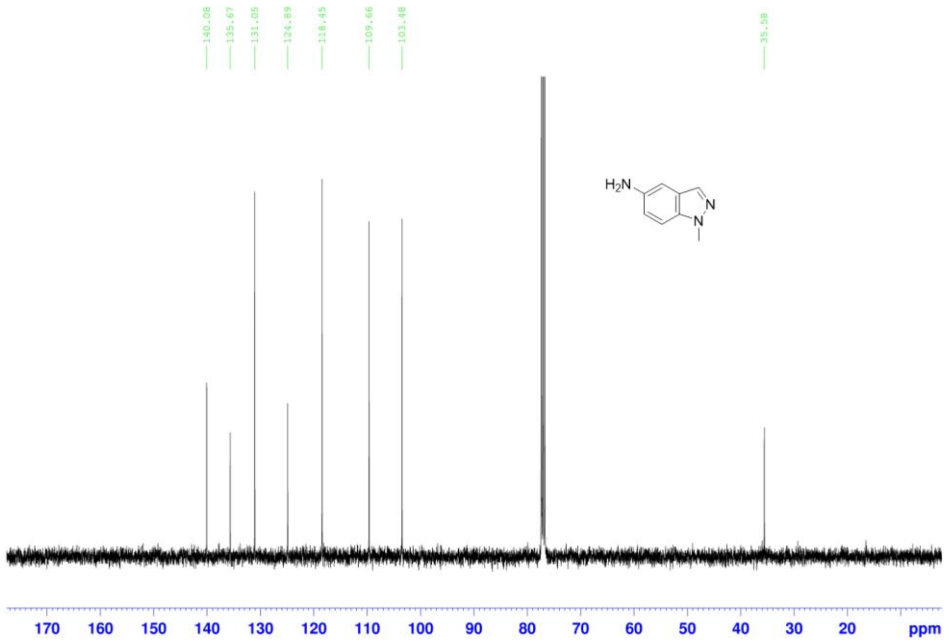

Supplementary Fig. 57. ^{13}C NMR spectrum of IN1-NH_2 in $CDCl_3$

Monoisotopic Mass, Even Electron Ions
5 formula(e) evaluated with 1 results within limits (up to 50 best isotopic matches for each mass)
Elements Used:
C: 0-8 H: 0-99 N: 0-3
WH-ZHU
ZW-YCX-293 25 (0.267) Cm (24:27)

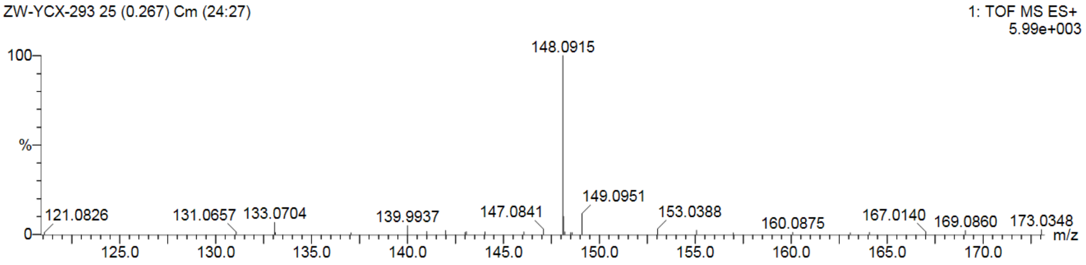

Mass	Calc. Mass	mDa	PPM	DBE	i-FIT	i-FIT (Norm)	Formula
Minimum:				-1.5			
Maximum:		5.0	40.0	50.0			
148.0915	148.0875	4.0	27.0	5.5	57.9	0.0	C8 H10 N3

Supplementary Fig. 58. HRMS spectrum of IN1-NH_2

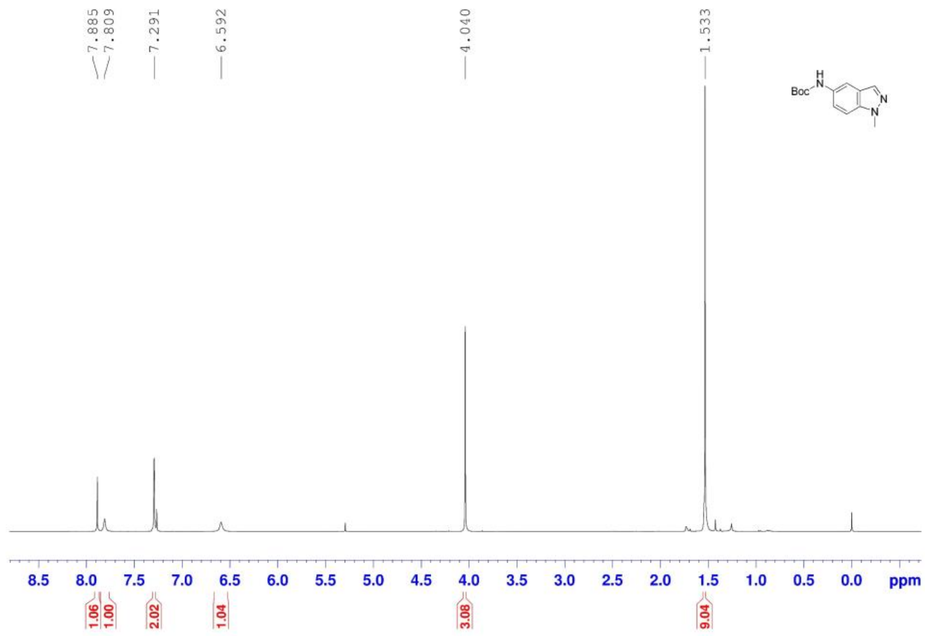

Supplementary Fig. 59. ^{1}H NMR spectrum of IN1-Boc in $CDCl_3$

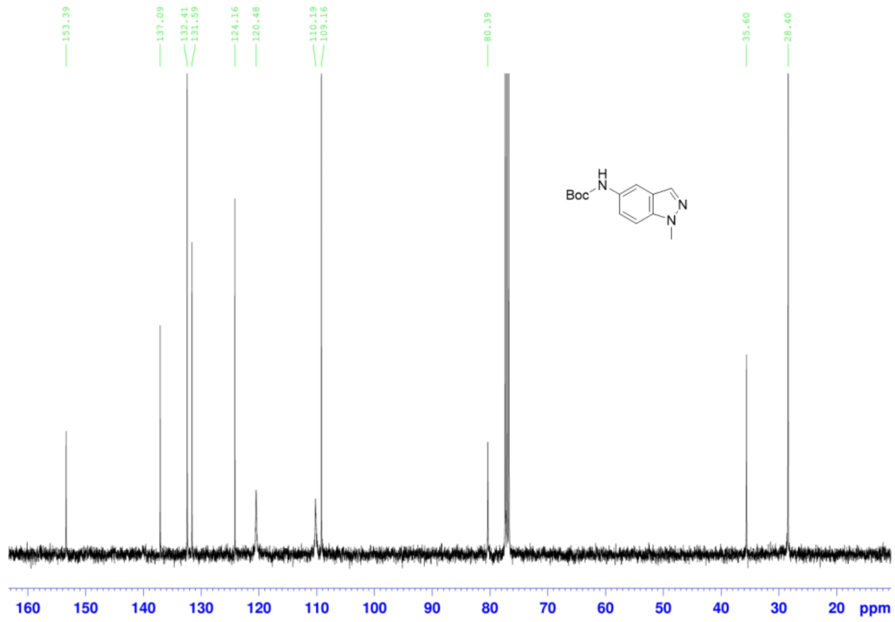

Supplementary Fig. 60. ^{13}C NMR spectrum of IN1-Boc in $CDCl_3$

Monoisotopic Mass, Even Electron Ions
21 formula(e) evaluated with 1 results within limits (up to 1 closest results for each mass)
Elements Used:
C: 0-13 H: 0-20 N: 0-3 O: 0-2 Na: 0-1

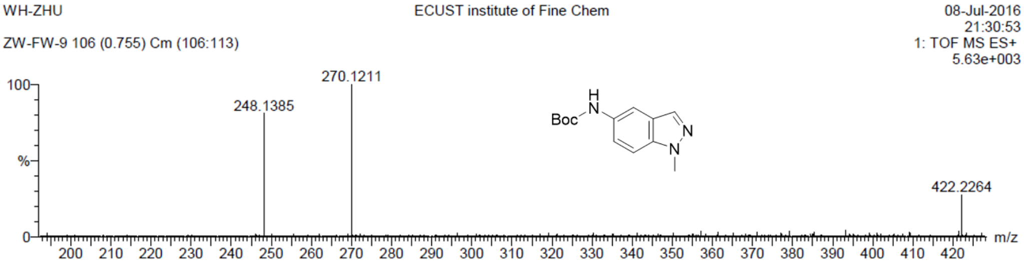

Minimum:				-1.5			
Maximum:		300.0	50.0	100.0			
Mass	Calc. Mass	mDa	PPM	DBE	i-FIT	i-FIT (Norm)	Formula
270.1211	270.1218	-0.7	-2.6	6.5	261.6	0.0	C13 H17 N3 O2 Na

Supplementary Fig. 61. HRMS spectrum of IN1-Boc

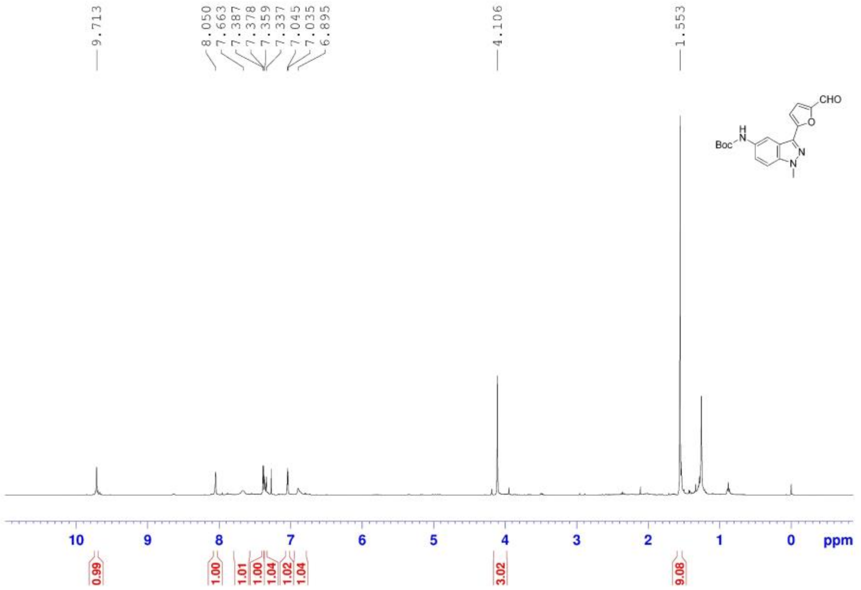

Supplementary Fig. 62. ^{1}H NMR spectrum of AL-IN1-Boc in $CDCl_3$

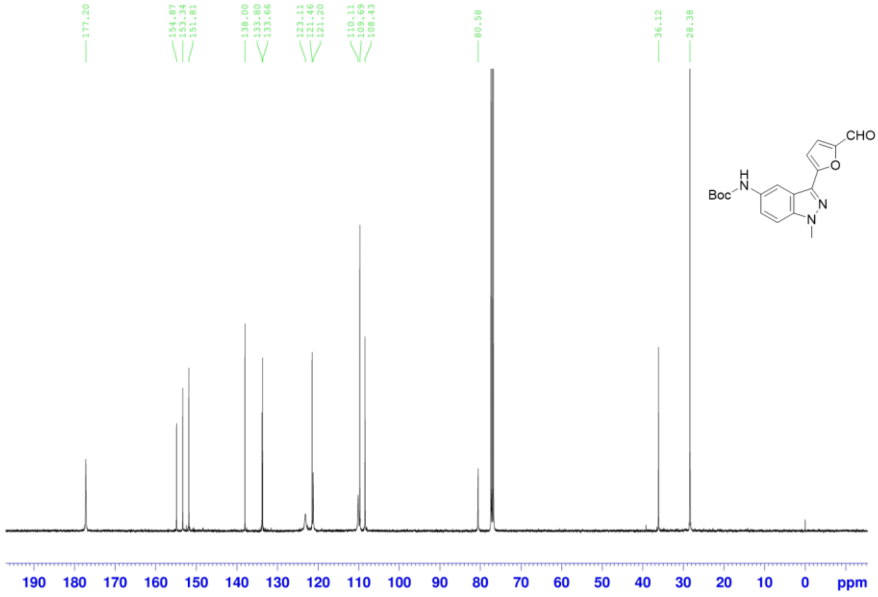

Supplementary Fig. 63. ^{13}C NMR spectrum of AL-IN1-Boc in $CDCl_3$

Monoisotopic Mass, Even Electron Ions
44 formula(e) evaluated with 1 results within limits (up to 1 closest results for each mass)
Elements Used:
C: 0-18 H: 0-135 N: 0-3 O: 0-4 Na: 0-1

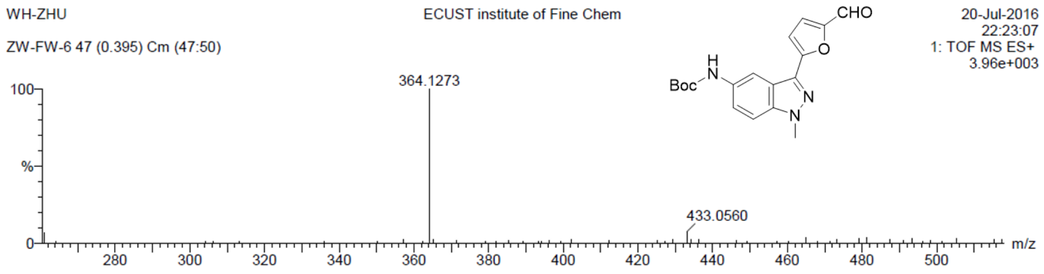

Minimum:				-1.5			
Maximum:		300.0	50.0	100.0			
Mass	Calc. Mass	mDa	PPM	DBE	i-FIT	i-FIT (Norm)	Formula
364.1273	364.1273	0.0	0.0	10.5	19.5	0.0	C18 H19 N3 O4 Na

Supplementary Fig. 64. HRMS spectrum of AL-IN1-Boc

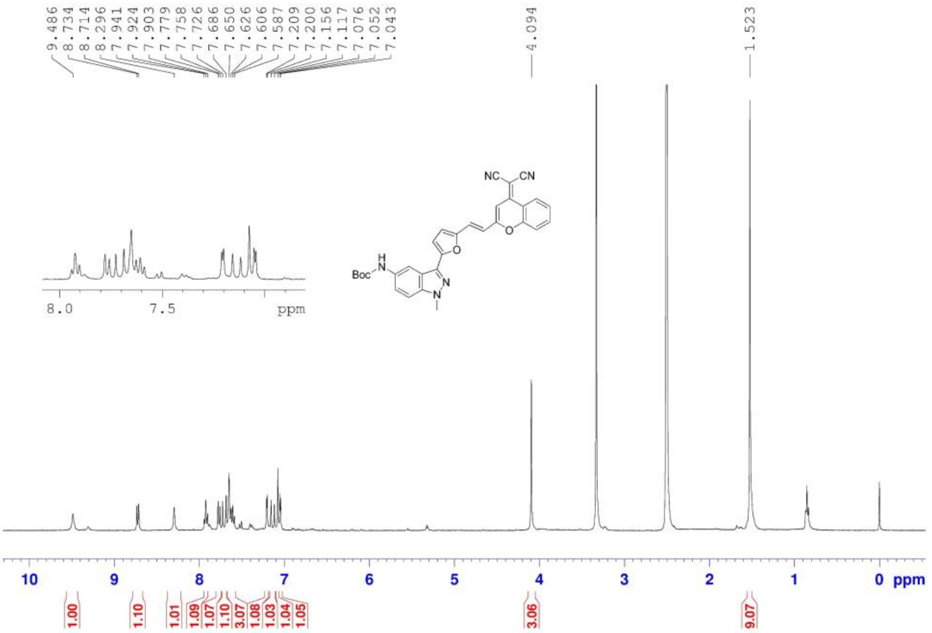

Supplementary Fig. 65. ^{1}H NMR spectrum of DCM-IN1-Boc in DMSO-d_6

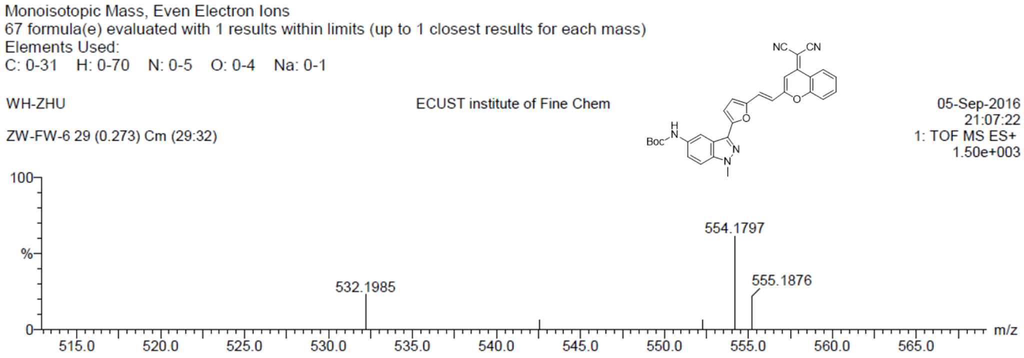

Minimum:				-1.5			
Maximum:		300.0	50.0	100.0			
Mass	Calc. Mass	mDa	PPM	DBE	i-FIT	i-FIT (Norm)	Formula
554.1797	554.1804	-0.7	-1.3	21.5	33.1	0.0	C31 H25 N5 O4 Na

Supplementary Fig. 66. HRMS spectrum of DCM-IN1-Boc

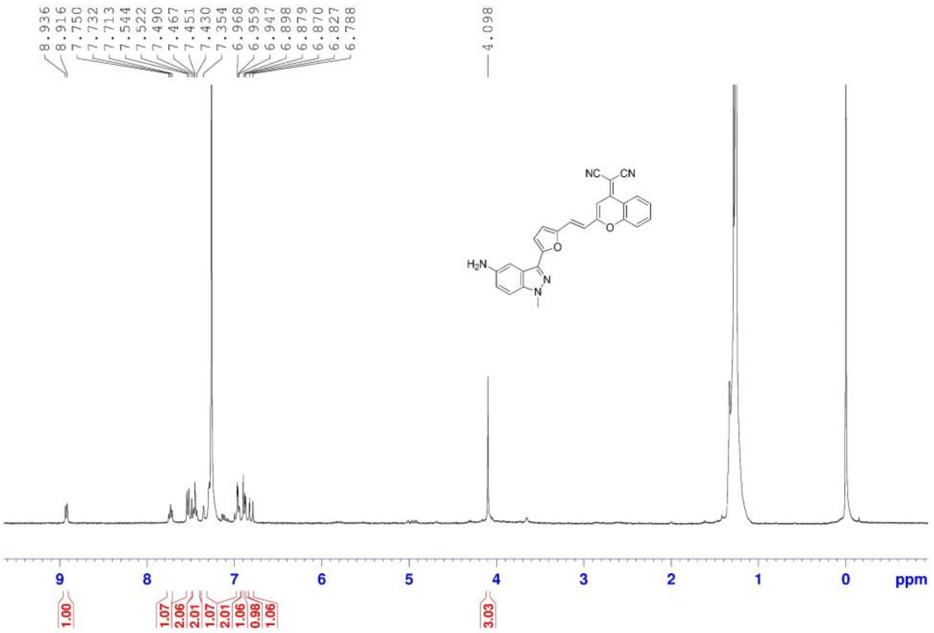

Supplementary Fig. 67. ^1H NMR spectrum of DCM-IN1-NH_2 in $CDCl_3$

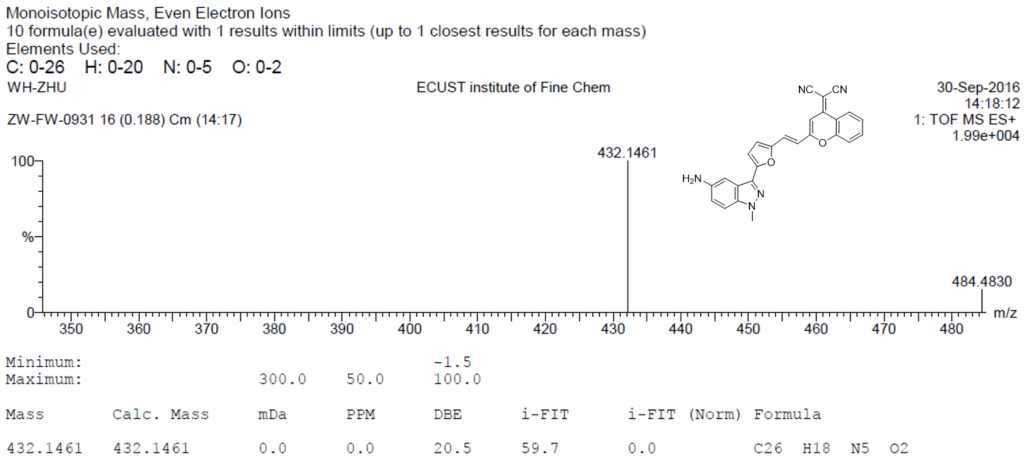

Supplementary Fig. 68. HRMS spectrum of DCM-IN1-NH_2

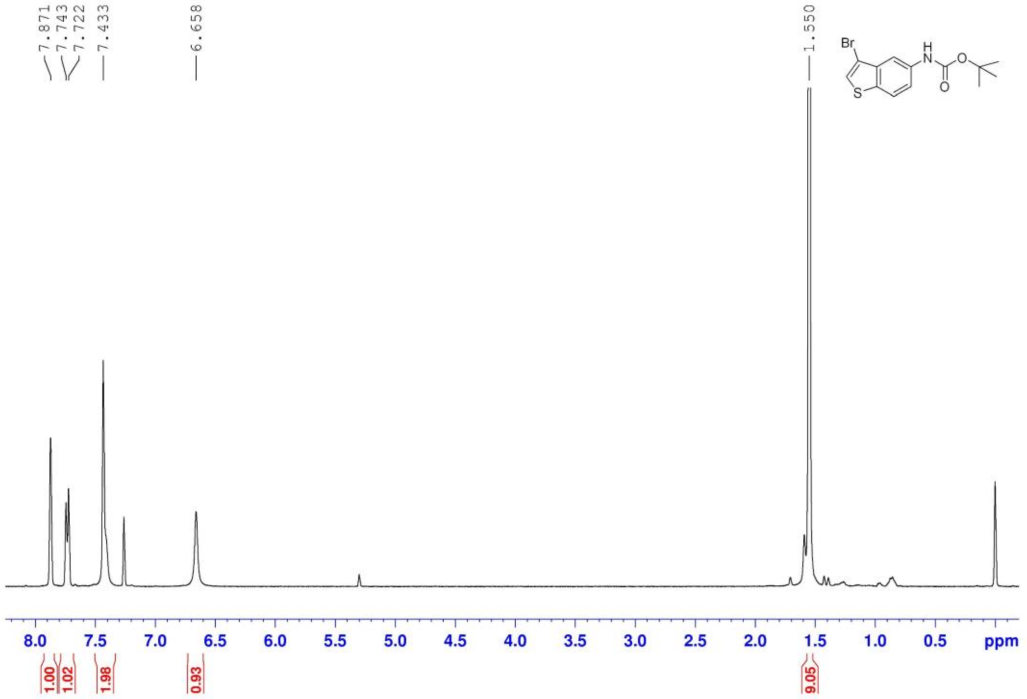

Supplementary Fig. 69. ^{1}H NMR spectrum of Br-TB-Boc in $CDCl_3$

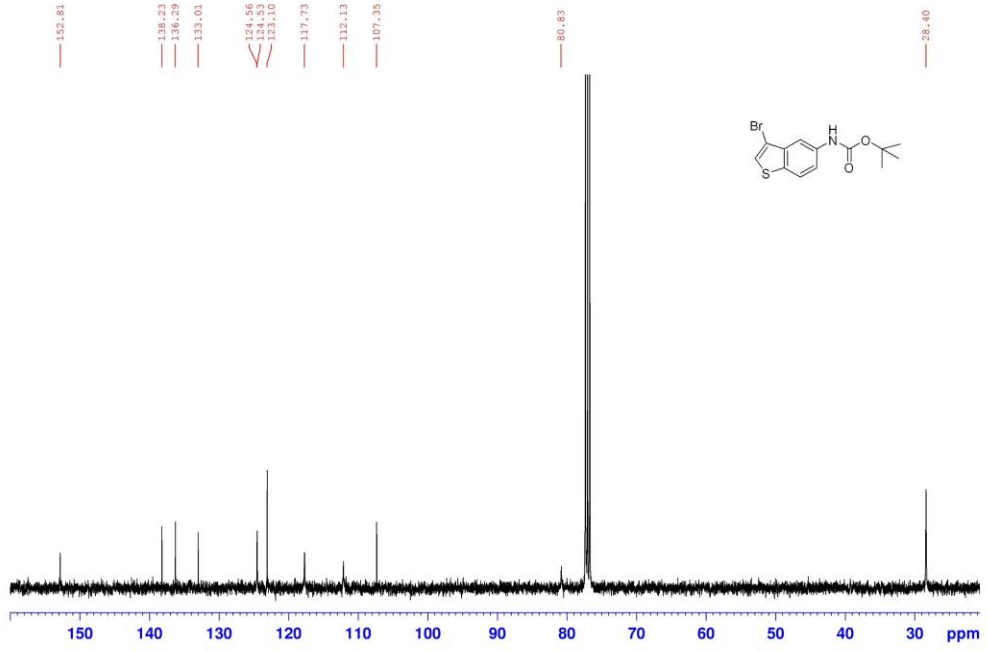

Supplementary Fig. 70. ^{13}C NMR spectrum of Br-TB-Boc in $CDCl_3$

Monoisotopic Mass, Even Electron Ions
22 formula(e) evaluated with 1 results within limits (up to 50 closest results for each mass)
Elements Used:
C: 0-13 H: 0-14 N: 0-1 O: 0-2 S: 0-1 Br: 0-1

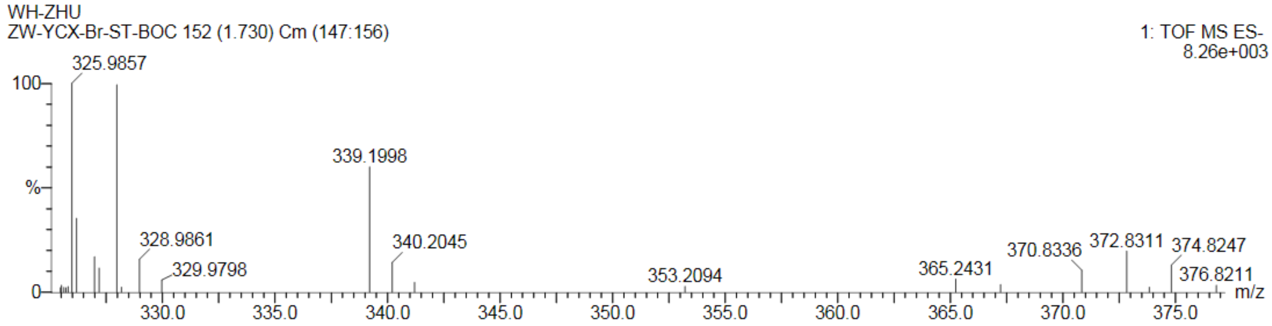

Minimum:				-1.5			
Maximum:		5.0	10.0	50.0			
Mass	Calc. Mass	mDa	PPM	DBE	i-FIT	i-FIT (Norm)	Formula
325.9857	325.9850	0.7	2.1	7.5	49.2	0.0	C13 H13 N O2 S Br

Supplementary Fig. 71. HRMS spectrum of Br-TB-Boc

Monoisotopic Mass, Even Electron Ions
67 formula(e) evaluated with 1 results within limits (up to 50 closest results for each mass)
Elements Used:
C: 0-18 H: 0-17 N: 0-1 O: 0-4 S: 0-1 Na: 0-3

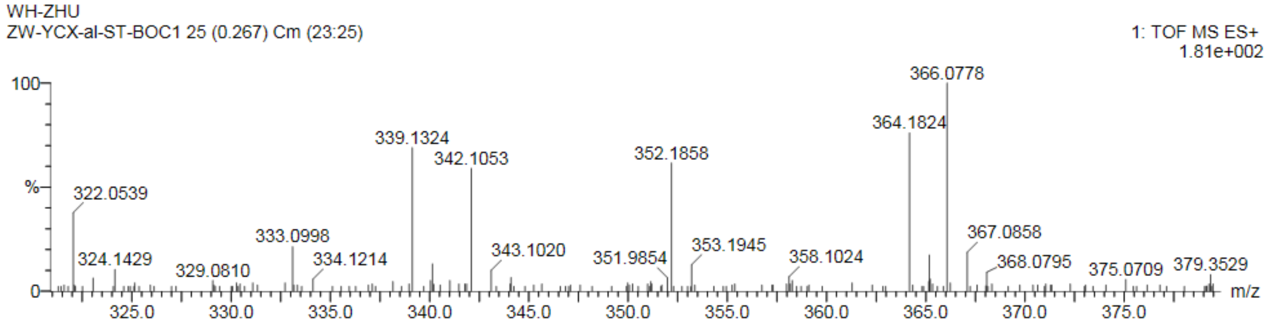

Minimum:				-1.5			
Maximum:		5.0	10.0	50.0			
Mass	Calc. Mass	mDa	PPM	DBE	i-FIT	i-FIT (Norm)	Formula
366.0778	366.0776	0.2	0.5	10.5	27.9	0.0	C18 H17 N O4 S Na

Supplementary Fig. 72. HRMS spectrum of AL-TB-Boc

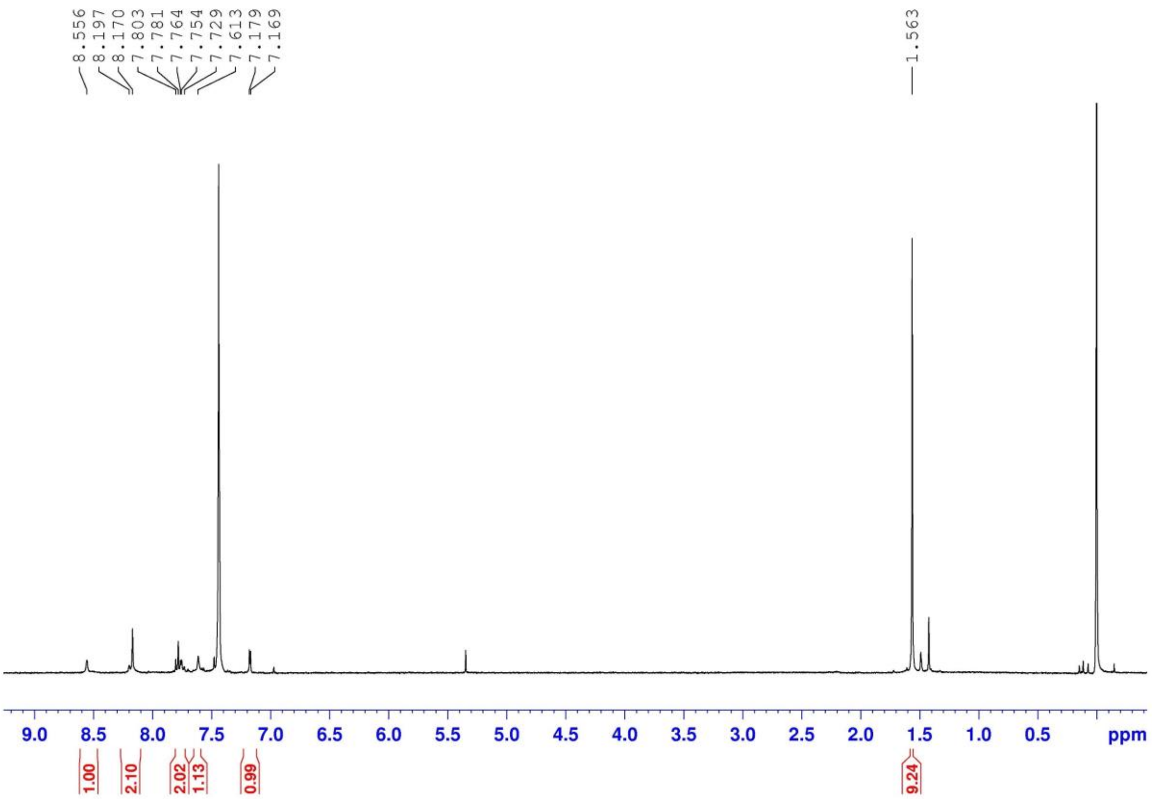

Supplementary Fig. 73. ^{1}H NMR spectrum of DC-TB-Boc in $CDCl_3$

Monoisotopic Mass, Even Electron Ions
99 formula(e) evaluated with 1 results within limits (up to 50 closest results for each mass)
Elements Used:
C: 0-21 H: 0-17 N: 0-3 O: 0-3 S: 0-1 Na: 0-3

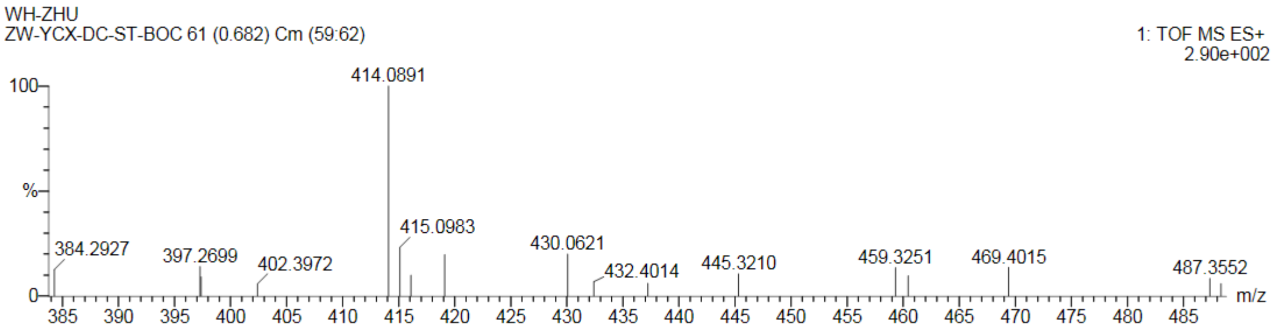

Minimum:				-1.5			
Maximum:		5.0	10.0	50.0			
Mass	Calc. Mass	mDa	PPM	DBE	i-FIT	i-FIT (Norm)	Formula
414.0891	414.0888	0.3	0.7	14.5	9.3	0.0	C21 H17 N3 O3 S Na

Supplementary Fig. 74. HRMS spectrum of DC-TB-Boc

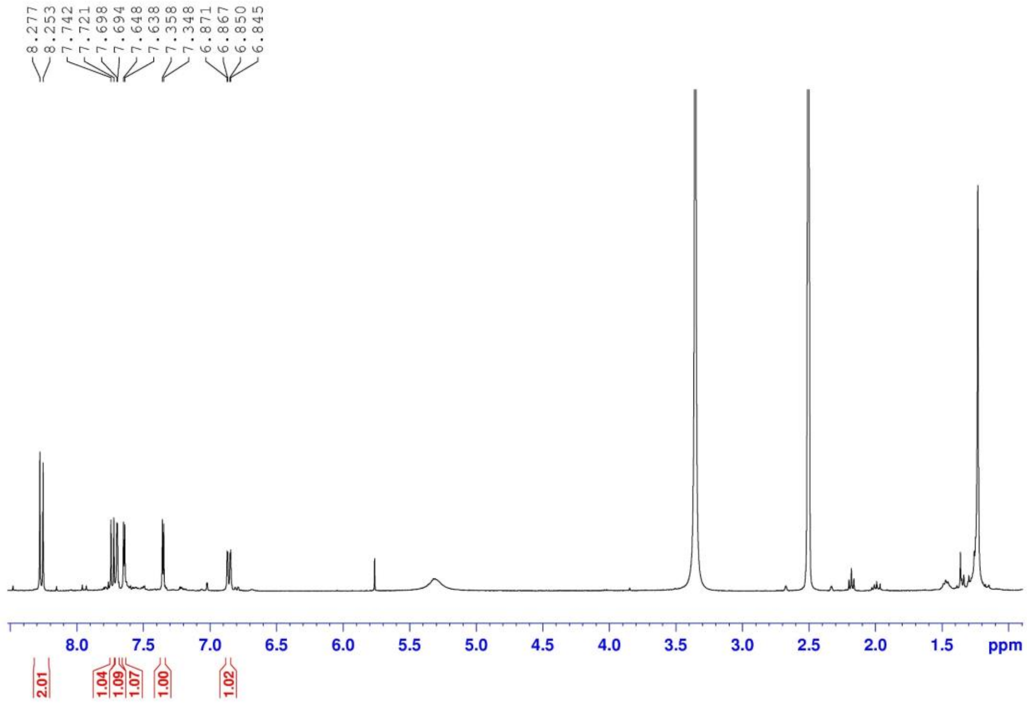

Supplementary Fig. 75. ^{1}H NMR spectrum of DC-TB-NH_2 in DMSO-d_6

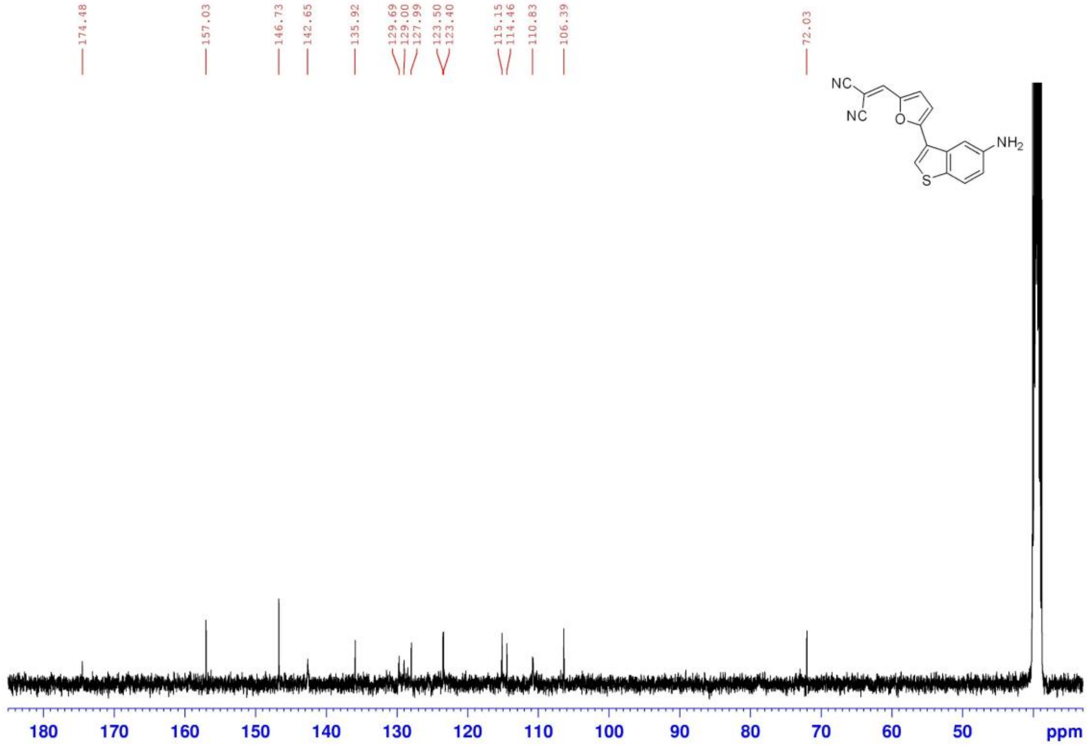

Supplementary Fig. 76. ^{13}C NMR spectrum of DC-TB-NH2 in DMSO-d_6

Monoisotopic Mass, Even Electron Ions
11 formula(e) evaluated with 1 results within limits (up to 50 closest results for each mass)
Elements Used:
C: 0-16 H: 0-10 N: 0-3 O: 0-1 S: 0-1
WH-ZHU
ZW-YCX-DM-TB-NH2 57 (0.635) Cm (56:57) 1: TOF MS ES+
5.08e+003

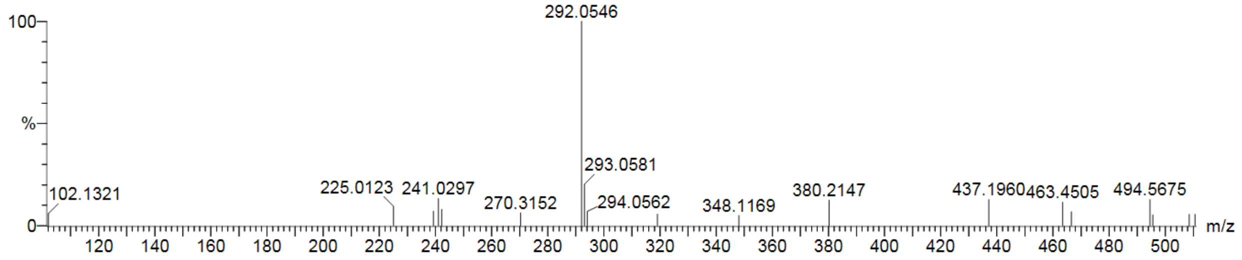

		mDa	PPM	DBE	i-FIT	i-FIT (Norm)	Formula
Minimum:				-1.5			
Maximum:		5.0	4.0	50.0			
**Mass**	**Calc. Mass**	**mDa**	**PPM**	**DBE**	**i-FIT**	**i-FIT (Norm)**	**Formula**
292.0546	292.0545	0.1	0.3	13.5	8.0	0.0	C16 H10 N3 O S

Supplementary Fig. 77. HRMS spectrum of DC-TB-NH_2

Supplementary Tables

Supplementary Table 1. Crystal data and structure refinement for IN-NH_2. The X-ray crystallographic coordinates for IN-NH_2 have been deposited at the Cambridge Crystallographic Data Centre (CCDC), under deposition number: 2041870.

Identification code	IN-NH_2
Empirical formula	$C_8 H_9 N_3$
Formula weight	147.18
Temperature	293(2) K
Wavelength	0.71073 Å
Crystal system	Orthorhombic
Space group	P c a 21
Unit cell dimensions	a = 13.9585(8) Å α = 90°.
	b = 7.6178(3) Å β = 90°.
	c = 7.1646(3) Å γ = 90°.
Volume	761.83(6) Å^3
Z	4
Density (calculated)	1.283 Mg/m^3
Absorption coefficient	0.082 mm^{-1}
F(000)	312
Crystal size	0.200 x 0.150 x 0.120 mm^3
Theta range for data collection	2.919 to 25.984°.
Index ranges	-17<=h<=10, -9<=k<=9, -8<=l<=8
Reflections collected	3578
Independent reflections	1424 [R(int) = 0.0197]
Completeness to theta = 25.242°	98.4 %
Absorption correction	Semi-empirical from equivalents
Max. and min. transmission	0.7456 and 0.6596
Refinement method	Full-matrix least-squares on F^2
Data / restraints / parameters	1424 / 1 / 110
Goodness-of-fit on F^2	1.106
Final R indices [I>2sigma(I)]	R1 = 0.0315, wR2 = 0.0766
R indices (all data)	R1 = 0.0370, wR2 = 0.0815
Absolute structure parameter	0.6(10)
Extinction coefficient	0.11(4)
Largest diff. peak and hole	0.101 and -0.077 e.Å^{-3}

Supplementary Table 2. Crystal data and structure refinement for IN-Boc. The X-ray crystallographic coordinates for IN-Boc have been deposited at the Cambridge Crystallographic Data Centre (CCDC), under deposition number: 2041873.

Identification code	IN-Boc	
Empirical formula	$C_{13} H_{17} N_3 O_2$	
Formula weight	247.29	
Temperature	160.01 K	
Wavelength	0.71073 Å	
Crystal system	Orthorhombic	
Space group	Pbca	
Unit cell dimensions	a = 13.8377(6) Å	α = 90°.
	b = 14.0408(5) Å	β = 90°.
	c = 14.1533(5) Å	γ = 90°.
Volume	2749.88(18) $Å^3$	
Z	8	
Density (calculated)	1.195 Mg/m^3	
Absorption coefficient	0.083 mm^{-1}	
F(000)	1056	
Crystal size	0.33 x 0.25 x 0.2 mm^3	
Theta range for data collection	2.878 to 26.991°.	
Index ranges	-13<=h<=17, -16<=k<=17, -17<=l<=18	
Reflections collected	12335	
Independent reflections	2979 [R(int) = 0.0885]	
Completeness to theta = 25.242°	99.4 %	
Absorption correction	Semi-empirical from equivalents	
Max. and min. transmission	0.7461 and 0.3021	
Refinement method	Full-matrix least-squares on F^2	
Data / restraints / parameters	2979 / 0 / 167	
Goodness-of-fit on F^2	0.967	
Final R indices [I>2sigma(I)]	R1 = 0.0701, wR2 = 0.1814	
R indices (all data)	R1 = 0.0873, wR2 = 0.1984	
Extinction coefficient	n/a	
Largest diff. peak and hole	0.399 and -0.336 e.$Å^{-3}$	

Supplementary Table 3. Crystal data and structure refinement for IN1-Boc. The X-ray crystallographic coordinates for IN1-Boc have been deposited at the Cambridge Crystallographic Data Centre (CCDC), under deposition number: 2041871.

Identification code	IN1-Boc	
Empirical formula	$C_{13} H_{17} N_3 O_2$	
Formula weight	247.29	
Temperature	160.02 K	
Wavelength	0.71073 Å	
Crystal system	Orthorhombic	
Space group	Pbca	
Unit cell dimensions	a = 12.8091(5) Å	α = 90°.
	b = 12.5740(4) Å	β = 90°.
	c = 16.5762(5) Å	γ = 90°.
Volume	2669.79(16) $Å^3$	
Z	8	
Density (calculated)	1.230 Mg/m^3	
Absorption coefficient	0.085 mm^{-1}	
F(000)	1056	
Crystal size	0.25 x 0.1 x 0.05 mm^3	
Theta range for data collection	2.457 to 30.503°.	
Index ranges	-18<=h<=15, -15<=k<=17, -23<=l<=23	
Reflections collected	29525	
Independent reflections	4065 [R(int) = 0.1142]	
Completeness to theta = 25.242°	99.8 %	
Absorption correction	Semi-empirical from equivalents	
Max. and min. transmission	0.7461 and 0.4179	
Refinement method	Full-matrix least-squares on F^2	
Data / restraints / parameters	4065 / 0 / 167	
Goodness-of-fit on F^2	1.063	
Final R indices [I>2sigma(I)]	R1 = 0.0702, wR2 = 0.1511	
R indices (all data)	R1 = 0.0995, wR2 = 0.1684	
Extinction coefficient	n/a	
Largest diff. peak and hole	0.315 and -0.331 e.$Å^{-3}$	

Supplementary Table 4. Crystal data and structure refinement for DC-IN-NH_2. The X-ray crystallographic coordinates for DC-IN-NH_2 have been deposited at the Cambridge Crystallographic Data Centre (CCDC), under deposition number: 2041872.

Identification code	DC-IN-NH_2	
Empirical formula	$C_{16} H_{11} N_5 O$	
Formula weight	289.30	
Temperature	293(2) K	
Wavelength	0.71073 Å	
Crystal system	Orthorhombic	
Space group	P 21 21 21	
Unit cell dimensions	a = 6.807(2) Å	α = 90°.
	b = 13.763(5) Å	β = 90°.
	c = 15.414(5) Å	γ = 90°.
Volume	1443.9(8) Å^3	
Z	4	
Density (calculated)	1.331 Mg/m^3	
Absorption coefficient	0.089 mm^{-1}	
F(000)	600	
Crystal size	0.120 x 0.080 x 0.040 mm^3	
Theta range for data collection	2.643 to 25.500°.	
Index ranges	-8<=h<=8, -16<=k<=16, -18<=l<=18	
Reflections collected	20143	
Independent reflections	2685 [R(int) = 0.0937]	
Completeness to theta = 25.242°	99.7 %	
Absorption correction	Semi-empirical from equivalents	
Max. and min. transmission	0.7456 and 0.6536	
Refinement method	Full-matrix least-squares on F^2	
Data / restraints / parameters	2685 / 0 / 209	
Goodness-of-fit on F^2	1.062	
Final R indices [I>2sigma(I)]	R1 = 0.0561, wR2 = 0.1269	
R indices (all data)	R1 = 0.1107, wR2 = 0.1579	
Absolute structure parameter	0.4(10)	
Extinction coefficient	0.017(6)	
Largest diff. peak and hole	0.154 and -0.138 e.Å^{-3}	

Supplementary Table 5. Crystal data and structure refinement for DC-IN-Boc. The X-ray crystallographic coordinates for DC-IN-Boc have been deposited at the Cambridge Crystallographic Data Centre (CCDC), under deposition number: 2041875.

Identification code	DC-IN-Boc	
Empirical formula	$C_{21} H_{19} N_5 O_3$	
Formula weight	389.41	
Temperature	293(2) K	
Wavelength	0.71073 Å	
Crystal system	Monoclinic	
Space group	P 21/m	
Unit cell dimensions	a = 11.211(3) Å	α = 90°.
	b = 6.7154(16) Å	β = 95.461(6)°.
	c = 12.960(3) Å	γ = 90°.
Volume	971.4(4) Å^3	
Z	2	
Density (calculated)	1.331 Mg/m^3	
Absorption coefficient	0.092 mm^{-1}	
F(000)	408	
Crystal size	0.190 x 0.050 x 0.040 mm^3	
Theta range for data collection	2.296 to 26.000°.	
Index ranges	-13<=h<=13, -8<=k<=8, -15<=l<=15	
Reflections collected	9048	
Independent reflections	2068 [R(int) = 0.0673]	
Completeness to theta = 25.242°	99.8 %	
Absorption correction	Semi-empirical from equivalents	
Max. and min. transmission	0.7456 and 0.6683	
Refinement method	Full-matrix least-squares on F^2	
Data / restraints / parameters	2068 / 0 / 199	
Goodness-of-fit on F^2	1.068	
Final R indices [I>2sigma(I)]	R1 = 0.0524, wR2 = 0.1006	
R indices (all data)	R1 = 0.0988, wR2 = 0.1245	
Extinction coefficient	0.044(6)	
Largest diff. peak and hole	0.179 and -0.146 e.Å^{-3}	

Supplementary Table 6. Crystal data and structure refinement for DCM-IN-NH_2. The X-ray crystallographic coordinates for DCM-IN-NH_2 have been deposited at the Cambridge Crystallographic Data Centre (CCDC), under deposition number: 2041874.

Identification code	DCM-IN-NH_2	
Empirical formula	$C_{26} H_{17} N_5 O_2$	
Formula weight	431.45	
Temperature	293(2) K	
Wavelength	0.71073 Å	
Crystal system	Orthorhombic	
Space group	P b c a	
Unit cell dimensions	a = 14.015(5) Å	α = 90°.
	b = 16.741(6) Å	β = 90°.
	c = 18.395(7) Å	γ = 90°.
Volume	4316(3) $Å^3$	
Z	8	
Density (calculated)	1.328 Mg/m^3	
Absorption coefficient	0.088 mm^{-1}	
F(000)	1792	
Crystal size	0.120 x 0.080 x 0.050 mm^3	
Theta range for data collection	2.214 to 24.995°.	
Index ranges	-16<=h<=16, -19<=k<=19, -16<=l<=21	
Reflections collected	18341	
Independent reflections	3793 [R(int) = 0.1928]	
Completeness to theta = 25.242°	97.3 %	
Absorption correction	Semi-empirical from equivalents	
Max. and min. transmission	0.7456 and 0.5728	
Refinement method	Full-matrix least-squares on F^2	
Data / restraints / parameters	3793 / 0 / 300	
Goodness-of-fit on F^2	0.997	
Final R indices [I>2sigma(I)]	R1 = 0.1036, wR2 = 0.2273	
R indices (all data)	R1 = 0.2900, wR2 = 0.3405	
Extinction coefficient	n/a	
Largest diff. peak and hole	0.253 and -0.199 e.$Å^{-3}$	

Supplementary Methods

Theoretical calculation details. M06-2X/Def2-SVP and CAM-B3LYP/def2SVP calculations were carried out using Gaussian 16 A[2]. Density functional theory (DFT) and time-dependent DFT (TD-DFT) were employed to investigate the fluorescence (quenching) mechanism of all compounds. All structural optimizations in the ground and excited states were performed using M06-2X functional[3] and Def2-SVP basis set[4]. Solvation effects in dimethyl sulfoxide (DMSO) were taken into account using the SMD model[5]. Frequency calculations were performed to confirm that we obtained stable structures without imaginary vibrational frequencies. The potential energy surface (PES) in the first excited state (S_1) was calculated at the same level (M06-2X/Def2-SVP) using relaxed scans with the corrected Linear Response (cLR) solvent formalism around the bond connecting the indazole to the furan moieties. Docking calculation was carried out using AutoDock 4.2.6.

Euthanasia of animals. Euthanasia was performed per the recommendations of the "AVMA Guidelines for the Euthanasia of Animals: 2013 edition".[6] In Section 2.2 "Small laboratory and wild-caught rodents (mice, rats, hamsters, guinea pigs, gerbils, degus, cotton rats)" of this Guidelines: "Barbiturates and barbituric acid derivatives—Injectable barbiturates act quickly and smoothly to render rodents unconscious." Thus, animals on this study were euthanized by injected to an overdose of barbiturates, which was consistent with the AVMA Guidelines of Euthanasia for the Euthanasia of Animals: 2013 Edition.

Tissue homogenates: tissue subcellular fractionation and protein extraction. After the euthanasia of mice, the organs (including heart, liver, spleen, lung, and kidney) were isolated by the standard anatomy atlas[7]. Also, tissue homogenates were prepared by the standard method[8-10]. Tissue pieces were put into 4.5 mL PE pipes, and added with ice-cold 500 μL RIPA buffer (RIPA lysis buffer, 10×, cat. no. 20-188, Merck, Darmstadt, Germany) containing cocktail of protease inhibitors (Protease Inhibitor Cocktail Set, Calbiochem, cat. no. 539131, Merck, Darmstadt, Germany). Then, they were sonicated for 3 min and incubated for 30 minutes at 0 °C. The resultant homogenate was centrifuged at 13400 g for 1 hour at 4°C the supernatant was collected and used for proceeding experiments. Homogenates were kept on ice until they were used for the assays which were completed on the same day as homogenization. In fluorescence assays of Fig 6k, homogenates were diluted in PBS buffer (homogenate:PBS:DMSO = 4.5:4.5:1, v:v:v, pH 7.4). These solutions were added with Lighter EW Tracker (10 μM) and incubated for 30 min at 37 °C. Then, the fluorescence intensities at 700 nm were recorded (λ_{ex} = 500 nm).

Synthesis of DCM, DCM-Ac, and DCM-NH_2. The compounds DCM, DCM-Ac, and DCM-NH_2 were synthesized by the established procedures from our reported works[11].

Synthesis of IN-NO_2. I-NO_2 (1.00 g, 6.13 mmol) and KOH (3.36 g, 60.10 mmol) were dissolved in water (10 mL) under an argon atmosphere at room temperature. Then Me_2SO_4 (3.50 g, 27.80 mmol) was added dropwise. The

mixture was refluxed for 1 h under an argon atmosphere. The solution was filtered and the residue was purified by silica gel chromatography (CH_2Cl_2:PE = 1:1) to get the desired product IN-NO_2 (300 mg). Yield was 28%. ^{1}H NMR (400 MHz, DMSO-d_6, ppm): δ 4.25 (s, 3H, NCH_3-H), 7.77 (d, J = 8.0 Hz, 1H, phenyl-H), 8.00 (dd, J = 8.0 Hz, 1H, phenyl-H), 8.77 (s, 1H, imidazole-H), 8.88 (d, J = 4.0 Hz, 1H, phenyl-H). ^{13}C NMR (100 MHz, $CDCl_3$, ppm): δ 150.02, 143.08, 127.77, 120.40, 120.25, 119.22, 118.19, 40.96. Mass spectrometry (ESI-MS, m/z): $[M + H]^+$ calcd. for $[C_8H_7N_3O_2 + H]^+$ 178.0629; found 178.0629.

Synthesis of IN-NH_2. IN-NO_2 (100 mg, 0.56 mmol), iron powder (110 mg, 2.00 mmol), and NH_4Cl (75 mg, 1.41 mmol) were dissolved in mixture of methanol and water (16 mL, v:v = 3:1) under an argon atmosphere at room temperature. Then the mixture was refluxed for 4 h under an argon atmosphere. The solution was added to ethyl acetate (20 mL) and washed with deionized H_2O (5 mL × 5). The organic layer was dried over anhydrous Na_2SO_4, filtered and concentrated by evaporation. The residue was purified by silica gel chromatography (CH_2Cl_2) to get the product IN-NH_2 (70 mg). Yield was 84%.^{1}H NMR (400 MHz, $CDCl_3$, ppm): δ 4.14 (s, 3H, NCH_3-H), 6.75 (m, 1H, phenyl-H), 6.81 (dd, J = 8.0 Hz, 4.0 Hz, 1H, phenyl-H), 7.53(d, J = 8.8 Hz, 1H, phenyl-H), 7.62 (s, 1H, imidazole-H). ^{13}C NMR (100 MHz, $CDCl_3$, ppm): δ 145.50, 140.55, 122.82, 121.33, 120.42, 118.29, 99.70, 40.09. Mass spectrometry (ESI-MS, m/z): $[M + H]^+$ calcd. for $[C_8H_9N_3 + H]^+$ 148.0875; found 148.0871.

Synthesis of IN-Boc. IN-NH_2 (200 mg, 1.36 mmol) was dissolved in THF (5 mL) under an argon atmosphere at room temperature. Then $(Boc)_2O$ (296 mg, 1.36 mmol, mixed with 10 mL of THF) was added dropwise. Then the mixture was stirred overnight at room temperature. The solution was added to CH_2Cl_2 (20 mL) and washed with deionized H_2O (20 mL × 5). The organic layer was dried over anhydrous Na_2SO_4, filtered and concentrated by evaporation. The residue was purified by silica gel chromatography (CH_2Cl_2:PE = 1:1) to get desired product IN-Boc (235 mg). Yield was 70%. ^{1}H NMR (400 MHz, $CDCl_3$, ppm): δ 1.53 (s, 9H, -(CH_3)$_3$), 4.19 (s, 3H, NCH_3-H), 6.45 (s, 1H, imidazole-H), 7.00 (dd, J = 8.0 Hz, 1H, phenyl-H), 7.61 (d, J = 8.0 Hz, 1H, phenyl-H), 7.79 (s, 1H, phenyl-H), 7.89 (s, 1H, amide-H). ^{13}C NMR (100 MHz, $CDCl_3$, ppm): δ 153.22, 146.46, 132.04, 123.32, 122.14, 121.13, 111.87, 107.32, 80.30, 40.26, 28.39. Mass spectrometry (ESI-MS, m/z): $[M + Na]^+$ calcd. for $[C_{13}H_{17}N_3O_2 + Na]^+$ 270.1218; found 270.1222.

Synthesis of IN-Ac. IN-NH_2 (200 mg, 1.36 mmol) was dissolved in CH_2Cl_2 (5 mL) under an argon atmosphere at room temperature. Then $(CH_3CO)_2O$ (0.5 mL) was added dropwise. Then the mixture was stirred overnight at room temperature. The solution was added to CH_2Cl_2 (20 mL) and washed with deionized H_2O (20 mL × 5). The organic layer was dried over anhydrous Na_2SO_4, filtered and concentrated by evaporation. The residue was purified by silica gel chromatography (CH_2Cl_2:MeOH = 100:1) to get desired product IN-Ac (192 mg). Yield was 75%. ^{1}H NMR (400 MHz, $CDCl_3$, ppm): δ 2.20 (s, 3H, -CH_3), 4.19 (s, 3H, NCH_3-H), 7.04 (dd, J = 9.2 Hz, 1H, phenyl-H), 7.34 (s, 1H, amide-H), 7.62 (d, J = 9.2 Hz, 1H, phenyl-H), 7.83 (s, 1H, imidazole-H), 8.14 (s, 1H, phenyl-H). ^{13}C NMR (100 MHz, $CDCl_3$, ppm): δ 168.43, 146.75, 131.53, 123.83, 121.96, 121.41, 117.94, 109.67, 10.33, 24.59. Mass

spectrometry (ESI-MS, m/z): $[M + H]^+$ calcd. for $[C_{13}H_{17}N_3O_2 + H]^+$ 190.0980; found 190.0978.

Synthesis of AL-SIN-Boc. IN-Boc (400 mg, 1.62 mmol), $Cu(OAc)_2$ H_2O (486 mg, 2.43 mmol), thiophene-2-carbaldehyde (544 mg, 4.86 mmol), pyridine (0.2 mL), and $Pd(PPh_3)_4$ (94 mg, 0.08 mmol) were dissolved in 1,4-dioxane (4 mL) under an argon atmosphere at room temperature. Then the mixture was refluxed for 24 h under an argon atmosphere. The solution was filtered and concentrated by evaporation. The residue was purified by silica gel chromatography (CH_2Cl_2) to get the product AL-SIN-Boc (252 mg). Yield was 44%. ^{1}H NMR (400 MHz, $CDCl_3$, ppm): δ 1.53 (s, 9H, -(CH_3)$_3$), 4.32 (s, 3H, NCH_3-H), 6.63 (s, 1H, phenyl-H), 7.11 (dd, 1H, phenyl-H), 7.44 (d, J = 4.0 Hz, 1H, furan-H), 7.65 (d, J = 8.0 Hz, 1H, phenyl-H), 7.88 (d, J = 4.0 Hz, 1H, furan-H), 8.01 (s, 1H, amide-H), 9.98 (s, 1H, C*H*O-H). Mass spectrometry (ESI-MS, m/z): $[M + H]^+$ calcd. for $[C_{18}H_{19}N_3O_3S + H]^+$ 358.1225; found 358.1233.

Synthesis of DCM-SIN-Boc. AL-SIN-Boc (89 mg, 0.25 mmol), DCM (52 mg, 0.25 mmol), piperidine (0.2 mL), and acetic acid (0.1 mL) were dissolved in toluene (10 mL) under an argon atmosphere at room temperature. Then the mixture was refluxed for 6 h under an argon atmosphere. The solution was concentrated by evaporation. The residue was purified by silica gel chromatography (CH_2Cl_2) to get the product DCM-SIN-Boc (28 mg). Yield was 20%. ^{1}H NMR (400 MHz, DMSO-d_6, ppm): δ 1.49 (s, 9H, -(CH_3)$_3$), δ= 4.26 (s, 3H, NCH_3-H), 7.15 (s, 1H, phenyl-H), 7.30 (dd, J = 8.0 Hz, 1H, phenyl-H), 7.37 (d, J = 16.0 Hz, 1H, alkene-H), 7.58 (d, J = 4.0 Hz, 1H, thiophene-H), 7.59 (d, J = 8.0 Hz, 1H, phenyl-H), δ= 7.64 (t, J = 4.0 Hz, 1H, phenyl-H), 7.77 (d, J = 4.0 Hz, 1H, thiophene-H), 7.81 (d, J = 8.0 Hz, 1H, phenyl-H), 7.94 (t, J = 8.0 Hz, 1H, phenyl-H), 8.02 (d, J = 16.0 Hz, 1H, alkene-H), 8.14 (s, 1H, phenyl-H), 8.74 (dd, J = 8.0 Hz, 1H, phenyl-H), 9.42 (s, 1H, amide-H). Mass spectrometry (ESI-MS, m/z): $[M + Na]^+$ calcd. for $[C_{31}H_{25}N_5O_3S + Na]^+$ 548.1756; found 548.1754.

Synthesis of DCM-SIN-NH$_2$. DCM-SIN-Boc (20 mg, 0.04 mmol) was dissolved in CH_2Cl_2 (5 mL) under an argon atmosphere at room temperature. Then trifluoroacetic acid (TFA, 1 mL) was added dropwise. The mixture was stirred overnight at room temperature. The solution was concentrated by evaporation. The residue was purified by silica gel chromatography (CH_2Cl_2:EtOAc = 6:1) to get desired product DCM-SIN-NH$_2$ (10 mg). Yield was 56%. ^{1}H NMR (400 MHz, DMSO-d_6, ppm): δ 4.23 (s, 3H, NCH_3-H), 5.21 (s, 2H, NH_2-H), 6.85 (m, 2H, phenyl-H), 7.12 (s, 1H, phenyl-H), 7.28 (d, J = 16.0 Hz, 1H, alkene-H), 7.42 (d, J = 8.0 Hz, 1H, phenyl-H), 7.51 (d, J = 4.0 Hz, 1H, thiophene-H), 7.62 (t, J = 8.0 Hz, 1H, phenyl-H), 7.70 (d, J = 4.0 Hz, 1H, thiophene-H), 7.79 (d, J = 8.0 Hz, 1H, phenyl-H), 7.94 (t, J = 8.0 Hz, 1H, phenyl-H), 8.00 (d, J = 16.0 Hz, 1H, phenyl-H), 8.74 (d, J = 8.0 Hz, 1H, phenyl-H). Mass spectrometry (ESI-MS, m/z): $[M + H]^+$ calcd. for $[C_{26}H_{17}N_5OS + H]^+$ 448.1232; found 448.1230.

Synthesis of AL-IN-Boc. IN-Boc (200 mg, 0.81 mmol), $Cu(OAc)_2$ H_2O (242 mg, 1.20 mmol), furan-2-carbaldehyde (233 mg, 2.43 mmol), pyridine (0.2 mL), and $Pd(PPh_3)_4$ (47 mg, 0.04 mmol) were dissolved in 1,4-dioxane (4 mL) under an argon atmosphere at room temperature. Then the mixture was refluxed for 24 h under an argon atmosphere.

The solution was filtered and concentrated by evaporation. The residue was purified by silica gel chromatography (CH_2Cl_2:PE = 1:1) to get the product AL-IN-Boc (65 mg). Yield was 41%. ^{1}H NMR (400 MHz, $CDCl_3$, ppm): δ 1.55 (s, 9H, -(C*H*$_3$)$_3$), 4.46 (s, 3H, NC*H*$_3$-H), 6.63 (s, 1H, furan-H), 7.01 (d, J = 4.0 Hz, 1H, furan-H), 7.15 (dd, J = 8.0 Hz, 4.0 Hz, 1H, phenyl-H), 7.43 (d, J = 4.0 Hz, 1H, phenyl-H), 7.66 (s, 1H, phenyl-H), 8.11 (s, 1H, amide-H), 9.72 (s, 1H, C*H*O-H). ^{13}C NMR (100 MHz, $CDCl_3$, ppm): δ 176.90, 153.00, 151.88, 149.83, 145.19, 134.42, 132.85, 132.05, 128.47, 124.22, 121.84, 118.63, 110.49, 80.67, 40.91, 28.38. Mass spectrometry (ESI-MS, m/z): $[M + H]^+$ calcd. for $[C_{18}H_{19}N_3O_4 + H]^+$ 342.1454; found 342.1458.

Synthesis of DC-IN-Boc. AL-IN-Boc (100 mg, 0.3 mmol) and malononitrile (28 mg, 0.4 mmol) were dissolved in $CHCl_3$ (5 mL) under an argon atmosphere at room temperature. Then triethylamine (TEA, 0.2 mL) was added dropwise. The mixture was stirred overnight at room temperature. The solution was added to CH_2Cl_2 (20 mL) and washed with deionized H_2O (20 mL × 5). The organic layer was dried over anhydrous Na_2SO_4, filtered and concentrated by evaporation. The residue was purified by silica gel chromatography (CH_2Cl_2:EtOAc = 80:1) to get the desired product DC-IN-Boc (100 mg). Yield was 85%. ^{1}H NMR (400 MHz, DMSO-d_6, ppm): δ 1.50 (s, 9H, -(C*H*$_3$)$_3$), 4.42 (s, 3H, NC*H*$_3$-H), 7.30 (d, J = 4.0 Hz, 1H, Ph-H), 7.37 (d, J = 12.0 Hz, 1H, Ph-H), 7.66 (d, J = 12.0 Hz, 1H, Ph-H), 7.72 (d, J = 4.0 Hz, 1H, Ph-H), 8.14 (s, 1H, Ph-H), 8.32(s, 1H, Ph-H), 9.44 (s, 1H, amide-H). Mass spectrometry (ESI-MS, m/z): $[M + H]^+$ calcd. for $[C_{26}H_{17}N_5O_2 + H]^+$ 390.1566; found 390.1566.

Synthesis of DC-IN-NH$_2$. DC-IN-Boc (161 mg, 0.41 mmol) was dissolved in CH_2Cl_2 (10 mL) under an argon atmosphere at room temperature. Then trifluoroacetic acid (TFA, 8 mL) was added dropwise. The mixture was stirred overnight at room temperature. The solution was concentrated by evaporation. The residue was purified by silica gel chromatography (CH_2Cl_2) to get the desired product DC-IN-NH$_2$ (70 mg). Yield was 59%. ^{1}H NMR (400 MHz, DMSO-d_6, ppm): δ 4.35 (s, 3H, NC*H*$_3$-H), 5.24 (s, 2H, N*H*$_2$-H), 6.88 (d, J = 8.0 Hz, 1H, Ph-H), 7.00 (s, 1H, Ph-H), 7.24 (d, J = 4.0 Hz, 1H, Ph-H), 7.49 (d, J = 8.0 Hz, 1H, Ph-H), 7.68 (d, J = 4.0 Hz, 1H, Ph-H), 8.20 (s, 1H, Ph-H). Mass spectrometry (ESI-MS, m/z): $[M + H]^+$ calcd. for $[C_{16}H_{12}N_5O + H]^+$ 290.1042; found 290.1042.

Synthesis of DC-IN-Ac. DC-IN-NH$_2$ (40 mg, 0.14 mmol) was dissolved in CH_2Cl_2 (5 mL) under an argon atmosphere at room temperature. Then $(CH_3CO)_2O$ (0.5 mL) was added dropwise. Then the mixture was stirred overnight at room temperature. The solution was added to CH_2Cl_2 (20 mL) and washed with deionized H_2O (20 mL × 5). The organic layer was dried over anhydrous Na_2SO_4, filtered and concentrated by evaporation. The residue was purified by silica gel chromatography (CH_2Cl_2) to get the desired product DC-IN-Ac (18 mg). Yield was 39%. ^{1}H NMR (400 MHz, DMSO-d_6, ppm): δ 2.08 (s, 3H, C*H*$_3$-H), 4.42 (s, 3H, NC*H*$_3$-H), 7.31 (d, J = 4.0 Hz, 1H, furan-H), 7.40 (d, J = 8.0 Hz, 1H, phenyl-H), 7.69 (s, 1H, phenyl-H), 7.72 (d, J = 4.0 Hz, 1H, furan-H), 8.32 (s, 1H, alkene-H), 8.36 (s, 1H, phenyl-H), 10.1(s, 1H, amide-H). Mass spectrometry (ESI-MS, m/z): $[M + H]^+$ calcd. for $[C_{18}H_{13}N_5O_2 + H]^+$ 332.1147; found 332.1147.

Synthesis of DCM-IN-Boc. AL-IN-Boc (57 mg, 0.17 mmol), DCM (52 mg, 0.25 mmol), piperidine (0.2 mL), and acetic acid (0.1 mL) were dissolved in Toluene (10 mL) under an argon atmosphere at room temperature. Then the mixture was refluxed for 6 h under an argon atmosphere. The solution was concentrated by evaporation. The residue was purified by silica gel chromatography (CH_2Cl_2) to get the product DCM-IN-Boc (28 mg). Yield was 31%. ^{1}H NMR (400 MHz, DMSO-d_6, ppm): δ 1.51 (s, 9H, -(CH_3)$_3$), 4.38 (s, 3H, NCH_3-H), 7.09 (s, 1H, phenyl-H), 7.15 (d, J = 4.0 Hz, 1H, furan-H), 7.22 (d, J = 16.0 Hz, 1H, alkene-H), 7.28 (d, J = 4.0 Hz, 1H, furan-H), 7.44 (d, J = 8.0 Hz, 1H, phenyl-H), 7.62 (t, J = 8.0 Hz, 2H, phenyl-H), 7.74 (d, J = 16 Hz, 1H, alkene-H), 7.79 (d, J = 8.0 Hz, 1H, phenyl-H), 7.94 (t, J = 8.0 Hz, 1H, phenyl-H), 8.23 (s, 1H, phenyl-H), 8.74 (d, J = 8.0 Hz, 1H, phenyl-H), 9.47 (s, 1H, amide-H). Mass spectrometry (ESI-MS, m/z): $[M + Na]^+$ calcd. for $[C_{31}H_{25}N_5O_4 + Na]^+$ 554.1804; found 554.1804.

Synthesis of DCM-IN-NH$_2$. DC-IN-Boc (20 mg, 0.04 mmol) was dissolved in CH_2Cl_2 (10 mL) under an argon atmosphere at room temperature. Then trifluoroacetic acid (TFA, 1 mL) was added dropwise. The mixture was stirred overnight at room temperature. The solution was concentrated by evaporation. The residue was purified by silica gel chromatography (CH_2Cl_2) to get desired product DCM-IN-NH_2 (10 mg). Yield was 59%. ^{1}H NMR (400 MHz, DMSO-d_6, ppm): δ 4.31 (s, 3H, NCH_3-H), 5.32 (s, 2H, NH_2-H), 6.86 (d, J = 8.0 Hz, 1H, phenyl-H), 7.07 (m, 2H, phenyl-H, furan-H), 7.21 (d, J = 16.0 Hz, 1H, alkene-H), 7.23 (s, 1H, phenyl-H), 7.25 (d, J = 4.0 Hz, 1H, furan-H)), 7.44 (d, J = 8.0 Hz, 1H, phenyl-H), 7.62 (t, J = 8.0 Hz, 1H, phenyl-H), 7.71 (d, J = 16.0 Hz, 1H, alkene-H), 7.89 (d, J = 8.0 Hz, 1H, phenyl-H), 7.93 (t, J = 8.0 Hz, 1H, phenyl-H), 8.74 (d, 1H, J = 8.0 Hz, phenyl-H). ^{13}C NMR (100 MHz, DMSO-d_6, ppm): δ 158.06, 152.54, 151.95, 150.30, 148.29, 144.63, 142.52, 135.30, 126.05, 124.65, 124.58, 122.10, 121.71, 120.50, 119.13, 118.89, 118.01, 117.47, 117.06, 116.05, 115.46, 110.35, 106.63, 97.53, 59.43. Mass spectrometry (ESI-MS, m/z): $[M + H]^+$ calcd. for $[C_{26}H_{17}N_5O_2 + H]^+$ 432.1461; found 432.1461.

Synthesis of IN1-NO$_2$. I1-NO_2 (1.00 g, 6.13 mmol) and KOH (3.36 g, 60.1 mmol) were dissolved in water (10 mL) under an argon atmosphere at room temperature. Then Me_2SO_4 (3.50 g, 27.8 mmol) was added dropwise. The mixture was refluxed for 1 h under an argon atmosphere. The solution was filtered and the residue was purified by silica gel chromatography (CH_2Cl_2:PE = 1:1) to get the desired product IN1-NO_2 (320 mg). Yield was 29%. ^{1}H NMR (400 MHz, d_6-DMSO, ppm): δ 4.13 (s, 3H, CH_3-H), 7.86 (d, 1H, J = 8.0 Hz, phenyl-H), 8.24 (dd, 1H, J = 8.0 Hz, 4.0 Hz, phenyl-H), 8.39 (s, 1H, imidazole-H), 8.84 (d, 1H, J = 4.0 Hz, phenyl-H). Mass spectrometry (ESI-MS, m/z): $[M + H]^+$ calcd. for $[C_8H_7N_3O_2 + H]^+$ 178.0617; found 178.0631.

Synthesis of IN1-NH$_2$. IN1-NO_2 (200 mg, 1.12 mmol), iron powder (220 mg, 4.00 mmol), and NH_4Cl (150.6 mg, 2.82 mmol) were dissolved in mixture of methanol and water (16 mL, v:v = 3:1) under an argon atmosphere at room temperature. Then the mixture was refluxed for 4 h under an argon atmosphere. The solution was added to ethyl acetate (20 mL) and washed with deionized H_2O (5 mL × 5). The organic layer was dried over anhydrous Na_2SO_4, filtered and concentrated by evaporation. The residue was purified by silica gel chromatography (CH_2Cl_2) to get the product IN1-NH_2 (130 mg). Yield was 79%. ^{1}H NMR (400 MHz, $CDCl_3$, ppm): δ 3.99 (s, 3H, CH_3-H), 6.85 (dd, 1H,

J = 8.0, 4.0 Hz, phenyl-H), 6.90 (s, 1H, phenyl-H), 7.19 (d, J = 8.0 Hz, 1H, phenyl-H), 7.76 (s, 1H, imidazole-H). ^{13}C NMR (100 MHz, $CDCl_3$, ppm): δ 140.08, 135.67, 131.05, 124.89, 118.45, 109.66, 103.48, 35.58. Mass spectrometry (ESI-MS, m/z): $[M + H]^+$ calcd. for $[C_8H_9N_3 + H]^+$ 148.0875; found 148.0915.

Synthesis of IN1-Boc. IN1-NH_2 (400 mg, 2.72 mmol) was dissolved in THF (5 mL) under an argon atmosphere at room temperature. Then $(Boc)_2O$ (594 mg, 2.72 mmol, mixed with 10 mL of THF) was added dropwise. Then the mixture was stirred overnight at room temperature. The solution was added to CH_2Cl_2 (20 mL) and washed with deionized H_2O (20 mL × 5). The organic layer was dried over anhydrous Na_2SO_4, filtered and concentrated by evaporation. The residue was purified by silica gel chromatography (CH_2Cl_2:PE = 1:1) to get the desired product IN1-Boc (500 mg). Yield was 74%. ^{1}H NMR (400 MHz, $CDCl_3$, ppm) δ 1.53 (s, 9H, -$(CH_3)_3$), 4.04 (s, 3H, CH_3-H), 6.60 (s, 1H, phenyl-H), 7.29 (s, 2H, phenyl-H, imidazole-H), 7.81 (s, 1H, phenyl-H), 7.88 (s, 1H, amide-H). ^{13}C NMR (100 MHz, $CDCl_3$, ppm): δ 153.39, 137.09, 132.41, 131.59, 124.16, 120.48, 110.19, 109.16, 80.39, 35.60, 28.40. Mass spectrometry (ESI-MS, m/z): $[M + Na]^+$ calcd. for $[C_{13}H_{17}N_3O_2 + Na]^+$ 270.1218; found 270.1211.

Synthesis of AL-IN1-Boc. IN1-Boc (400 mg, 1.62 mmol), $Cu(OAc)_2$ H_2O (486 mg, 2.40 mmol), furan-2-carbaldehyde (466 mg, 4.86 mmol), pyridine (0.2 mL), and $Pd(PPh_3)_4$ (94 mg, 0.08 mmol) were dissolved in 1,4-dioxane (4 mL) under an argon atmosphere at room temperature. Then the mixture was refluxed for 24 h under an argon atmosphere. The solution was filtered and concentrated by evaporation. The residue was purified by silica gel chromatography (CH_2Cl_2:PE = 1:1) to get the product AL-IN1-Boc (80 mg). Yield was 14%. ^{1}H NMR (400 MHz, $CDCl_3$, ppm): δ 1.55 (s, 9H, -$(CH_3)_3$), 4.11 (s, 3H, NCH_3-H), 6.90 (s, 1H, furan-H), 7.04 (d, J = 4.0 Hz, 1H, furan-H), 7.35 (d, J = 8.0 Hz, 1H, phenyl-H), 7.38 (d, J = 4.0 Hz, 1H, phenyl-H), 7.66 (s, 1H, phenyl-H), 8.05 (s, 1H, amide-H), 9.71 (s, 1H, C*H*O-H). ^{13}C NMR (100 MHz, $CDCl_3$, ppm): δ 177.20, 154.87, 153.34, 151.81, 138.00, 133.80, 133.66, 123.11, 121.46, 121.20, 110.11, 109.69, 108.43, 80.58, 36.12, 28.38. Mass spectrometry (ESI-MS, m/z): $[M + Na]^+$ calcd. for $[C_{18}H_{19}N_3O_4 + Na]^+$ 364.1273; found 364.1273.

Synthesis of DCM-IN1-Boc. AL-IN1-Boc (114 mg, 0.34 mmol), DCM (104 mg, 0.50 mmol), piperidine (0.2 mL), and acetic acid (0.1 mL) were dissolved in Toluene (10 mL) under an argon atmosphere at room temperature. Then the mixture was refluxed for 6 h under an argon atmosphere. The solution was concentrated by evaporation. The residue was purified by silica gel chromatography (CH_2Cl_2) to get the product DCM-IN1-Boc (48 mg). Yield was 27%. ^{1}H NMR (400 MHz, DMSO-d_6, ppm) δ 1.52 (s, 9H, Boc-H), 4.09 (s, 3H, NCH_3-H), 7.04 (d, J = 4.0 Hz, 1H, furan-H), 7.08 (s, 1H, pyran-H), 7.13 (d, J = 16.0 Hz, 1H, alkene-H), 7.20 (d, J = 4.0 Hz, 1H, furan-H), 7.61 (t, J = 8.0 Hz, 2H, phenyl-H), 7.65 (s, 1H, phenyl-H), 7.70 (d, J = 16.0 Hz, 1H, alkene-H), 7.76 (d, J = 8.0 Hz, 1H, phenyl-H), 7.92 (t, J = 8.0 Hz, 1H, phenyl-H), 8.30 (s, 1H, phenyl-H), 8.72 (d, J = 8.0 Hz, 1H, phenyl-H), 9.49 (s, 1H, amide-H). Mass spectrometry (ESI-MS, m/z): $[M + Na]^+$ calcd. for $[C_{31}H_{25}N_5O_4 + Na]^+$ 554.1804; found 554.1797.

Synthesis of DCM-IN1-NH_2. DC-IN1-Boc (40 mg, 0.08 mmol) was dissolved in CH_2Cl_2 (10 mL) under an argon

atmosphere at room temperature. Then trifluoroacetic acid (TFA, 1 mL) was added dropwise. The mixture was stirred overnight at room temperature. The solution was concentrated by evaporation. The residue was purified by silica gel chromatography (CH_2Cl_2) to get the desired product DCM-IN1-NH_2 (20 mg). Yield was 59%. ^{1}H NMR (400 MHz, $CDCl_3$, ppm): δ 4.10 (s, 3H, NCH_3-H), 6.81 (d, J = 16.0 Hz,1H, alkene-H), 6.87 (d, J = 4.0 Hz, 1H, furan-H), 6.90 (s, 1H, phenyl-H), 6.96 (m, 2H, phenyl-H, furan-H), 7.35 (s, 1H, pyran-H), 7.45 (t, J = 8.0 Hz, 2H, phenyl-H), 7.52 (m, 2H, phenyl-H, alkene-H), 7.73 (t, J = 8.0 Hz, 1H, phenyl-H), 8.93 (d, 1H, J = 8.0 Hz, phenyl-H). Mass spectrometry (ESI-MS, m/z): $[M + H]^+$ calcd. for $[C_{26}H_{17}N_5O_2 + H]^+$ 432.1461; found 432.1461.

Synthesis of Br-TB-Boc. 3-bromobenzo[b]thiophen-5-amine (150 mg, 0.66 mmol) was dissolved in THF (5 mL) under an argon atmosphere at room temperature. Then $(Boc)_2O$ (144 mg, 0.66 mmol, mixed with 10 mL of THF) was added dropwise. Then the mixture was stirred overnight at room temperature. The solution was added to CH_2Cl_2 (20 mL) and washed with deionized H_2O (20 mL × 5). The organic layer was dried over anhydrous Na_2SO_4, filtered and concentrated by evaporation. The residue was purified by silica gel chromatography (CH_2Cl_2:PE = 1:1) to get the desired product Br-TB-Boc (100 mg). Yield was 46%. ^{1}H NMR (400 MHz, $CDCl_3$, ppm) δ 1.55 (s, 9H, -$(CH_3)_3$), 6.66 (s, 1H, Ph-H), 7.43 (s, 2H, Ph-H), 7.73 (d, 1H, J = 8.4 Hz, Ph-H), 7.87 (s, 1H, Ph-H), 7.88 (s, 1H, amide-H). ^{13}C NMR (100 MHz, $CDCl_3$, ppm): δ 152.81, 138.23, 136.29, 133.01, 124.56, 124.53, 123.10, 117.73, 112.13, 107.35, 80.83, 28.40. Mass spectrometry (ESI-MS, m/z): $[M + H]^+$ calcd. for $[C_{13}H_{12}NO_2SBr + H]^+$ 325.9850; found 325.9857.

Synthesis of AL-TB-Boc and DC-TB-Boc. Br-TB-Boc (100 mg, 0.31 mmol), (5-formylfuran-2-yl)boronic acid (85 mg, 0.61 mmol), and $Pd(PPh_3)_4$ (35 mg, 0.03 mmol) were dissolved in 1,4-dioxane (3 mL) under an argon atmosphere at room temperature. Then Cs_2CO_3 (398 mg, 1.22 mmol, mixed with 2 mL of water) was added dropwise. Then the mixture was refluxed for 24 h under an argon atmosphere. The solution was filtered. Then, the solution was added to CH_2Cl_2 (20 mL) and washed with deionized H_2O (20 mL × 5). The organic layer was dried over anhydrous Na_2SO_4, filtered and concentrated by evaporation. The solid was dissolved in CH_2Cl_2 (2 mL). After the addition of diethyl ether (10 mL), a yellow crystalline powder precipitated. Then the crude AL-TB-Boc was obtained and used for the next step directly. Mass spectrometry (ESI-MS, m/z): $[M + Na]^+$ calcd. for $[C_{18}H_{17}NO_4S + Na]^+$ 366.0776; found 366.0778. AL-TB-Boc (50 mg, 0.15 mmol), malononitrile (20 mg, 0.30 mmol), and TEA (three drops) were dissolved in $CHCl_3$ (5 mL) under an argon atmosphere at room temperature. Then the mixture was stirred overnight at room temperature. The solution was added to CH_2Cl_2 (20 mL) and washed with deionized H_2O (20 mL × 5). The organic layer was dried over anhydrous Na_2SO_4, filtered and concentrated by evaporation. The residue was purified by silica gel chromatography ((CH_2Cl_2:PE = 1:1) to get desired product DC-TB-Boc (10 mg). Yield was 17%. ^{1}H NMR (400 MHz, $CDCl_3$, ppm): δ 4.56 (s, 9H, CH_3), 7.17 (d, 2H, J = 4.0 Hz, Ph-H), 7.61 (s, 1H, -NH-), 7.73-7.80 (m, 2H, Ph-H), 8.18 (d, 2H, J = 10.8 Hz, Ph-H), 8.86 (s, 1H, Ph-H). Mass spectrometry (ESI-MS, m/z): $[M + Na]^+$ calcd. for $[C_{21}H_{17}N_3O_3S + Na]^+$ 414.0888; found 414.0891.

Synthesis of DC-TB-NH_2. DC-TB-Boc (20 mg, 0.05 mmol) was dissolved in CH_2Cl_2 (10 mL) under an argon atmosphere at room temperature. Then trifluoroacetic acid (TFA, 1 mL) was added dropwise. The mixture was stirred overnight at room temperature. The solution was concentrated by evaporation. The residue was purified by silica gel chromatography (CH_2Cl_2) to get the desired product DC-TB-NH_2 (5 mg). Yield was 33%. ^1H NMR (400 MHz, DMSO-d_6, ppm): δ 6.86 (dd, J = 8.8 Hz, 1H, Ph-H), 7.35 (d, J = 4.0 Hz,1H, Ph-H), 7.64 (d, J = 4.0 Hz,1H, Ph-H), 7.69 (d, J = 1.6 Hz,1H, Ph-H), 7.72 (d, J = 8.4 Hz, 1H, Ph-H), 8.26 (d, J = 9.6 Hz, 2H, Ph-H). ^{13}C NMR (100 MHz, DMSO-d_6, ppm): δ 174.48, 157.03, 146.73, 142.65, 135.92, 129.69, 129.00, 127.99, 123.50, 123.40, 115.15, 114.46, 110.83, 106.39, 72.03. Mass spectrometry (ESI-MS, m/z): $[M + H]^+$ calcd. for $[C_{16}H_9N_3OS + H]^+$ 292.0545; found 292.0546.

Supplementary References

1. Li, Y. *et al.* Ultrasensitive near-infrared fluorescence-enhanced probe for in vivo nitroreductase imaging. *J. Am. Chem. Soc.* **137**, 6407-6416 (2015).
2. Frisch, M. *et al.* Gaussian 16 Rev. A.03 (*Wallingford, CT*, 2016).
3. Zhao, Y. & Truhlar, D. G. The M06 suite of density functionals for main group thermochemistry, thermochemical kinetics, noncovalent interactions, excited states, and transition elements: two new functionals and systematic testing of four M06-class functionals and 12 other functionals. *Theor. Chem. Acc.* **120**, 215-241 (2008).
4. Weigend, F. & Ahlrichs, R. Balanced basis sets of split valence, triple zeta valence and quadruple zeta valence quality for H to Rn: Design and assessment of accuracy. *Phys. Chem. Chem. Phys.* **7**, 3297-3305 (2005).
5. Marenich, A. V., Cramer, C. J. & Truhlar, D. G. Universal solvation model based on solute electron density and on a continuum model of the solvent defined by the bulk dielectric constant and atomic surface tensions. *J. Phys. Chem. B* **113**, 6378-6396 (2009).
6. AVMA Guidelines for the Euthanasia of Animals: 2013 edition (2013).
7. Greene, E. C. Anatomy of the Rat. *Transactions of the American Philosophical Society* **27**, iii-370 (1935).
8. Cox, B. & Emili, A. Tissue subcellular fractionation and protein extraction for use in mass-spectrometry-based proteomics. *Nat. Protoc.* **1**, 1872-1878 (2006).
9. Deen, M. C. *et al.* Selective fluorogenic beta-glucocerebrosidase substrates for convenient analysis of enzyme activity in cell and tissue homogenates. *ACS Chem. Biol.* **15**, 824-829 (2020).
10. Klose J. Preparation of protein samples from mouse and human tissues for 2-D electrophoresis. In: Walker J.M. (eds) The protein protocols handbook. (Springer protocols handbooks. Humana press. 2002).
11. Wu, X. *et al. In vivo* and *in situ* tracking cancer chemotherapy by highly photostable NIR fluorescent theranostic prodrug. *J. Am. Chem. Soc.* **136**, 3579-3588 (2014).
