# Supplementary material for: Fluorescence umpolung enables light-up sensing of N-acetyltransferases and nerve agents
Source: Nat Commun. 2021 Jun 23;12:3869. doi: 10.1038/s41467-021-24187-5 (PMC8222306; doi:10.1038/s41467-021-24187-5)
Supplement: Supplementary file 2 — Reporting summary [file 41467_2021_24187_MOESM2_ESM.pdf]

nature research

Corresponding author(s): Wei-Hong Zhu

Last updated by author(s): May 21, 2021

Reporting Summary

Nature Research wishes to improve the reproducibility of the work that we publish. This form provides structure for consistency and transparency in reporting. For further information on Nature Research policies, see our Editorial Policies and the Editorial Policy Checklist.

Statistics

For all statistical analyses, confirm that the following items are present in the figure legend, table legend, main text, or Methods section.

n/a	Confirmed	
☐	☒	The exact sample size (*n*) for each experimental group/condition, given as a discrete number and unit of measurement
☐	☒	A statement on whether measurements were taken from distinct samples or whether the same sample was measured repeatedly
☐	☒	The statistical test(s) used AND whether they are one- or two-sided<br>*Only common tests should be described solely by name; describe more complex techniques in the Methods section.*
☐	☒	A description of all covariates tested
☐	☒	A description of any assumptions or corrections, such as tests of normality and adjustment for multiple comparisons
☐	☒	A full description of the statistical parameters including central tendency (e.g. means) or other basic estimates (e.g. regression coefficient) AND variation (e.g. standard deviation) or associated estimates of uncertainty (e.g. confidence intervals)
☐	☒	For null hypothesis testing, the test statistic (e.g. *F*, *t*, *r*) with confidence intervals, effect sizes, degrees of freedom and *P* value noted<br>*Give P values as exact values whenever suitable.*
☐	☒	For Bayesian analysis, information on the choice of priors and Markov chain Monte Carlo settings
☐	☒	For hierarchical and complex designs, identification of the appropriate level for tests and full reporting of outcomes
☐	☒	Estimates of effect sizes (e.g. Cohen's *d*, Pearson's *r*), indicating how they were calculated

Our web collection on statistics for biologists contains articles on many of the points above.

Software and code

Policy information about availability of computer code

Data collection	Data of absorption spectra are collected with software of Cary WinUV Scan Application 5.1.0.1016. Data of fluorescent spectra are collected with software of Cary Eclipse Scan Application 1.2(147). Data of cell imaging is collected with software of Leica Application Suite X 1.1.0.12420. Data of animal imaging is collected with the software of a PerkinElmer IVIS Lumina Kinetic Series III imaging system. The PerkinElmer IVIS Lumina Kinetic Series III imaging system software: Living Image 4.5 (64-bit).
Data analysis	Data analysis of absorption spectra use OriginPro 2015 (64-bit) Sr2 b9.2.272 and Prism 7 for Windows 7.00. Data analysis of fluorescent spectra use OriginPro 2015 (64-bit) Sr2 b9.2.272 and Prism 7 for Windows 7.00. NMR data was processed with MestReNova (Mestrelab research, 11.0.3-18688) or TopSpin (Bruker, 4.0.6). Data analysis of cell imaging and MTT assay uses OriginPro 2015 (64-bit) Sr2 b9.2.272, Image 1.47f and Prism 7 for Windows 7.00. Data analysis of animal imaging uses a PerkinElmer IVIS Lumina Kinetic Series III imaging system. The PerkinElmer IVIS Lumina Kinetic Series III imaging system software: Living Image 4.5 (64-bit). M06-2X/Def2-SVP and CAM-B3LYP/def2SVP calculations were carried out using Gaussian 16 A. Docking calculation was carried out using AutoDock 4.2.6. Data analysis of M06-2X/Def2-SVP and CAM-B3LYP/def2SVP calculations and Docking calculation use OriginPro 2015 (64-bit) Sr2 b9.2.272 and Prism 7 for Windows 7.00.

For manuscripts utilizing custom algorithms or software that are central to the research but not yet described in published literature, software must be made available to editors and reviewers. We strongly encourage code deposition in a community repository (e.g. GitHub). See the Nature Research guidelines for submitting code & software for further information.

Data

Policy information about availability of data

All manuscripts must include a data availability statement. This statement should provide the following information, where applicable:

- Accession codes, unique identifiers, or web links for publicly available datasets
- A list of figures that have associated raw data
- A description of any restrictions on data availability

The X-ray crystallographic coordinates for structures reported in this study have been deposited at the Cambridge Crystallographic Data Centre (CCDC), under deposition numbers CCDC-2041874, CCDC-2041875, CCDC-2041872, CCDC-2041871, CCDC-2041873, and CCDC-2041870. These data can be obtained free of charge from The Cambridge Crystallographic Data Centre via www.ccdc.cam.ac.uk/data_request/cif. The source data underlying Figures 4c, 6d, 6e, 6g, 6l, 6k, and Supplementary Figures 6a, 6b, 6d, 6e, 7, 8, 10, 12b(Inset), 15, 16a are provided as a Source Data file. All other data are available from the corresponding author. Source data are provided with this paper.

Field-specific reporting

Please select the one below that is the best fit for your research. If you are not sure, read the appropriate sections before making your selection.

☒ Life sciences ☐ Behavioural & social sciences ☐ Ecological, evolutionary & environmental sciences

For a reference copy of the document with all sections, see nature.com/documents/nr-reporting-summary-flat.pdf

Life sciences study design

All studies must disclose on these points even when the disclosure is negative.

Sample size	Sample size was determined as n ≥ 3. As the nature of the experiments were exploratory, the number of individuals in each experiment was determined to validate technological advance and was not used to validate a biological hypothesis.
Data exclusions	No data were excluded from the data analysis.
Replication	The cell and animal imaging was repeated independently for 3 times with similar results.
Randomization	Allocation was random.
Blinding	The investigators were blinded to group allocation during data collection and analysis.

Reporting for specific materials, systems and methods

We require information from authors about some types of materials, experimental systems and methods used in many studies. Here, indicate whether each material, system or method listed is relevant to your study. If you are not sure if a list item applies to your research, read the appropriate section before selecting a response.

Materials & experimental systems

n/a	Involved in the study	
☒	☐	Antibodies
☐	☒	Eukaryotic cell lines
☒	☐	Palaeontology and archaeology
☐	☒	Animals and other organisms
☒	☐	Human research participants
☒	☐	Clinical data
☒	☐	Dual use research of concern

Methods

n/a	Involved in the study	
☒	☐	ChIP-seq
☒	☐	Flow cytometry
☒	☐	MRI-based neuroimaging

Eukaryotic cell lines

Policy information about cell lines

Cell line source(s)	were purchased from the Institute of Cell Biology (Shanghai, China).
Authentication	Authentication testing of Hep-G2 and HeLa cell lines have been performed by shanghai Biowing Applied Biotechnology Co.Ltd via STR profiling. STR profiles match the standards recommended for Hep-G2 and HeLa cell lines authentication.
Mycoplasma contamination	The cell lines were not tested for mycoplasma contamination.
Commonly misidentified lines (See ICLAC register)	There is no commonly misidentified cell lines in the study.

Animals and other organisms

Policy information about studies involving animals; ARRIVE guidelines recommended for reporting animal research

Laboratory animals	The 3-4-week-old female BALB/cA nude mice were purchased from Shanghai Genechem Co. Ltd. and maintained under standard conditions. The animals were housed in sterile cages within laminar airflow hoods at 24 °C, 45-65% humidity in a specific pathogen-free room with a 12-h light/12-h dark schedule and fed autoclaved chow and water ad libitum.
Wild animals	The study did not involve wild animals.
Field-collected samples	The study did not involve samples collected from the field.
Ethics oversight	The procedures for care and use of animals were approved by the East China University of Science and Technology Animal Studies Committee, and all applicable institutional and governmental regulations concerning the ethical use of animals were followed.

Note that full information on the approval of the study protocol must also be provided in the manuscript.
